# Molecular Landscape of Tourette’s Disorder

**DOI:** 10.3390/ijms24021428

**Published:** 2023-01-11

**Authors:** Joanna Widomska, Ward De Witte, Jan K. Buitelaar, Jeffrey C. Glennon, Geert Poelmans

**Affiliations:** 1 Department of Cognitive Neuroscience, Donders Institute for Brain Cognition and Behaviour, Radboud University Medical Center, 6525 GA Nijmegen, The Netherlands; 2 Department of Human Genetics, Radboud University Medical Center, 6525 GA Nijmegen, The Netherlands; 3 Conway Institute of Biomolecular and Biomedical Research, School of Medicine, University College Dublin, D04 V1W8 Dublin, Ireland

**Keywords:** Tourette’s disorder, genetics, tissue/cell type specificity analyses, functional enrichment analyses, genetic sharing analyses, molecular landscape, drug targets

## Abstract

Tourette’s disorder (TD) is a highly heritable childhood-onset neurodevelopmental disorder and is caused by a complex interplay of multiple genetic and environmental factors. Yet, the molecular mechanisms underlying the disorder remain largely elusive. In this study, we used the available omics data to compile a list of TD candidate genes, and we subsequently conducted tissue/cell type specificity and functional enrichment analyses of this list. Using genomic data, we also investigated genetic sharing between TD and blood and cerebrospinal fluid (CSF) metabolite levels. Lastly, we built a molecular landscape of TD through integrating the results from these analyses with an extensive literature search to identify the interactions between the TD candidate genes/proteins and metabolites. We found evidence for an enriched expression of the TD candidate genes in four brain regions and the pituitary. The functional enrichment analyses implicated two pathways (‘cAMP-mediated signaling’ and ‘Endocannabinoid Neuronal Synapse Pathway’) and multiple biological functions related to brain development and synaptic transmission in TD etiology. Furthermore, we found genetic sharing between TD and the blood and CSF levels of 39 metabolites. The landscape of TD not only provides insights into the (altered) molecular processes that underlie the disease but, through the identification of potential drug targets (such as FLT3, NAALAD2, CX3CL1-CX3CR1, OPRM1, and HRH2), it also yields clues for developing novel TD treatments.

## 1. Introduction

Tourette’s disorder (TD) is a childhood-onset neurodevelopmental disorder characterized by multiple motor and vocal tics lasting more than one year. Tics are generally preceded by premonitory urges, peak in severity between the ages of 10 and 12, fluctuate over time, and, in most cases, show improvement by late adolescence or early adulthood. TD affects approximately 1% of the general population, is more prevalent in males and is often associated with other neuropsychiatric comorbidities, including attention-deficit/hyperactivity disorder (ADHD), obsessive compulsive disorder (OCD), autism spectrum disorders (ASDs), anxiety, and depression [1,2]. TD is a highly familial and heritable disorder [3]. Furthermore, TD is thought to be a complex disease resulting from interactions between multiple genetic and environmental risk factors, although the etiology and pathogenesis of TD have not yet been (fully) elucidated. Genetic studies have suggested that both common genetic variants with small effects and rare variants with larger effects contribute to TD risk, although small sample sizes have hindered the discovery of genome-wide significant signals. Various biological effects of the genes (and their encoded proteins) that have been associated with TD, including alterations in the histaminergic pathway, synaptic transmission, cell adhesion, and mitochondrial function, highlight the complexity of the disorder [4,5]. Moreover, environmental factors, such as pre-, peri-, and postnatal events, psychological stress, and infections, could not only contribute to gene-environment interactions but also affect the development, course, and severity of TD symptoms [6,7]. Neurobiologically, TD appears to involve abnormalities in the development, structure and function of cortico-striato-thalamo-cortical (CSTC) circuits associated with motor and behavioral control and with impaired signaling of multiple modulatory neurotransmitters, especially dopamine [8].

As for treating TD, current therapies—including psychoeducation, behavioral interventions and medication, such as atypical antipsychotics—may partly ameliorate symptoms [9,10,11,12]. However, inadequate control of tics and the occurrence of adverse side effects hinder the treatment of TD. Therefore, novel strategies are required to enhance our understanding of the molecular basis of this disorder, which could in turn provide clues for the development of (more) effective treatments. Previous studies have shown that drug candidates are more likely to pass clinical trials and be approved for patients if they target genes linked to human disease [13,14], highlighting the importance of human genetics in drug target identification. In addition, considering that well-powered studies of candidate gene hypotheses for other complex traits, e.g., schizophrenia [15,16], showed that previously reported positive findings were highly likely to be false positives, we decided to focus on omics datasets.

More specifically, we applied and extended the approach that we used before to build so-called ‘molecular landscapes’ of complex neuropsychiatric diseases, including ADHD [17], ASDs [18], OCD [19], and Parkinson’s disease (PD) [20]. In short, we first compiled a comprehensive list of candidate genes that are associated with TD through one or more types of omics data. These data primarily included genomic data (different types of common and rare genetic variants) and were corroborated by epigenomic data (DNA methylation) and transcriptomic data (differential gene/mRNA expression in blood and brain). To identify the molecular mechanisms that are affected in TD, we then performed tissue/cell type specificity and functional enrichment analyses of the TD candidate genes. As biofluid levels of many metabolites represent ‘intermediate phenotypes’ (that link genetic or environmental risk factors to a disease) and the variation in these metabolite levels is at least in part attributable to genetic factors [21,22,23], we also used genomic data to investigate the extent and direction of genetic overlap between TD and the levels of a large number of blood and cerebrospinal fluid (CSF) metabolites. Subsequently, we applied additional selection criteria—that reflected the amount of independent omics evidence—to the list of TD candidate genes, resulting in ‘prioritized’ TD candidate genes and candidate genes for which less omics evidence implicating them in TD etiology was available. Lastly, we built a molecular landscape of TD through integrating the results from the tissue/cell type and functional enrichment analyses with an elaborate literature search for interactions between the proteins encoded by the TD candidate genes and the metabolites implicated through the genetic overlap analyses and other metabolome/microbiome studies. The resulting TD landscape provides insights into the (altered) molecular processes that underlie the disease as well as potential drug targets that could be further developed into treatments. 

## 2. Results

### 2.1. Input Omics Datasets and Candidate Genes

Based on the literature search and our analyses of the TD GWAS data, we compiled a list of TD candidate genes from the single-omics studies of TD (i.e., genomics, transcriptomics, epigenomics, metabolomics, and microbiomics). We provide the characteristics of the included studies in Table S1 and below, we briefly describe the included studies. 

Based on the type of omics evidence they provide, we classified the studies as guiding (genomics studies), corroborating (epigenomics and transcriptomics studies) and additional (metabolomics and microbiome) studies. For the genomics data, we compiled a list of TD candidate genes from studies of rare genetic variants/events—(i) eight chromosomal rearrangements studies [24,25,26,27,28,29,30,31] (the main list includes 15 genes and the extended list consists of 23 genes), (ii) fifteen single-nucleotide variation (SNV) studies [27,32,33,34,35,36,37,38,39,40,41,42,43,44,45] (the main list includes 134 genes and the extended list consists of 846 genes), and (iii) eleven copy number variations (CNV) studies [37,44,46,47,48,49,50,51,52,53,54] (the main list includes 52 genes, and the extended list consists of 956 genes)—and studies of common genetic variants (of single-nucleotide polymorphisms or SNPs), i.e., genome-wide association studies (GWASs). The GWAS-derived genes include the results from our own unpublished analyses of the summary statistics data from the TD GWAS by Yu et al. [55], i.e., 113 genes from the MAGMA analysis, 224 genes from the FUMA analysis, and 143 genes from the TWAS analysis. In addition, the GWAS-derived candidate genes include published results of (other) studies using TD GWASs, including cross-disorder studies and annotation of the GWAS by Yu et al. from the GWAS Catalog [56,57,58,59,60], as well as the preliminary MAGMA results from the newest TD GWAS (available as a preprint on medRxiv at the time of analysis) [61].

Corroborating evidence for the genomic-studies-derived genes was assembled from two epigenome-wide association studies (EWASs) that investigated DNA methylation in the peripheral blood of TD patients versus controls [62,63] (the main list includes 71 genes and the extended list consists of 8 genes), and transcriptomic studies in the brain (postmortem, in the striatum of medicated TD patients vs. controls) [64] (957 genes) and blood (medicated [65,66] and unmedicated [67,68] TD patients versus control). We could not find any published proteomic studies of TD.

As for additional evidence, we included findings from three studies that investigated metabolomic changes in the plasma of TD patients [69], serum of PANS patients [70], and urine in a case study of PANDAS-associated tics [71], and from microbiome studies in children with tic disorder [72] and in PANS/PANDAS patients with tics [73]. These results were used as supportive evidence for the metabolites linked to TD through the PRS-based analyses (see below).

We combined the lists of genes implicated through the abovementioned genomic, epigenomic and transcriptomic studies along with their functional annotations into one table (Table S2). In total, we compiled a list of 872 TD candidate genes implicated in TD through the genomic studies (guiding evidence), and this list was subsequently used in tissue and cell specificity and functional enrichment analyses.

### 2.2. Tissue and Cell Type Specificity

We performed specificity analyses to identify the tissues and cell types with enhanced expression of TD-associated genes. We separately analyzed the genes associated with TD based on all types of genomic data and on the postmortem brain transcriptomic data to identify tissues and cell types that contribute to the development of TD and that are particularly affected by lifelong TD, respectively.

#### 2.2.1. Genomic Data

In the Tissue Specific Expression Analysis (TSEA), we found that genes preferentially expressed in 2 out of the 25 human tissues tested were significantly enriched (pSI < 0.05, FDR *p*-value < 5 × 10^−2^, see Materials and Methods) within the 872 TD candidate genes: the brain as a whole (FDR *p*-value = 9.17 × 10^−9^, 148 genes) and the pituitary (FDR *p*-value = 1.30 × 10^−5^, 114 genes; of note, 74 genes were enriched in both the brain and pituitary). We also observed strong expression enrichment in the brain and, to a lesser extent, the pituitary at the more stringent pSI cutoff of 0.01 (brain: FDR *p*-value = 2.73 × 10^−5^, 81 genes; pituitary: FDR *p*-value = 1.3 × 10^−2^, 46 genes), which indicated an expression enrichment of specific TD candidate genes in the brain (and pituitary) (File S1). 

In the analysis of human spatiotemporal brain gene expression data (6 brain regions across 10 developmental periods), we found that TD gene expression was enriched in seven spatiotemporal coordinates, involving 4 brain regions during specific developmental periods: (I) cerebellum: early fetal period (FDR *p*-value = 2.8 × 10^−2^, 53 genes); (II) cortex: early mid-fetal period (FDR *p*-value = 4 × 10^−3^, 51 genes), neonatal period/early infancy (FDR *p*-value = 5 × 10^−3^, 33 genes), adolescence (FDR *p*-value = 1.4 × 10^−2^, 34 genes), young adulthood (FDR *p*-value = 5.021 × 10^−4^, 48 genes); (III) striatum: early mid-fetal period (FDR *p*-value = 4.3 × 10^−2^, 36 genes); (IV) thalamus: neonatal period/early infancy (FDR *p*-value = 2.1 × 10^−2^, 46 genes). We also observed a trend-significant enrichment of the late mid-fetal period in the cortex (FDR *p*-value = 5.2 × 10^−2^, 36 genes) and mid–late childhood in the cerebellum (FDR *p*-value = 7.9 × 10^−2^, 53 genes) (File S2). 

Taking into account the substantial cellular heterogeneity in brain tissue, we then used single-cell data from adult mice to identify individual candidate cell populations that are likely to be affected in TD. In the Cell-Specific Expression Analysis (CSEA), we observed a trend-significant enrichment of two cell populations: Drd2+ medium spiny neurons (MSNs) of the striatum (33 genes) and layer 6 corticothalamic neurons (Ntsr+, 21 genes), although these results did not pass our threshold for significance (FDR *p*-value = 1.6 × 10^−1^ for both cell types) (File S3). 

#### 2.2.2. Brain Transcriptomic Data

Alterations of gene expression from a complex mix of cells, such as those from brain tissue, may represent changes in the (relative) cellular composition of the tissue [74]. To this end, we also applied the CSEA method to the transcriptomic data from the postmortem striatum of TD patients [64] to infer the cellular fingerprint of a lifelong disease. Among the striatal cell types assessed by CSEA, genes downregulated in TD striatum were over-represented in the expression profiles of cholinergic interneurons (FDR *p*-value < 5 × 10^−2^ across all pSI thresholds) and, to a lesser extent, Drd1+ MSNs (FDR *p*-value = 6 × 10^−2^), indicating a loss and/or reduced function of these cell types. Of note, the enrichment of cholinergic interneurons was also significant for the basal forebrain, which is consistent with their restricted distribution in the central nervous system (CNS), i.e., with these cell types being concentrated in these particular brain regions (the basal forebrain, caudate and putamen) [75]. Furthermore, genes downregulated in the TD striatum were also enriched for genes that are highly expressed in cortical cells, i.e., layer 6 corticothalamic neurons (Ntsr+ neurons), layer 5 pyramidal neurons projecting to the thalamus, spinal cord and striatum (Glt25d2 neurons), and cortical neurons that express the prepronociceptin gene (Pnoc+ neurons) (File S4). The analysis of the WGCNA module that was enriched for downregulated genes further confirmed an overabundance of genes that are highly expressed in interneurons, and it provided evidence for an involvement of Drd2+ MSNs (FDR *p*-value < 5 × 10^−2^ across all pSI thresholds) (File S5). Lastly, the analysis of upregulated genes revealed an overrepresentation of immunity-related cell types (in the cortex) and glial cells (in the cerebellum and cortex) (File S6), while the WGCNA module analysis yielded a specific enrichment of immune cells (File S7).

### 2.3. Functional Enrichment Analyses

We used Ingenuity Pathway Analysis (IPA) to identify canonical pathways, diseases/biological functions and upstream regulators that are enriched within the 872 TD candidate genes. We provide the full results of all analyses performed with IPA in Table S3, while below, we describe the most significant findings (i.e., with FDR *p*-value < 5 × 10^−2^). 

The canonical pathway analysis in IPA identified two signaling pathways that were significantly enriched within the 872 genes: ‘cAMP-mediated signaling’ and ‘Endocannabinoid Neuronal Synapse Pathway’ (Table 1). Three genes—*ADCY2*, *MAPK3*, and *PRKAR2A*—were implicated in both pathways, suggesting a (partial) shared underlying biology.

In the diseases and biofunctions analysis, IPA identified significant enrichment of 71 functional annotations contained within two functional categories: Molecular and Cellular Functions and Physiological System Development and Function (Table 2). Most of the enriched functions are linked to the development and function of the nervous system and include many overlapping genes (Table S3b). The Diseases and Disorders category was highly enriched for cancer-related diseases (Table S3b). This result is partly driven by the inclusion in IPA of findings from the COSMIC and ClinVar projects, which identified many associations between genes and various cancers, and genes involved in normal biological processes are impacted when these functions are dysregulated by cancer. After filtering the results to exclude cancer, we found a significant association (FDR *p*-value < 5 × 10^−2^) with 119 disease annotations falling into several higher-level categories, including ‘Neurological Disease’ (54 annotations), ‘Psychological Disorders’ (30), ‘Developmental Disorder’ (19), ‘Hereditary Disorder’ (17), ‘Cardiovascular Disease’ (9), ‘Skeletal and Muscular Disorders’ (9), ‘Gastrointestinal Disease’ (7), ‘Infectious Disease’ (5), and ‘Inflammatory Disease’ (4). 

In the upstream regulator analysis, none of the identified upstream regulators remained significant after correction for multiple testing (Table S3c). That being said, the top regulators at a suggestive *p*-value threshold (uncorrected *p*-value < 1 × 10^−2^) include—among ‘Drugs and Chemicals’—molybdenum disulfide (chemical reagent), topotecan (chemical drug), GnRH analog (biologic drug), lipoxin LXA4 (endogenous chemical), and—among ‘Genes, RNAs and Proteins’—NEDD4 (enzyme), OPRM1 (G-protein coupled receptor), ANGPT2 (growth factor), CLCA2 (ion channel), and TEAD4 (transcription regulator).

### 2.4. Shared Genetic Etiology Analyses with Levels of Blood and Cerebrospinal Fluid Metabolites

#### 2.4.1. Polygenic Risk Score (PRS)-Based Analyses

We conducted PRS-based analyses to investigate the presence and extent of genetic overlap between TD and metabolite concentrations in blood and/or CSF. After Bonferroni correction for the number of tests performed, we identified significant associations between TD and the levels of 37 blood metabolites (out of the 993 blood metabolites tested) and 2 CSF metabolites (out of the 338 CSF metabolites tested). The results for these metabolites are presented in Table 3, along with the superpathway and pathway annotations (where applicable). Genetic variants associated with TD explained up to 2.08% of the genetically determined variation in the levels of the 37 significant blood metabolites, and up to 9.90% of the variation in the levels of the two significant CSF metabolites. The complete results of the PRS-based analyses for the blood and CSF metabolites are provided in Table S4a,b.

#### 2.4.2. SNP Effect Concordance Analysis (SECA)

Through performing SECA for the significantly associated metabolites from the PRS-based analyses, we found a significant genetic concordance between TD and the levels of all 39 metabolites (Table 3). Among these, 24 blood metabolites showed positive concordance, indicating that genetic variants associated with TD also convey genetic risk to increased blood levels of these metabolites. The remaining 13 blood metabolites—including indoxyl sulfate, homocitrulline, pyridoxate, myo-inositol, triacylglycerols—and the two CSF metabolites—*N*-acetyl-aspartyl-glutamate (NAAG) and butyrate—showed negative concordance with TD, implying that genetic variants associated with TD also convey genetic risk to decreased blood/CSF levels of these metabolites.

### 2.5. Molecular Landscape of TD 

**Figure 1:**
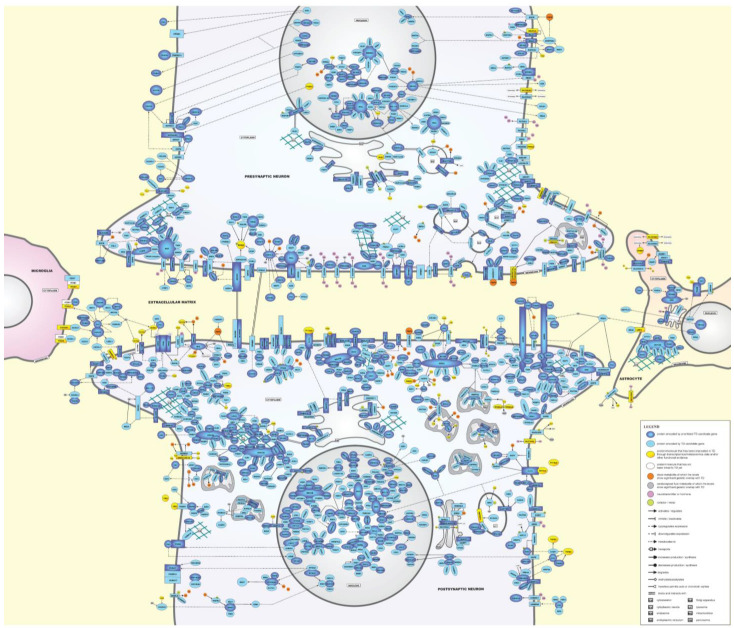
Molecular landscape of TD. In this landscape, the interactions between the key proteins/molecules/metabolites implicated in TD in pre- and postsynaptic neurons, astrocytes and/or microglial cells are shown. In Table S5, all interactions between the landscape proteins/molecules/metabolites are provided. In File S8, we provide a pdf version of Figure 1 that will allow interested readers to look up proteins and molecules in the landscape through using the search function.

Through the approach described in the Materials and Methods and by integrating the results from the tissue/cell type specificity and functional enrichment analyses with the literature search for interactions between the proteins encoded by the 872 TD candidate genes and the metabolites implicated through the PRS-based analyses, we built a molecular landscape of TD (Figure 1). The landscape is located in the synapse, where presynaptic and postsynaptic neurons interact with astrocytes, microglial cells and the extracellular matrix (ECM), together forming a structure referred to in the literature as the pentapartite synapse [81,82]. For building the landscape, we focused on the 239 proteins encoded by the prioritized TD candidate genes (see Materials and Methods)—that are dark blue in Figure 1—and their interactions. In addition, if they interacted with at least one of the dark blue proteins, we added some of the remaining proteins encoded by the remaining TD candidate genes for which there was less omics evidence available (see Materials and Methods)—and these proteins are light blue in Figure 1. In total, this amounted to 197 (unique) dark blue proteins and 276 (unique) light-blue proteins that are shown in the landscape. The landscape also includes 42 yellow proteins/molecules that have been implicated in TD through transcriptomics/metabolomics data and/or other functional evidence. Lastly, the 11 blood and 2 CSF metabolites of which the levels were found to show significant genetic overlap with TD are indicated in orange and grey, respectively. All interactions between the landscape proteins/molecules can be found—with their corresponding literature references—in Table S5, but below, we have provided a description of the main processes in the landscape, with the key implicated proteins/molecules/metabolites in bold and by part of the neuronal cells where these processes/cascades are mainly taking place. 

#### 2.5.1. Description of the TD Landscape

##### Presynaptic and Postsynaptic Neurons

- Extracellular matrix (ECM)

Synapses are enwrapped by a layer of extracellular matrix (ECM), which is important for (shaping and maintaining) synaptic morphology and function. The ECM of the brain consists of non-fibrous proteins such as glycoproteins, matricellular proteins (such as periostin and tenascins), enzymes that regulate ECM deposition and degradation, and fibrous/structural proteins (such as collagens and laminins) [83]. The ECM proteins can modulate the activity or bioavailability of extracellular signaling molecules, such as growth factors, cytokines, chemokines, and extracellular enzymes, and/or bind directly to cell surface receptors to regulate cellular functions. ECM components are synthesized intracellularly in glia and neurons and secreted into the ECM, where they aggregate with the existing matrix, fill the synaptic cleft, and interact with cell surface receptors. Furthermore, the ECM is also involved in the exchange of nutrients and metabolites between the CNS and systemic circulation [84]. 

First, **THBS1**, an adhesive glycoprotein that is downregulated by **indoxyl sulfate** (**IS**), binds and interacts with multiple other landscape proteins, both in the ECM and membrane. Another ECM glycoprotein involved in—among other functions—cell adhesion is **FN1**, and this protein is upregulated by palmitic acid and (also) has very many interactions with other landscape proteins in ECM and membrane. In addition, **POSTN** (**periostin**) is an ECM protein that plays a role in cell adhesion and ECM remodeling by regulating the expression of landscape proteins—including **FN1**—and interacting with membrane proteins. The tenascin **TNN** is involved in neurite outgrowth through binding the **ITGA4-ITGB1-complex** in the membrane (of microglial cells). Moreover, enzymes that regulate ECM function are the peptidase **DPP4** that (also) binds **FN1**, the protease **HTRA3** that cleaves **FN1**, and **LOLX1**, an oxidase that catalyzes the formation of crosslinks in collagen and elastin fibers. In addition, **RELN** (**reelin**) is a serine protease that regulates many functions, e.g., neuronal adhesion, neuronal migration, neurite outgrowth and synaptic plasticity. **RELN** expression is regulated by the transcription factors **NPAS3** and **TBR1** as well as the membrane receptor **FFAR3** (see below), while **butyric acid** decreases its acetylation and demethylation. Moreover, **RELN** degrades **FN1** and binds/signals through the membrane protein **LRP8** (in astrocytes). Fibrous/structural landscape proteins include several collagen proteins (**COL4A2**, **COL5A1**, **COL6A3**, and **COL8A1**) that regulate synaptogenesis and neuronal cell adhesion, and the laminin **LAMA5**.

Other ECM landscape proteins include the vasoactive peptide **ADM** (that downregulates **FN1**), lipid transport-regulating **APOM** (that downregulates **FN1** and is downregulated by **palmitic acid**), **GCG** (which is a precursor that can be cleaved into multiple peptides, one of which downregulates **FN1**), **CLCA2** (a chloride channel accessory protein that regulates the expression of **CDKNA1** and **FN1**), **CX3CL1** (a membrane protein that is cleaved by the neuronal membrane enzyme **ADAM10** into a soluble form that is a ligand for both **CX3CR1** and the **ITGA4-ITGB1-complex** in the membrane of microglial cells), the interleukins **IL17A** and **IL31**, **LTBP1**—a key regulator of transforming growth factor beta proteins such as **TGFB1** and **TGFB3**—and **NOTCH2NLA**, a component of the NOTCH signaling pathway that regulates neuronal differentiation and can also be located in the cytoplasm. Other ECM proteins with a role in regulating neuronal differentiation and function are **NXPH1** (that binds and interacts with **NRXN1** in the presynaptic membrane), the protease inhibitor **SERPINE2** (that, among other interactions, downregulates the expression of the cytokine **TNFB**), the nerve growth factor **VGF** (that, e.g., regulates **CHGB**, a neuroendocrine secretory granule protein), and members of the WNT protein family (**WNT1**, **WNT3**, **WNT5A**, and **WNT10B**) that modulate many processes such as neuronal differentiation and migration, dendrite development, synaptogenesis, adult neurogenesis, and neural plasticity (summarized in [85]).

- Cell membrane

Neuronal cell membrane proteins connect extracellular and intracellular signaling cascades and largely determine a neuronal cell’s capacity to communicate and interact with its environment. Different types of cell membrane proteins can be discerned, including enzymes, receptors, ion channels, transporters, cell adhesion-regulating proteins, and other membrane proteins [86].

First, a number of landscape proteins are membrane-located enzymes, such as **DPEP2** (involved in the metabolism of **arachidonic acid**), **FKBP11** (that regulates protein folding), **MARK3** (involved in the phosphorylation of **MAPT**), and **NAALAD2**, an enzyme that is expressed in neuronal and astrocytic membranes and regulates **glutamate** synthesis, see below. In addition, **DAGLA** is a membrane-located enzyme that is involved in the metabolism of **arachidonic acid** and **stearic acid**. **DAGLA** also complexes with the presynaptic transporter **SLC6A4** (see below) and with the postsynaptic density scaffolding protein **HOMER2**. Furthermore, like the related protein **ZDHHC8** in the Golgi membrane (see below), the membrane-located enzyme **ZDHCC17** transfers **palmitic acid** onto target proteins, and it also complexes with **TNFB** and other landscape proteins, including the membrane proteins **LMBR1L** and **TMEM100B**. 

As for receptors, different functional classes are located in the (neuronal) cell membrane. In this respect, several landscape proteins are G protein-coupled receptors (GPCRs). GPCRs are highly expressed throughout the brain and regulate synaptic transmission and plasticity [87]. After being bound and activated by their ligands, GPCRs regulate downstream signaling through stimulatory G-proteins—**CRHR1**, the receptor for the hormone **CRH** and in this way a major regulator of the hypothalamic–pituitary–adrenal (HPA) cascade, and **HRH2**, a receptor of histamine that interacts with the TD-linked enzyme **histidine decarboxylase** (**HDC**) (see below) and regulates **arachidonic acid** production—and inhibitory G proteins. Examples of the latter type of GPCRs include **CX3CR1** (a membrane receptor that is highly expressed in microglial cells and that is activated and involved in regulating the immune response through binding its ligand **CX3CL1**, which itself also signals through the membrane **ITGA4-ITGB1-complex**) and **DRD2**, a **dopamine** that receptor interacts with, regulates, or is regulated by multiple landscape proteins. Other membrane GPCRs that signal through inhibitory G-proteins are **FFAR3** (that is activated by **butyrate** and downregulates the expression of landscape proteins such as **RELN** and the potassium channel (see below) **KCNH5**), as well as **FPR1**, **FPR2** and **FPR3**, chemokine receptors that form a functional complex, regulate inflammation, and are regulated by the extracellular proteins **ANXA1** and arachidonic acid metabolite LXA4 and by intracellular **BHLE40** and **COP1**. Furthermore, opioid receptors representing the μ, δ, and κ families—encoded by the **OPRM1**, **OPRD1** and **OPRK1** genes, respectively—interact with each other (with **OPRM1** signaling through both stimulatory and inhibitory G-proteins) and multiple landscape proteins in the cell membrane (**CD302**, **DRD2**, **EGFR**, and **SLC6A4**), extracellular space (**ADM**, **FN1**) and cytoplasm (**CKB**, **CRKL**, **HSP90AA1**, **NCL**, **PI4KA**, and **TLN2**). Lastly, the **PROK2-PROKR2 complex** induces the production of gonadotropin-releasing hormone (GnRH), which has been linked to TD (see below). Other non-GPCR landscape membrane receptors include the kinase receptors **EGFR**—that interacts with many landscape proteins, including being inhibited by the cytoplasmic protein **ERRFI1**—and **FLT3** that, when bound/activated by the cytokine **FLT3LG**, regulates the phosphorylation of **MAPK3** and **MAPT**. Moreover, **FLT3** regulates the expression of nuclear **EXCC6**, cytoplasmic **PIM1**, and lysosomal **MPO**, and is degraded by **RNF115**. Other membrane receptors in the landscape include **IL17RB**—which forms a functional complex with IL17RA that has **IL17A** as its ligand—and **PLA2R1**, a receptor of phospholipase A2 (not shown) that upregulates the expression of the mitochondrial enzyme **MGST1** (see below). Furthermore, **PTPRU** is a (pre) synaptic phosphatase receptor involved in the development and maintenance of dopaminergic neurons [88], while **NOTCH1** is a (pre)synaptic membrane receptor that interacts with multiple landscape proteins and, upon ligand activation, the notch intracellular domain (**NOTCH1-ID**) is released into the cytoplasm and subsequently the nucleus, where it functions as a transcription factor through, e.g., interacting with **RERE** (see below). Moreover, a number of landscape proteins are (subunits of) neurotransmitter receptors that, when activated through neurotransmitter binding, function as ligand-gated ion channels (see below): the **acetylcholine** receptor subunits **CHRNA7** and **CHRNB4**, the **GABA** (**γ-aminobutyric acid**) receptor subunits **GABRA2**, **GABRB3** and **GABRG1**, and the NMDA **glutamate** receptor subunits **GRIN1**, **GRIN2A**, **GRIN2B** and **GRIN3A**. Lastly, **SELE** (**selectin-E**) is a (pre-or post)synaptic membrane receptor that is involved in immunoadhesion and that is (also) highly expressed in brain vascular endothelial cells. **SELE** binds its membrane-located ligand extracellular **SELPLG**—which leads to its dephosphorylation—and its expression is regulated by intracellular **ENO1**, **ESR1**, **indoxyl sulfate**, **MAPK3K4**, **MAPK3**, **MRTFA** and **RCAN1**, membrane-located **NOTCH1**, and secreted, extracellular **SERPINE2**. 

In addition, several membrane-located ion channels operate in the landscape. A first group of ion channels are the neurotransmitter receptors that, upon activation, function as ligand-gated ion channels (see above). Secondly, the landscape contains multiple (subunits of) voltage-gated ion channels that mediate the transport of (univalent and divalent) ions into neuronal cells, including the calcium channel subunit **CACNA1D**, the chloride channel subunit **CLCN2** (which is activated by **arachidonic acid**), the sodium channel subunit **SCN5A**, and the potassium channel subunits **HCN1**, **HCN4**, **KCNH3**, **KCNH5**, **KCNJ11** and astrocytic **KCNK1**, with the latter also being regulated by the cytoplasmic enzyme **SENP1** (see below).

Members of the ATP-binding cassette (ABC) family of transporters—**ABCA7** and **ABCG8** (which itself forms a functional complex with extracellular **APOM** (see above))—play a role in lipid homeostasis. In addition, **ABCC1** mediates the export of organic anions and many drugs from the cytoplasm. In astrocytes, the expression of **ABCG8** is regulated by **NR1H2**, whereas **ABCC1** forms a complex with the multifunctional membrane protein **LMBR1L** (see below). Other landscape transporters belong to the solute carrier family of proteins, including the (post)synaptic sodium/bicarbonate cotransporter **SLC4A10** that regulates intracellular pH, **SLC6A2**—a presynaptic amine transporter that inhibits both **DRD2** and **SLC6A4**—and the presynaptic serotonin transporter **SLC6A4** that interacts with many landscape proteins and terminates the action of serotonin in the synaptic cleft by transporting serotonin (back) into presynaptic neurons. In addition, **SLC23A1** transports **vitamin C** into presynaptic neurons, **SCL26A2** transports sulfate into these neurons (not shown), and **SLC30A9** as well as **SLC39A12** transport **zinc** into postsynaptic neurons and astrocytes, respectively.

Several membrane proteins in the landscape also have an important role in regulating cell adhesion, i.e., **CD47**—which is bound and activated by **THBS1** and forms a complex with the (microglial) **ITGA4-ITGB1-complex**—and **CD276** that form a presynaptic complex, and **CD302** that forms a presynaptic complex with **OPRM1**. Furthermore, **CNTN6**—which complexes with **NOTCH1**, leading to the release of **NOTCH1-ID** to the nucleus (not shown)—and **CNTNAP2**—of which the expression is regulated by the transcription factor **FOXP2**—are proteins of the contactin family that regulate (pre)synaptic cell adhesion. In addition, **CDHR1** is a cell adhesion protein of which the expression is upregulated by **CX3CL1**, while presynaptic **NRCAM** and the postsynaptic protocadherins **PCDH7**, **PCDH12** and **PCDH17**—which also interact with each other—are cell adhesion proteins that are involved in the establishment and maintenance of specific neuronal connections in the brain. Lastly, the teneurin proteins **TENM2**—that binds the **ADGRL1** receptor—and **TENM4**—that complexes with extracellular **OLFM1** (see below)—also regulate neuronal cell adhesion and connectivity. 

Lastly, a number of ‘other’ membrane proteins act in the landscape. First, presynaptic neuroligins such as **NLGN3** and **NLGN4X** regulate synapse function and synaptic signal transmission through forming a synapse-spanning functional complex with postsynaptic neurexins such as **NRXN1**. In the same way, presynaptic **EFNA5** and postsynaptic **EPHB2** can form a synapse-spanning complex that modulates synaptic function. Moreover, the membrane protein **RIMBP2** regulates (pre)synaptic transmission through interacting with the membrane-located scaffold protein **RIMS1** and the calcium channel **CACNA1D**. **AGRN** (**agrin**) is a transmembrane protein that is large enough to span the synaptic cleft and act across it [89,90] (not shown) and forms multiple functional complexes with other intra- and extracellular landscape proteins. Another membrane protein that interacts with many other landscape proteins is **LMBR1L**. In addition, the membrane protein **KIDINS220** is a key regulator of synaptic plasticity through binding and interacting with extracellular **FN1** and **OLFM1**, as well as cytoplasmic **GAK** (see below). In turn, **OLFM1**—which also binds **TENM4** (see above)—inhibits complex formation between the inner cell membrane-associated protein **RTN4R** and the transmembrane protein **LINGO1**, with the **RTN4R**-**LINGO1-complex** being a key regulator of axonal growth.

- Cytoskeleton

The cytoskeleton has three components, i.e., actin filaments, intermediate filaments, and microtubules (MTs), and a large number of landscape proteins regulate the function of these components and interact with each other as well as cytoplasmic, nuclear, and membrane-located proteins. In CNS cells, the cytoskeleton is crucial for cell shape and physiology, and it also forms specialized structures such as growth cones—that are responsible for axon elongation and guidance during development—dendritic spines and synapses—that form the structural basis for nerve cell communication and higher order processes such as learning and memory—and membrane specializations critical for the initiation and propagation of nerve impulses.

Firstly, actin filaments play an important role in neuronal development, including regulating growth cone dynamics (**ACTR3**), remodeling of dendritic spines (**ABI2**, **DBN1**), and migration of neuronal precursors [91] (**ABI2**, **DBN1**). In addition, certain landscape proteins link the cytoskeleton to the cell membrane (**ANK3**, **EPB41**), while other—at least to some extent cytoskeletal—proteins regulate actin filament organization (**KLHL5**, **LIMCH1**, **MPRIP**, and **PDLIM7**), actin-based transport (myosins including **MYO10**, **MYO19**), and cell adhesion (**CTNNA3**, **LIMCH1**, **PKP4**, **TLN2**, and **TRIP6**). 

Secondly, intermediate filaments (or neurofilaments) are important for organelle positioning, transport, and function [92], and **PRPH** is an important protein in neurofilaments. 

Lastly, the crosstalk between actin filaments and MTs is important for regulating cytoskeleton-associated processes such as cell migration, cell division, cell polarity and cell (neuronal) shape. More specifically, MTs are composed of tubulin dimers—different combinations of **TUBA1A**, **TUBA1B** and/or **TUBA1C**—and serve as routes for intracellular transport and structural support for dendrites and axons. In addition, MTs contribute to the development, maintenance, and plasticity of synapses, including roles in (pre)synaptic vesicle (re)cycling, mitochondrial arrangement, and interactions with receptors in the neuronal membrane [93]. In addition, the centrosome is the main MT-organizing center and is the main site of microtubule nucleation and anchoring involved in many processes, particularly during cell division, cell migration and differentiation. In this respect, the landscape contains several proteins that are involved in centrosome function: **CEP85L**, **CEP128**, **CENJP**, **HAUS3**, **MPHOSPH9**, **PCM1**, and **WDR62**. Furthermore, the landscape contains a large number of MT-associated motor proteins that move along MTs and regulate intracellular protein trafficking and transports: proteins from the **DNAH-complex** (**DNAH1**, **DNAH3**, **DNAH5**, **DNAH7**, **DNAH10** (not shown), and **DNAH11**), **DYNC2H1** and **DYNC2I1** (involved in retrograde transport), **KIF26B** and **KLC1** (that specifically regulate organelle transport along MTs). 

Lastly, landscape proteins regulate the function/stability/organization of MTs, including **ABGL4**, **CAPN6**, **CCDC66**, **MAPT**, **MTUS2**, **NCKAP5L**, and **NINL**.

- Cytoplasm

The cytoplasm has many functions in (neuronal) cells, including regulating signal transduction between the cell membrane and the nucleus and/or cellular organelles/other cell parts, producing molecules/metabolites involved in many signaling cascades (e.g., glycolysis, gluconeogenesis, protein biosynthesis) and storing or transporting these molecules/metabolites from their production site to other parts of the cell, post-translational modifications of synthesized proteins, and cell cycle regulation. 

First, a number of landscape proteins are located in the cytoplasm but mainly regulate cytoskeletal processes. These proteins include **RHOA** and its activators **ARHGAP26**, **DLC1**, **KALRN**, **NGEF** and **TRIO**. In addition, cytoplasmic **FARP2**, **GCA**, **TJP1** and **TROAP** regulate cell adhesion through interacting with the cytoskeleton. 

Moreover, a large number of cytoplasmic landscape proteins are enzymes, including **DGKQ** (involved in lipid metabolism), **ENO1** (involved in glycolysis) and **POFUT1**—that form a complex, with **POFUT1** also regulating, through fucosylation, membrane-located **NOTCH1** and transmembrane **AGRN**—**MDH1** (involved in the TCA cycle) and **PFKM** (involved in glycolysis and inhibited by **citrate**). Other cytoplasmic landscape enzymes are involved in regulating the metabolism of phosphatidylinositol (PI), with (changes in) PI (metabolites) having been linked to normal human brain development and aging as well as organizing the cell membrane [94,95], i.e., **IMPA1** (important enzyme for maintaining intracellular levels of the PI metabolite **myo-inositol** (**MI**) that mediates brain signaling in response to hormones, neurotransmitters and growth factors [96]), **OSBPL2**, **PI4KA**, **PIKFYVE** and **PLCH1**. Furthermore, two (partially) cytoplasmic landscape enzymes—**AHCY** and **COMT**—are involved in the metabolism of S-adenosylmethionine (SAM)—the methyl donor for most methylation reactions in cells, including histone and DNA methylation in the nucleus—that for its synthesis requires **betaine**, which itself is synthesized in the mitochondria (see below). In addition, **PRMT1**—which is activated by **FAM98B**—is involved in (arginine) methylation of multiple proteins and histones. Moreover, cAMP—that is produced by **ADCY2**—is degraded by **PDE4A**, an enzyme that binds and interacts with **PRKAR2A**, a kinase that is regulated by cAMP. Furthermore, **SULT4A1** is involved in the metabolism of multiple neurotransmitters. Lastly, **HDC**—an enzyme that is upregulated by **TNFB**—interacts with the histamine receptor **HRH2** (see above) and converts **histidine** to histamine using pyridoxal 5′-phosphate (**PLP**, the active form of vitamin B6) as cofactor, while **WWOX** is an oxidoreductase enzyme that interacts with multiple landscape proteins in the cytoplasm and nucleus where it functions as an adaptor protein and transcriptional repressor, respectively. 

Multiple cytoplasmic proteins also regulate (mainly presynaptic) vesicle transport/trafficking (**CLTC1**, **NSF**, **VPS13A**), recycling (**GAK** and **STON2** (highly expressed in astrocytes) and exocytosis (**DYSF**, **PREPL**, and **SNAP29**). **DYSF** is a cell membrane and cytoplasmic protein that uses calcium as a cofactor and regulates the expression of both extracellular **FN1** and cytoplasmic **ACTR3**. 

Furthermore, a large number of cytoplasmic landscape proteins regulate post-translational modifications of proteins, i.e., ubiquitination and SUMOylation. Both these modifications are reversible processes that regulate protein localization and activity. Ubiquitination marks proteins for proteasome-dependent degradation, while sumoylation is not used to tag proteins for degradation but modifies proteins involved in many cellular processes including gene expression, chromatin structure, signal transduction, DNA damage response and cell cycle progression. Molecular chaperone proteins such as **BAG5** and **HSP90AA1**—that has very many interactions in the landscape, such as forming a complex with the adaptor protein **ST13**—play an important role in maintaining a protein’s native folding and function, which protects against the buildup of misfolded proteins [97]. When misfolded proteins interact with chaperones (which cannot be refolded), they can be shuttled for ubiquitin-dependent proteasome degradation. 

As for ubiquitination-related proteins, the landscape contains both ubiquitin-conjugating enzymes—including **UBE2J1** and **BIRC6**, an anti-apoptotic protein that has **CASP8** (which itself is a protease with multiple interactions in the landscape) and **DIABLO** as its ubiquitination targets—and many E3 ubiquitin ligases, i.e., **ASB3** (which degrades **TNFRSF1B**) and **ASB8**, the complex of **COP1**, **COPS9**, **DCAF1** (which degrades **ESR1**) and **RBX1**, **DCAF12**, **FBXL17** (which degrades **PRMT1**), **RNF115** (which degrades **EGFR** and **FLT3**), **RNF4** and **RNF41**. After having been ubiquitinated, proteins are degraded by the 26S proteasome complex, of which the landscape proteins **ADRM1**, **PSMD7**, **PSMD14** are subunits. In addition, **PSME4** is an associated component of the proteasome that promotes ubiquitin-independent degradation and binds with **PSMD14** and **FBXL17**. Ubiquitination is counterbalanced by the action of deubiquitinating enzymes (DUBs) that remove ubiquitin from target proteins, such as **UCHL1** and **USP4**. Lastly, **DESI1**, **PIAS3**, **PIAS4**, **SENP1**, and **SENP6** are proteins that can operate in the cytoplasm and—mainly—the nucleus and that regulate the SUMOylation of proteins.

The landscape also contains several RNA-binding proteins (RBPs) that function both in the cytoplasm and nucleus and regulate various aspects of mRNA metabolism, including mRNA processing, stability, transport and translation and thus affect neurodevelopment, synapse homeostasis, and the neuronal cytoskeleton [98]: **EDC4**, **MARF1**, **MEX3B**, and **PAN3**—that all form a complex—**IGF2BP1**, **NCL**, **PIWIL1**—which binds and stabilizes the mRNA of **DHX57**—**MAPT**, **RBMS1** (that complexes with **MEX3B**), **RBFOX1**, **SPATS2**, **TNRC6A**, and **YTHDF2**. 

In addition, the landscape contains many cytoplasmic kinases that are involved in specific signaling cascades, including **MAPK3**—a kinase that regulates and interacts with many other landscape proteins—and the NF-kappa-B (NFKB) kinase complex that interacts with the landscape proteins/kinases **LRRC14**, **TANK**—that downregulates **TNFB** expression—**TBR1**, **TNIP2**, **TRAF3**, and **TRIP6**. Other kinases in the landscape include **CRKL** (a kinase that interacts with multiple other landscape proteins, including increasing the expression of **HDC** and **TNFB** as well as activating **MAPK3**), **GRK4** (which inhibits **MAPK3**), and the cAMP-dependent **PRKAR2A**. In addition to kinases, a number of phosphatases operate in the landscape, such as **DUSP6**, **PPP1R3A**, **PPP1R3B** and **PPP2R2B**. 

Furthermore, multiple cytoplasmic landscape proteins—some of which can also be located in the nucleus—regulate the cell cycle. In this respect, **CDKN1A**, a protein with many interactions in the landscape, is an important regulator of cell cycle progression (and other landscape processes). **PIM1** phosphorylates **CDKN1A**, which results in the relocation of **CDKN1A** to the cytoplasm and enhanced **CDKN1A** stability, while the nuclear/cytoplasmic protein **FHIT**—that also complexes with **ENO1**—upregulates **CDKN1A** expression. Other cytoplasmic landscape proteins involved in cell cycle regulation are the kinases **GAK**, **PKN2** and **STK38**, and **TOB2**. 

Lastly, four ‘miscellaneous’ landscape proteins can be located in the cytoplasm and—to some extent—in the nucleus and have (very) many interactions with other landscape proteins, i.e., **CASP8**, **EP300**, **ESR1** and **RICTOR**. First, **CASP8** is a key regulator of apoptosis and its activity/expression is regulated by **arachidonic acid**, **butyric acid**, **citrate**, **GABA**, **glutamate** and **palmitic acid**. Furthermore, **CASP8** activity is regulated by—among other proteins—cytoplasmic **DIABLO** and nuclear **IP6K2** (an enzyme involved in phosphatidylinositol metabolism, see above). In addition, **EP300** functions as an acetyltransferase for proteins such as **ESR1**, **ETS1**, **GABPB1**, **PHF5A** and **TADA3** (in the nucleus), and **MAPK3**, **MAPT**, **PRMT1** (in the cytoplasm). Upon binding its ligand, the female sex hormone **estradiol**, **ESR1** can either function as a cytoplasmic adapter protein or as a nuclear transcription factor. The nuclear translocation—and hence transcriptional activation/activity—of **ESR1** activity/activation is regulated by **MACROD1** and the **WWC1**-**DLC1**-**complex**. **RICTOR** is part of the mammalian target of rapamycin (mTOR) complex 2 (mTORC2), a multiprotein complex critical for cell growth and metabolism. **RICTOR** forms a functional complex with multiple landscape proteins and regulates the expression of membrane-located **DRD2**, cytoplasmic **PSMD7** and **PSME4**, and mitochondrial **NDUFA4**.

- Organelles

Multiple interacting landscape proteins are involved in regulating mitochondrial functioning in both pre- and postsynaptic neurons.

Specifically, several TD landscape proteins are mitochondrial matrix proteins that regulate mitochondrial translation, including translation factors (**GUF1**, **LRPPRC**, **GFM2**, **MTIF3**, **MTRFR**), mt-tRNA synthetase (**NARS2**), 39S subunit proteins of mitochondrial ribosome (**MRPL3** and **MRPL40**) and rRNA methyltransferase **MRM1**. Mitochondrial translation is essential for maintaining the cellular energetic balance through the synthesis of proteins involved in the oxidative phosphorylation (OXPHOS). This is required for adenosine triphosphate (ATP) production and the folding of the mitochondrial cristae. Therefore, impaired mitochondrial translation results in diminished ATP production and consequent cellular energy deficit [99], as well as impaired maintenance of mitochondrial DNA (mtDNA) [100].

Several mitochondrial proteins are involved in importing and sorting other proteins (**IMMP2L**, **XPNPEP3**, and **DNAJC15**). Specifically, **IMMP2L** and **DNAJC15**—located in the mitochondrial inner membrane—are involved in the processing and activation of **DIABLO**, which is subsequently released into the cytosol, where it can initiate apoptosis through activating caspases (such as **CASP8**). Other landscape proteins are involved in mitochondrial fusion and cristae formation (**OPA1**) and mitophagy (**PARK7** and **MAP1LC3B**).

Multiple mitochondrial landscape proteins operate in metabolic pathways. These include proteins that regulate the metabolism of: (1) carbohydrates related to the tricarboxylic acid (TCA) cycle, such as the interconversion of **citrate** (**CIT**) to isocitrate via **cis-aconitate** (**CAA**), catalyzed by **ACO2**, the transport of **citrate** by **SLC25A1**, and the conversion of malate to **pyruvate** catalyzed by **ME2**; (2) the urea cycle, in which the **CPS1** enzyme is required to convert ammonia into urea and protect the brain from ammonia toxicity [101]; (3) amino acids, including **NAT8L**, an enzyme that synthesizes *N*-acetylaspartate (NAA), which is subsequently converted with **glutamate** to form *N*-acetylaspartyl-glutamate (**NAAG**), in a cytoplasmic reaction catalyzed by **RIMKLA** and **RIMKLB**; (4) choline and **betaine**, with both enzymes catalyzing one step of the two-step process of choline to **betaine** conversion: **CHDH** and **ALDH7A**; and (5) detoxification/control of reactive oxygen species (ROS) levels and glutathione metabolism (**TXNRD2** and **MGST1**).

The neurodevelopment and normal function of synapses also depend on a readily available supply of ATP. In neurons, the majority of ATP is generated in the mitochondria by OXPHOS via the electron transport chain (ETC) and the ATP synthase complex. Multiple landscape proteins are subunits or assembly factors of the ETC, specifically Complex I proteins (**NDUFA13**, **NDUFA6**, **NDUFB1**, and **NUBPL**), Complex IV proteins (**NDUFA4**, **PNKD**), and the ATP synthase complex (**ATP5IF1**). Furthermore, creatine kinase B (**CKB**) reversibly catalyzes the transfer of phosphate between ATP and creatine (**CR**) for the synthesis of phosphocreatine (**PCR**) in the so-called phosphocreatine shuttle, which acts as an energy-buffering system between the mitochondrial sites of ATP production and the cytosolic sites of ATP utilization. In addition, **MPV17**, an ion channel in the inner mitochondrial membrane, may also be involved in the control of OXPHOS, apart from its role in mitochondrial deoxynucleotide homeostasis and mtDNA maintenance. Similarly, **POLG** that encodes the catalytic subunit of DNA polymerase γ, is responsible for mtDNA replication and maintenance.

Lastly, **MICU3** is a brain-specific enhancer of mitochondrial calcium uptake that forms a heterodimer with **MICU1**.

The endoplasmic reticulum (ER) is a large, dynamic organelle that has multiple roles in the cell. First, the ER has an important role in lipid biosynthesis and metabolism, e.g., enzymes such as **CEPT1** (involved in phospholipid metabolism), **CERS5** and **SGPP2** (involved in sphingolipid metabolism), and **DHDDS** and **NUS1** (involved in lipid metabolism in general). In addition, ER-located enzymes in the landscape are involved in protein modification, including fucosylation (**POFUT1**) and palmitoylation (**ZDHHC11**, which catalyzes the addition of **palmitic acid** onto various proteins thus affecting their localization and function). Furthermore, landscape ER proteins regulate intracellular protein transport: **LMAN2**, **MPPE1**, **PIGW**, **SORT1**, and **VAMP7**. Other landscape proteins are involved in ubiquitin-dependent degradation of misfolded ER proteins: chaperone proteins **DNAJC18** and **DNAJC22**, **GABARAPL2**, **SEL1L**, **SELENOK**, **TMBIM6**, **TMEM33**, and **UBE2J1**. Lastly, **TMBIM6** also functions as a calcium transporter that modulates ER calcium homeostasis. 

The Golgi apparatus (GA) is the main site of protein modification, which includes transferring chondroitin sulfate (CS), the most abundant type of proteoglycan expressed in CNS acting as a barrier molecule affecting axonal growth, neuronal cell migration and plasticity [102] (**CSGALNACT2**), and transferring **palmitic acid** onto **DRD2** (**ZDHHC8**), which is important for **DRD2** relocating to the neuronal membrane [103]. In addition, the GA is involved in regulating protein trafficking (**AP3B2**, **DOP1B**, **MPPE1**, **SORT1**, and **VAMP7**) and **zinc** transport (**SLC30A6**). 

The peroxisomes are multifunctional organelles that contribute to fatty acid/lipid metabolism (**ACAA1** and **SLC27A2**) and metabolite/cofactor transport (**SLC25A17**, which is inhibited by **pyridoxal 5′-phosphate**). In addition, two landscape proteins are involved in peroxisome biogenesis and proliferation, i.e., **PEX2** and **PEX11B**. 

The lysosomes constitute the major proteolytic compartment and contain multiple landscape proteins, i.e., **KLHL22**, **LAPTM5** and **VPS13A**, that are involved in the degradation of target proteins such as **DEPDC5** and **NPRL2**. In addition, the peroxidase **MPO** is activated by **arginine**, inhibited by **butyric acid**, forms a functional complex in the extracellular space with **FN1**, and is involved in oxidative stress and lysosomal damage. 

In presynaptic neurons, several landscape proteins regulate the function of endosomes (EN), including membrane trafficking, degradation of proteins such as **EFGR**, and protein transport to lysosomes or cytoplasmic vesicles (CVs) (**ARL8B**, **DNAJC13**, **PIKFYVE**, and **ZFYVE28**). In turn, CVs mediate autophagy and protein transport to and from the plasma membrane and between organelles, and landscape proteins are specifically involved in CV-linked autophagy (**GABARAPL2**, **MAP1LC3B**, and **TBC1D5**), CV trafficking, exocytosis (of proteins such as **PAM**, an enzyme that catalyzes the conversion of inactive to active (secreted) neuropeptides) and/or recycling (**ASTN2**, **CLTCL1**, **PTPRN2**, **SORT1**, and **VAMP7**).

- Nucleus

In the nucleus of pre- and postsynaptic neurons, four groups of proteins can be discerned. The first group of proteins are part of and/or regulate the function of the nuclear pore complex (NPC) that mediates nucleocytoplasmic transport, genome organization and gene expression [104]: **GLE1**, **NUP85**, **NUTF2**, **RANBP1**, **RANGAP1**, **TOR1A**, **TNIK**, and **WDR62**. The second group of proteins regulate rRNA processing and ribosome synthesis, including **BMS1**, **DGCR8**, **NOP14**, and **TCOF1**. The third group of landscape proteins in the nucleus are functionally involved in transcriptional regulation as well as DNA and histone modifications. In this respect, the landscape contains a large number of transcription factors, such as **BBX**, **CDX2**, **GABPB1**, **GTF2H1**, **GTF2IRD1**, **HOXB4**, **JUND**, **MYT1L** and **POU3F2** (which both have a key role in neuronal differentiation), **NR4A2** (which is highly expressed in dopaminergic neurons and, e.g., upregulates **DRD2** expression), **PHF3**, **TEAD2**, **TERF2IP**, **USF2**, and the zinc finger proteins **ZNF536**, **ZNF664**, **ZNF837**, and **ZNHIT3**. In addition, some of the landscape transcription factors are specifically known to activate gene transcription, e.g., **ETS1**—which is activated by **TCF20**, itself a transcriptional activator—**GTF2A1**, **MRTFA**, and **POU4F2**. Other transcription factors specifically repress gene transcription, including **AEBP1**, **ATN1**, **BHLHE40**, **FOXP1**—which is specific to dopaminergic neurons, regulates the expression of landscape proteins such as **CDKNA1** and **CNTN6**, and is regulated by **EP300**—**POU4F2**, **RERE**—which functionally interacts the intracellular, nuclear domain of **NOTCH1** (**NOTCH1-ID**)—**RUNX1T1**—which interacts with **ATN1**, **ETS1** and the histone-modifying enzyme (see below) **EP300**—and **TBR1**. Furthermore, the landscape contains a few other proteins that regulate the transcriptional process itself, i.e., the subunits of the DNA-dependent RNA polymerases II (**POLR2I**) and III (**POLR3C**, **POLR3H**), and **XRN2**. Moreover, as transcription proceeds, transcripts are (differently) capped, spliced, and polyadenylated so they can be efficiently exported across the nuclear envelope to the cytoplasm for translation of mRNA to protein. Landscape proteins that are involved in this pre-mRNA processing include **FIP1L1** (involved in polyadenylation), **LRPPRC** (which, in addition to its role in the mitochondria (see above), regulates nuclear mRNA export), and multiple proteins that regulate pre-mRNA splicing, as part of splicing complexes: **ESS2**, **GEMIN6**, **SF3A2**, **SNU13**, three splicing factors of the SRSF protein family—**SRSF3** (also involved in nuclear mRNA export), **SRSF4** and **SRSF7**—and **SUGP1**. In addition, **WDR61**—a nuclear protein that has multiple interactions with other landscape proteins—and **PHF5A** bind each other and are a part of the PAF1C complex that regulates transcription elongation and chromatin structure (see below) [105], with **PHF5A** also being involved in pre-mRNA splicing. 

Multiple landscape proteins are implicated in chromatin remodeling, i.e., post-translational modifications (PTMs) of DNA, histones and non-histone targets, including acetylation, methylation and sumoylation, which in turn affects gene transcription [106]. The PTMs are performed by multi-protein complexes that are recruited to act at specific regions of chromatin, and landscape proteins that are members of these complexes include **ACTR8**, **INO80D** and **MCRS1** (that bind and are part of the INO80 complex), **BCL11A** and **BCL7A** (subunits of BAF complex), **BRPF3**, **KANSL1**, **KAT14** and **TADA3** (that bind and are part of the ATAC complex, with **TADA3** also activating **CDKN1A** and being regulated by **EP300**), **MORF4L2**, and **L3MBTL2** and **PHC3** (that are part of the polycomb repressor complex, which keeps genes in a non-transcribed state). As for specific PTMs, the aforementioned and other landscape proteins are involved in DNA/histone acetylation (**BRPF3**, **EP300**—that also acts on non-histone targets, including **ETS1**, **GABPB1** and **PRMT1**—**KANSL1**, **KAT14**, **MCRS1**, **MORF4L2**, **PHF14**, and **TADA3**), methylation (six histone methyltransferases—**ASHL1**, **KMT5A**, **KMT2C**, **KMT2D**, **NSD1** and **NSD3**—and one histone demethylase, **KDM5B**), and (de)sumoylation (**PIAS4**—which mediates the sumoylation of, e.g., **PARK7**—and **SENP6**).

Lastly, the fourth group of nuclear landscape proteins are involved in DNA damage repair: **ERCC5** (involved in repairing UV-induced DNA damage), **GTF2H1** (a transcription factor (see above) that also repairs damaged DNA), **IHO1** (which repairs double-strand DNA breaks), **MAU2** and **NIPBL**—which are part of the cohesin complex that repairs DNA damage—**RPA2** (which binds and stabilizes ssDNA) and **XRCC6**, a protein that repairs DNA damage and has very many interactions with other landscape proteins. 

##### Microglial Cells and Astrocytes

- Microglial cells

Microglial cells play diverse roles in brain development and adult brain function, including the regulation of synaptic plasticity and pruning. They also serve as brain macrophages and are important for the brain immune response, as they are primary sources of immune response factors such as cytokines that in turn can modulate synaptic plasticity [107]. The membrane receptors/proteins (**CD47**, **CX3CR1** (which is a microglia-specific receptor), **ITGA4**, and **ITGB1**) and extracellular cytokines (**CX3CL1**) and other molecules (**FN1** and **TNN**) that are expressed in and regulate the function of microglial cells have already been described above in the part about pre- and postsynaptic neurons.

- Astrocytes

Astrocytes and their projections envelop pre- and postsynaptic neurons and closely approach the synaptic cleft, representing key components of the synapse that are active mediators of synaptic function [108,109]. In addition, astrocytes are important for maintaining the blood–brain barrier (BBB) and brain cholesterol metabolism [110]. A number of protein interactions that (also) occur in astrocytes have already been described above in the part about pre- and postsynaptic neurons. Below, we have added proteins and their interactions that are—to some extent—specific to astrocytes. 

**NR1H2** (also known as Liver X receptor beta, LXRβ) is a transcription factor that is mainly expressed in astrocytes and other glial cells [111] and that has an important role in regulating brain cholesterol metabolism and dopaminergic neuronal function. In the landscape, **NR1H2** downregulates the expression of both **TSHR** and **DIO2**. **TSHR** is a GPCR that signals through stimulatory G-proteins and forms a functional complex with **FN1** but that also regulates thyroid hormone synthesis through binding and being activated by its ligand, thyroid stimulating hormone (**TSH**). In addition, **DIO2** is an ER membrane-located enzyme that the conversion of thyroxine or **T4** (the inactive form of thyroid hormone) to triiodothyronine or **T3** (the active form of thyroid hormone). **T3** generated by **DIO2** is then transported out of astrocytes and into neurons by the membrane transporter **SLC16A2**. Furthermore, **DIO2** is degraded by the ER membrane-located ubiquitination protein **UBE2J1**, it forms a complex with cytoplasmic **RBX1**, and it is involved in downregulating the expression of the transcription factor **NR4A2**. **NR1H2** also regulates the expression of the membrane cholesterol/lipid transporter **ABCG8**. Lastly, histamine (see above) is transported in and out of astrocytes (and presynaptic neurons) by the membrane transporters **SLC22A3** and **SLC29A3**, while in astrocytes, it is also methylated and hence inactivated by the **HNMT** enzyme. 

Below, we will provide six examples of key landscape proteins that are interesting putative (novel) drug targets for TD—i.e., FLT3, NAALAD2, CXC3R1 and CXC3L1, OPRM1, and HRH2—and we discuss why and how these targets can be linked to four aspects of target specificity (i.e., regional, temporal, molecular and modulatory specificity).

## 3. Discussion

In this paper, we have compiled, analyzed and integrated different types of omics data into a molecular landscape of TD. Below, we will discuss the main findings from our analyses and provide examples of putative drug targets derived from the landscape that could be modulated with a beneficial effect on TD. 

First, we tested the (general) hypothesis that the expression of genes for a specific disease will be relatively enhanced in tissue and cell types that are vulnerable to this disease. We found that TD candidate gene expression is enhanced in the brain and pituitary, two tissues that were shown to be rich in neural cells [112]. Our subsequent spatiotemporal analysis of brain tissue revealed that especially the cerebellum, cortex, striatum, and thalamus across various developmental periods may be involved in TD etiology. These results are consistent with previous reports of alterations in anatomical and functional circuits involving the cortex, striatum and thalamus (see above) as well as the cerebellum being key contributors to the pathogenesis of TD [113,114]. Furthermore, our analyses of mouse data showed enriched expression of TD candidate genes in two cell types in particular, i.e., Drd2+ MSNs and layer 6 corticothalamic neurons. In keeping with their potential involvement in TD, these two cell types are also enriched among genes that were found to be downregulated in the postmortem striatum of TD patients, which further suggests that TD onset and progression may be particularly related to deficits in the function or presence of these cells. Drd2+ medium spiny neurons (MSNs) are inhibitory/GABAergic neurons (MSNs) that express the dopamine D2 receptor and, together with Drd1+ MSNs, they constitute approximately 95% of the neurons in the striatum, the main input structure of the basal ganglia. Drd2+ MSN neurons act as crucial regulators of striatal output via the ‘indirect’ pathway and so alter striatal-mediated ‘action’. Furthermore, these MSNs receive convergent excitatory inputs from the cortical and thalamic areas and further project to the output nuclei of the basal ganglia circuit [115]. In addition, Drd2+ MSNs have been shown to be crucial in habit formation in mice [116,117]. This is particularly interesting because both from a cognitive-behavioral and neuroscientific perspective, tics—the hallmark of TD—can be viewed as habits that have been formed over time and that are associated with premonitory sensations and can, at least partially, be controlled [118,119,120,121,122]. As a result, habit reversal training (HRT) is currently among the first-line behavioral interventions aimed at reducing tics [123,124]. Furthermore, rodent studies have shown that Drd2 expression changes during development, with increased expression across early postnatal life and peak Drd2 expression by early adolescence, followed by decreased expression in adulthood (reviewed in [125]). In accordance, the dopaminergic excitability of Drd2+ MSNs also decreases during the juvenile period [126] and in humans, a similar peak of dopaminergic innervation of the striatum was observed in preadolescence, with a subsequent decrease during adulthood [127]. In addition, postmortem studies have shown an increased DRD2 density in the frontal cortex and striatum of TD patients [128,129] while it was also demonstrated in monozygotic twins that differences in DRD2 binding influence TD severity [130]. Based on all these findings, it seems that drugs aimed at reducing DRD2 activity during the critical time window associated with TD (i.e., childhood to (early) adolescence) would be beneficial and, indeed, DRD2 antagonists are currently still the standard pharmacological treatment of TD/tics [131]. The downregulated genes in postmortem TD striatum were also enriched for genes that are highly specific to cholinergic neurons, in line with the observed reduction in cholinergic (ChAT+) interneurons in postmortem immunohistochemical studies of TD patient striatum [64,132]. Interestingly, habit formation (see above) is (also) modulated by striatal cholinergic interneurons [133,134], and these neurons are also important for synchronizing the activity of (Drd2+) MSNs that suppresses or ends a movement bout [135]. In addition, pharmacotherapy with cholinergic drugs has been observed to modulate motor tics [136]. Lastly, we found that the upregulated genes in postmortem TD striatum are highly expressed in glial and immune cells, which may imply that inflammation-mediated mechanisms (also) contribute to TD, but this enrichment was not found through our analysis of the genetic data. Taken together, our tissue and cell type specificity analyses suggest that TD is not confined to a single brain region and that there are potentially multiple cellular routes to TD. 

Our analyses of the TD candidate genes revealed two significantly enriched pathways: ‘cAMP-mediated signaling’ and ‘Endocannabinoid Neuronal Synapse Pathway’. In Figures S1 and S2, we provide a graphical representation of the two pathways at the cellular level in which the proteins encoded by TD candidate genes and the metabolites implicated through the PRS-based analyses are indicated in purple. First, cAMP (3′-5′-cyclic adenosine monophosphate) is an important second messenger molecule that is used for intracellular signal transduction. cAMP is produced from ATP by adenylate cyclases (such as the landscape protein ADCY2) that themselves are activated through stimulatory GPCRs (including the landscape proteins CRHR1, HRH2, OPRM1, and TSHR) or inhibited through inhibitory GPCRs (landscape proteins DRD2—the dopamine receptor that interacts with many landscape proteins and is also enriched in TD-linked Drd2+ neurons (see above)—FPR1, FPR2, FFAR3, OPRD1, OPRK1, and OPRM1). Furthermore, cAMP is degraded by phosphodiesterase enzymes [137] such as the landscape protein PDE4A and it regulates synaptic function through activating protein kinase A (PKA) (of which the landscape protein PRKAR2A is a regulatory subunit) [138]. All these findings suggest that abnormalities in cAMP signaling could be a central functional theme in TD etiology. In this respect, studies of postmortem brains from TD patients have revealed reduced concentrations of cAMP in the cerebral cortex and putamen [139,140]. Conversely, increased cortical and striatal levels of cAMP and associated reduced levels of phosphodiesterase activity have been associated with stereotypic, tic-like behavior of deer mice [141]. Changes in cAMP levels and activity in dopaminergic neurons during development may also underlie tic-like symptoms in ADHD, a disorder that is highly comorbid with TD [142]. Lastly, and interestingly, some drugs that target cAMP-mediated signaling are already in use to treat TD or in the clinical trial phase, e.g., the DRD2 modulator aripiprazole, the DRD2 antagonist risperidone and the opioid receptor antagonist naloxone (see below), further implying that cAMP signaling is altered in TD. 

The second pathway that is enriched within the TD candidate genes points towards an involvement of (altered) endocannabinoid signaling in TD etiology. The endocannabinoid system (ECS) comprises two cannabinoid receptors—CNR1 and CNR2—their ligands, the endocannabinoids, and the enzymes regulating endocannabinoid synthesis and degradation. CNR1 is highly expressed in the CNS, while CNR2 is mainly expressed in immune cells and activated microglia, with some expression also detected in the CNS. Furthermore, endocannabinoids are endogenous lipid-signaling molecules that are produced in the cell membrane from phospholipid precursors and act as messengers that modulate pre- and postsynaptic functions [143,144] in multiple brain regions. The two best characterized endocannabinoids are arachidonoyl ethanolamide (AEA or anandamide) and 2-arachidonoylglycerol (2-AG). Interestingly, endocannabinoids are also derivatives of arachidonic acid (AA) and our PRS-based analyses implicated genetic sharing between TD and increased AA blood levels (see below). Previous studies have also suggested altered ECS signaling in the pathophysiology of TD. In this respect, studies have yielded inconclusive results regarding genetic variation in *CNR1* being associated with TD [145,146] but significantly increased CSF levels of several endocannabinoids as well as AA were reported in TD patients compared with controls [147]. In addition, the enriched ‘Endocannabinoid Neuronal Synapse Pathway’ contains the key landscape protein DAGLA, an enzyme that can be located in the pre- and postsynaptic membrane (see above) and that produces 2-AG in an autocrine fashion [148]. Moreover, *Dagla* KO zebrafish show stereotypical movements and deficits in motion perception [149]. Interestingly, several enzymes in the enriched endocannabinoid-related pathway (shown in Figure S2) are also encoded by genes that are not TD candidate genes—and that are therefore not in the landscape—but of which the expression is differentially regulated in the blood of TD patients. Specifically, FAAH—an enzyme that hydrolyzes AEA to AA—was upregulated in the blood of TD patients aged 5–9 [66]. In addition, ABHD6—an enzyme that hydrolyzes 2-AG to AA—was upregulated in the blood of TD patients aged 13–16, and ABHD6 expression was positively correlated with symptom severity in adult TD patients [66,67]. AEA can also be produced through the hydrolysis of *N*-acyl-phosphatidylethanolamines (NAPEs) by the enzyme NAPEPLD—that was downregulated in the blood of TD patients aged 5–9 [66]—or by the combined action of the enzymes ABHD4 (downregulated in the blood of TD patients aged 13–16) and GDE1 (of which the expression in blood was negatively correlated with TD severity) [66,67]. Furthermore, inhibition of FAAH and MGLL (an enzyme that degrades 2-AG to AA) resulted in increased levels of AEA and 2-AG, respectively, which—in mice—disrupted habit formation [150,151] that has been hypothesized to also be underlying TD (see above). In addition, when bound and activated by endocannabinoids, CNR1 can act as an inhibitory GPCR that suppresses cAMP production and subsequent PKA activation [152,153]. This constitutes a link between the endocannabinoid pathway and the cAMP pathway discussed above, with the landscape proteins ADCY2 and PRKAR2A also being involved in both enriched pathways. Moreover, when CNR1 and DRD2 are co-expressed, co-stimulation with agonists of these two receptors led to an increase in cAMP production in striatal neurons, while when applied separately, CNR1 or DRD2 agonists inhibited cAMP production [154], which suggests a link between endocannabinoid, cAMP and dopaminergic signaling. Lastly, cannabinoid receptors are also a prime target of the exogenous cannabinoid D9-tetrahydrocannabinol (THC), the psychotropic component of cannabis. In this respect, a putatively beneficial role of cannabis in TD treatment comes from anecdotal evidence of patients reporting improvement in their tics after using cannabis [155] as well as some case studies and clinical trials (summarized in [156,157]). That being said, as most of these studies provide only low level of evidence for a beneficial effect, the European and American authorities currently only recommend cannabis use for (adult) treatment-resistant TD cases in which established therapy did not alleviate symptoms [10,11,12]. In addition, developmental observations suggest that endocannabinoid receptor expression increases only gradually in the postnatal period, which (partially) explains the observed insensitivity to the psychoactive effects of cannabis in young people. Therefore, it was hypothesized that children may respond positively to medicinal applications of cannabis without undesirable central effects [158]. However, only three single case reports are currently available to suggest that a medicinal form of cannabis would be effective and safe for treating severe tics in minors with TD [159,160,161]. 

In addition to the pathway analysis, we conducted further analyses for biological functions that are enriched within the TD candidate genes. Most of the enriched functions are related to processes such as development, migration and proliferation of neurons/brain cells (leading to neuronal circuitry development), synaptic function, and neurotransmission. In this respect, previous studies have reported altered synaptic plasticity in TD patients compared to controls (in the cortex and brain stem) [162,163,164]. In addition, a recent study assessed the enrichment of multiple gene sets using individual-level genotype data and identified three genome-wide significant gene sets that are implicated in TD, i.e., ligand-gated ion channel signaling, lymphocytic signaling, and cell adhesion and trans-synaptic signaling [5].

Apart from the analyses of which we discussed the results above, we performed PRS-based analyses to determine the presence, extent, and direction of genetic overlap between TD and blood or CSF metabolite levels. Below, we will discuss the main findings from these analyses. As a general comment, we would like to point out that although different levels of metabolites in the blood (plasma or serum) may not directly reflect changes in the brain, they reflect alterations in metabolic pathways in the body and may therefore be associated indirectly with the development of TD. In addition, many metabolites can cross the BBB—via transporters or diffusion—and changes in the blood levels of these metabolites likely lead to more direct changes in the brain and vice versa. 

First, we found genetic sharing between TD and higher blood levels of betaine. Betaine (also known as trimetylglycine) is an amino acid that is taken up into the body through the diet or can be synthesized in the mitochondria from choline in a two-step process catalysed by landscape proteins CHDH and ALDH7A. Betaine acts as an important cellular osmolyte and a methyl donor for the conversion of homocysteine to methionine—and hence increases methionine levels [165]—as part of the methionine cycle [166]. This cycle produces S-adenosylmethionine (SAM) and S-adenosylhomocysteine (SAH), key modulators of cellular methylation and hence epigenetic regulators [167,168]. Interestingly, MAT2A and MAT2B, two enzymes from the methionine cycle—that converts methionine to SAM—were found to be upregulated in the blood of TD patients aged 13–16 [66], while the landscape proteins PRMT1 and AHCY catalyze the subsequent conversion of SAM to SAH and SAH to homocysteine, respectively. In addition, the blood expression of PRMT1 is correlated with TD severity [67]. Furthermore, methionine was shown to induce stereotypy and prepulse inhibition deficits in mice [169] and to increase amphetamine-induced stereotyped behaviors in rats [170]. Higher homocysteine serum and plasma concentrations were also found in patients with primary dystonia compared to controls [171,172]. All these findings provide further support to an involvement of homocysteine/methionine metabolism in TD, in which alterations in homocysteine/methionine levels would lead to changes in methylation of downstream substrates, including histones and result in altered gene expression [167,168].

We also identified a shared genetic etiology between TD and decreased blood levels of pyridoxate, the primary catabolic product of vitamin B6. Blood levels of pyridoxate are strongly correlated with blood levels of its precursor pyridoxal 5′-phosphate (PLP), the active form of vitamin B6. Therefore, pyridoxate has been suggested as a possible complementary and short-term marker of vitamin B6 status [173]. In this respect, previous studies have shown that supplementation of vitamin B6 in combination with other molecules such as magnesium [174,175] was safe and effective in alleviating symptoms of TD in children and adolescents. Furthermore, enzymes involved in the metabolism of pyridoxate and PLP were found to be differentially expressed in TD patients: ALPL—that catalyzes the dephosphorylation of PLP to pyridoxal (the transportable form of vitamin B6)—was upregulated in the postmortem striatum of TD patients [64], while PDXK—that catalyzes the conversion of vitamin B6 precursors to their phosphorylated counterparts, including PLP—and PHOSPHO2—that dephosphorylates PLP—were downregulated in the blood of children with TD [66]. Moreover, the blood expression of AOX1—that converts PLP to pyridoxate—was positively correlated with TD severity [67]. Interestingly, magnesium—that is a cofactor of many landscape proteins—is (also) a cofactor for most of these enzymes. Lastly, and importantly, vitamin B6 (PLP) acts as a cofactor in various enzymatic reactions, including the conversion of histidine to histamine by the important landscape protein HDC.

Furthermore, we found genetic sharing between TD and increased blood levels of Tumor necrosis factor-beta (TNFB), a cytokine that is produced by lymphocytes. In the landscape, TNFB interacts with several proteins, and it induces downstream signaling by binding to heterodimeric TNFRSF1A and TNFRSF1B [176]. TNFRSF1A and TNFRSF1B expression is upregulated in the striatum of TD patients [64] and the blood expression of TNFSRF1B is negatively correlated with TD symptom severity [67]. In addition, a mutation in *TNFRSF1A* has been linked to persistent tics [177]. A direct involvement of TNFB in the pathogenesis of TD has not been studied but some evidence for its (putative) role in TD comes from studies on auto-immune disorders. First, TNFB regulates the formation of tertiary lymphoid-like structures [178,179], such as the murine nasal-associated lymphoid tissue (NALT) [180] that is analogous to the human tonsils/adenoids [181]. Group A streptococcal (GAS) bacteria that are present in both mouse NALT and human tonsils [182] are crucial in the pathophysiology of PANDAS, an auto-immune disease that presents itself as a combination of tics and OCD-like symptoms [183]. In addition, streptococcal superantigens—that are involved in PANDAS [184]—directly stimulate the secretion of TNFB [185]. Furthermore, TNFB is crucial for protecting against Toxoplasma bacterial infection in the CNS [186], which is interesting because a possible role of Toxoplasma infection in the pathogenesis of TD and tic disorder has been reported [187,188]. 

Furthermore, we identified genetic overlap between TD and decreased blood levels of myo-inositol (MI). MI is a metabolite of the second messenger phosphatidylinositol (PI) [189], and PI and its metabolites regulate normal human brain development and aging as well as the organization of the cell membrane [94,95]. In addition, MI (derivatives) play crucial roles in various processes, such as signal transduction, osmoregulation, membrane biogenesis and trafficking, cytoskeletal organization, gene expression, DNA repair, energy metabolism and autophagy, and they have been implicated in multiple disorders [190]. In line with our findings, a brain MRS study in TD reported reduced MI levels in the left frontal cortex [191]. The levels of intracellular MI are dependent on de novo synthesis, conversion of MI derivatives, uptake from the extracellular fluid and/or degradation. In this respect, a role for altered MI signaling in TD is also implicated by the fact that some key enzymes involved in the synthesis of MI were found to be differentially expressed in the brain and/or blood of TD patients: HK2 (increased in the postmortem striatum of TD patients [64], ISYNA1 (an enzyme that catalyzes the rate-limiting step in MI synthesis and of which the expression was downregulated in the blood of TD patients aged 5–9 and 13–16 [66]) and the landscape protein IMPA1 (upregulated in the blood of TD patients aged 10–12 [66]). Moreover, *Impa1* KO mice showed TD-like behaviors, including increased motor activity in the open field and forced-swim tests, hyperactivity, and stereotypy in the home cage [192]. Four landscape proteins involved in the conversion of MI derivatives were also differentially expressed: PLCH1 (downregulated in TD striatum [64]), PI4KA (downregulated in the blood of TD patients aged 5–9 [66]), PIKFYVE (upregulated in blood of TD patients aged 13–16 [66]) and IP6K2 (alternatively spliced in the blood of TD patients and blood expression is negatively correlated with TD severity [67,68]. Furthermore, the expression of the MI transporter SLC5A11 was dysregulated in the blood of TD patients (upregulated and downregulated in TD patients aged 5–9 and 13–16, respectively [66]). Lastly, MI is catabolized in the kidneys by the enzyme MIOX, and blood expression of MIOX was found to be upregulated in TD patients aged 5–9 [66].

Our PRS-based analyses also revealed genetic sharing between TD and increased or decreased blood levels of multiple types of lipids, including glycerophospholipids, sphingolipids, triacylglycerols, fatty acids, myo-inositol (see above) and lipid ratios. As for fatty acids (FA), these are utilized as energy source, signaling molecules and structural components of membranes [193], and depending on their chemical structure and chain length, they are classified as saturated, monounsaturated, or polyunsaturated (PUFA). In this respect, we found genetic sharing between TD and increased blood levels of saturated, long chain FA such as stearate (stearic acid) and palmitate (palmitic acid), as well as (long chain) PUFA. These PUFA are divided into omega-6 FA—including the essential PUFA linoleate (linoleic acid, LA) that is a precursor of gamma-linolenate (gamma-linolenic acid) and arachidonate (arachidonic acid, AA, see above)—and omega-3 FA, such as the essential PUFA alpha-linolenate (alpha-linolenic acid). Palmitic acid (PA) is the most common saturated FA found in the human body and can be provided through the diet or be synthesized endogenously from other FA—such as palmitoylethanolamide (PAE) that is increased in the CSF of TD patients [147] and that is converted to PA by FAAH, an enzyme that is also involved in AA synthesis (see above)—carbohydrates and amino acids. PA represents 20–30% of total FA in membrane phospholipids and adipose triacylglycerols [194] and it has multiple functions—reflected by the multiple landscape proteins of which it regulates the expression or activity—including palmitoylation, a post-translational modification of proteins that involves the attachment of PA to specific cysteines, which increases the hydrophobicity of cytoplasmic proteins and hence increases their affinity for cytosolic membrane surfaces. Furthermore, AA is the biologically active omega-6 FA and represents about 20% of the neuronal FA. AA is converted to various eicosanoids that are important mediators of inflammation—with both pro- and anti-inflammatory activities—and is involved in regulating synaptic transmission [195]. More specifically, AA is released from membrane phospholipids through phospholipase enzymes. Subsequently, AA can be metabolized by three different groups of enzymes, i.e., cyclooxygenases, lipoxygenases and cytochrome P450 enzymes that generate numerous biologically active mediators, many of which are potential preventive and therapeutic targets for various diseases [196]. In this respect, it is interesting that the cyclooxygenase PTGS1 and the lipoxygenases ALOX5, ALOX5AP and ALOX15B were all found to be upregulated in the postmortem striatum of TD patients [64]. Moreover, and as already discussed above, AA is a precursor for endocannabinoids and therefore, it has an important role in regulating both endocannabinoid and cAMP signaling, the two pathways that were significantly enriched in the TD candidate genes (see above). Linked to this, the extracellular and anti-inflammatory [197] metabolite LXA4—which is synthesized from AA through sequential actions of lipoxygenases—mostly exerts its effects through GPCRs such as the landscape protein FPR2 and CNR1. As for its effect on CNR1, LXA4 was found to act as an allosteric modulator of CNR1, thereby enhancing its affinity for AEA and ultimately decreasing cAMP production [198], which may make LXA4 a potential TD treatment that could be used instead of the cannabinoids themselves (see above). Lastly, omega-3 and omega-6 FA compete with each other in their effects on downstream signaling [199]. In keeping with this, omega-3 FA can decrease the bodily levels of omega-6 FA through being ingested via the diet (e.g., from fish oil) and they have important anti-inflammatory properties, e.g., through inhibiting NFKB signaling [200]. Therefore, it follows that omega-3 FA would constitute a putative adjunctive treatment of TD and indeed, a randomized, double-blind, placebo-controlled trial in children indicated that omega-3 FA supplementation may be beneficial in the reduction in tic-related impairment for some children and adolescents with TD, but not for tics per se [201].

In addition to blood metabolites, we found genetic sharing between TD and the CSF levels of two metabolites: NAAG and butyrate. As for NAAG, we will discuss this metabolite and its links with TD in detail below. Butyrate (or butyric acid, BA) is a short chain fatty acid naturally produced by bacterial fermentation of undigested carbohydrates, such as dietary fiber in the gut. Interestingly, different levels of BA-producing bacterial groups [202,203,204]—including Roseburia [72], Faecalibacteriumin [205] and Bacteroidia [206]—were found in the microbiome of TD/tic disorder patients compared to controls, suggesting that rebalancing of gut microbiota could be a promising biological therapy for TD [207]. BA then travels from the gut through the systemic circulation and reaches the brain, where it crosses the BBB via monocarboxylated transporters of the SCL16 family [208], including SLC16A3, SLC16A4 and SLC16A7 that were found to be differentially expressed in the blood or postmortem brain tissue of TD patients [64,66]. In the brain, BA can act as a regulator of the immune response through its anti-inflammatory actions in microglia [209], an epigenetic regulator that increases gene expression through inhibiting histone deacetylation [210,211] and/or as an endogenous ligand for a subset of GPCRs, including the landscape protein FFAR3 and—not shown in the landscape—FFAR2 (upregulated in postmortem TD brain) [64] and HCAR2 (of which the blood expression is negatively correlated with TD severity) [67]. BA also modulates the expression/activity of many (other) landscape proteins. In the extracellular matrix, BA decreases the expression of COL4A2, COL5A1, COL6A3, IL17A, and TNFAIP2; it increases the expression of ANXA1 and WNT5; and it regulates the acetylation and methylation of RELN. In the cytoplasm, BA increases the activity of HDC—leading to increased histamine synthesis (see below)—and CASP8, while it also increases the expression of CDKN1A and GAK. Furthermore, BA decreases the expression of cytoplasmic ESR1, NCL and PRKAR2A, and it increases the release of DIABLO from mitochondria. In addition, BA activates cAMP-PKA signaling—although independently from GCPR-mediated signaling) [212]—and there is a positive association between BA and endocannabinoids in human subjects (enrolled in a 6-week exercise intervention) [213], indicating that BA regulates both pathways that were enriched in our data. Lastly, previous studies have suggested a beneficial role of BA in treatment of neuropsychiatric conditions, such as ASDs [214,215], Huntington’s disease (HD) and PD, where it exerts neuroprotective effects, supports mitochondrial function and decreases behavioral abnormalities [216,217,218,219] through inhibiting histone deacetylation [218,220]. Furthermore, BA was shown to positively affect memory-related synaptic plasticity [221] and omega 3 FA (see above) were shown to restore the normal levels of BA-producing bacteria [222]. All these findings suggest that approaches aimed at increasing (CNS) butyrate levels—e.g., through changing the gut microbiome or having an omega 3 FA-rich diet—may be considered as (adjunctive) treatments for TD.

Based on all our data and analyses, we built an integrated molecular landscape of TD that contains interactions between more than 500 proteins, metabolites and other molecules, and above, we have already described the main landscape processes. Before providing more details about specific putative drug targets that we identified in the landscape and as a more general comment, we would like to note that multiple landscape proteins—spanning different subcellular locations—are involved in protein degradation. These include (proteins constituting) the ubiquitin-proteasome system in the cytoplasm, ER proteins involved in ubiquitin-dependent degradation of misfolded ER proteins, CV-mediated autophagy, and molecular chaperones, and all these proteins regulate the removal and recycling of misfolded proteins and damaged organelles. Furthermore, impairment of these processes and accumulation of protein aggregates and faulty organelles can lead to the generation of oxidative stress, inflammation, and cell death [223], which in turn affects synapse formation, maturation, and plasticity [224]. In addition, based on the four aspects of target specificity described in the Materials and Methods, we identified a number of putative drug targets in the TD landscape, and we will describe six of these targets in more detail below.

First, FLT3 is a membrane-located receptor tyrosine kinase that regulates inflammation and other immunity-related functions [225]. *FLT3* is the most significantly associated gene in the largest GWAS of TD published to date [55]. FLT3 shows regional specificity for TD, as it is highly expressed in the brain and in our TWAS, we found TD-associated eQTLs with a positive effect on *FLT3* expression in multiple brain regions (top finding for the cortex; Z-score = 4.66 and *p*-value = 3.24 × 10^−6^). In keeping with this, a recent study also found the most significant TD-associated TWAS signal for the cortex and, more specifically, the dorsolateral prefrontal cortex [226]. In the same study, the authors reported an increased expression of FLT3 in lymphoblastoid cell lines derived from TD patients compared to controls [226]. As for its temporal specificity for TD, FLT3 is highly expressed in two brain regions for which we have found spatiotemporal enrichment of TD candidate gene expression (see above), i.e., in the cerebellum (in the neonatal period and infancy, early and middle-late childhood, adolescence, and young adulthood) and in the thalamus (in adolescence and young adulthood). Moreover, FLT3 has considerable molecular specificity for TD as it interacts with multiple other landscape proteins. For example, upon binding its ligand, the cytokine FLT3LG—which has been linked to TD as well, as FLT3LG blood expression is positively correlated with TD symptom severity [67]—regulates the phosphorylation of MAPK3 and MAPT, two highly interactive landscape proteins, while it also regulates the expression of cytoplasmic PIM1 and nuclear EXCC6, two proteins that (also) interact with many other landscape proteins. Lastly, FLT3 (putatively) has modulatory specificity for TD because, and as mentioned above, TD-associated eQTLs upregulate *FLT3* expression in multiple brain regions, which suggests that inhibition of FLT3 (function) could have a beneficial effect on TD symptoms. In this respect, it is interesting that inhibitors of FLT3 have been approved for various cancers and have been trialed with positive effect for multiple T-cell-mediated auto-immune diseases [225] that are genetically and/or clinically overlapping/comorbid with TD [227,228,229]. Moreover, FLT3 inhibitors have been found to have a therapeutic effect in (human) cell and mouse models of Rett syndrome, a genetically determined neurodevelopmental disorder [230], and to alleviate peripheral neuropathic pain in mice [231]. The above being said, additional in silico, in vitro and in vivo studies are needed to further determine if and how FLT3-based treatments for TD could be developed.

Another promising drug target from the landscape is NAALAD2, an enzyme that is expressed in neuronal and astrocytic membranes and that converts *N*-acetyl-aspartyl-glutamate (NAAG) to *N*-acetylaspartate (NAA, which is synthesized by the mitochondrial landscape enzyme NAT8L) and glutamate. Conversely, NAAG is formed from NAA and glutamate by the cytoplasmic landscape enzymes RIMKLA and RIMKLB. As for its regional and temporal specificity for TD, NAALAD2 is highly expressed in the pituitary, neurons and astrocytes [232] and it is highly expressed in the striatum during young adulthood [233], respectively. Furthermore, NAALAD2 has molecular specificity for TD because it works on two important, TD-linked neurotransmitters: NAAG and glutamate. NAAG is one of the only CSF markers for which we found genetic sharing with TD and it is thought to function as a neurotransmitter in both the CNS and peripheral nervous system, with its lowest expression being in the pituitary [234], a finding that is in line with NAALAD2 expression being the highest in this brain region (see above). In a magnetic resonance spectroscopy (MRS) study, patients with TD also had reduced levels of NAA in the left putamen and bilateral frontal cortex [191]. In addition, the main excitatory neurotransmitter glutamate—that is converted from NAAG by NAALAD2—interacts with multiple landscape proteins. Furthermore, a number of MRS studies have investigated the involvement of (brain) glutamate in TD, but this has yielded inconsistent results [235,236,237]. Lastly, it seems that NAALAD2 also has (putative) modulatory specificity for TD. In addition to genetic sharing (see above), we found a negative genetic concordance between TD and CSF levels of NAAG, indicating that genetic variants associated with TD are also associated with decreased NAAG CSF levels. In turn, this suggests that (brain) NAALAD2—which uses NAAG to ‘produce’ glutamate—should be inhibited to treat TD. Furthermore, transcriptomic profiling has shown decreased RIMKLB levels and increased NAT8L levels in the blood of TD patients aged 5 to 9 [66], as well as alternative splicing of *RIMKLA* in the blood of adult TD patients [68]. All these findings suggest that in addition to inhibiting NAALAD2, a treatment to reduce TD symptoms may be to activate/increase NAAG synthesis. However, there are currently no known treatments that increase NAAG synthesis. Interestingly, inhibiting FLT3 (see above) also prevents glutamate-induced toxicity [238]—that could be the result of increased NAALAD2 expression/activity—which constitutes a functional link between NAALAD2 and FLT3. Moreover, NAALAD2 inhibitors—that elevate synaptic NAAG levels [239]—were found to reduce stereotypical movements in different mouse models (of schizophrenia) [240,241], which further highlights the suitability of NAALAD2 as a novel TD target and the need to conduct further experiments to develop it into an effective TD treatment.

Two other interacting putative drug targets from the landscape are the membrane receptor CX3CR1 and its ligand, the chemokine CX3CL1. CX3CR1 is highly expressed in the microglial cell membrane. In addition, the CX3CR1-CX3CL1-complex plays a key role in regulating brain inflammation [242] as well as synaptic pruning and connectivity [243,244,245], while CX3CL1 is also located in the neuronal cell membrane where it can be cleaved into a soluble chemokine by the membrane-located landscape enzyme ADAM10 [246,247]. As for their regional specificity for TD, the expression of CX3CR1 was found to be upregulated in the (postmortem) striatum of TD patients (FC = 1.78) [64] and increased CX3CL1 blood expression is correlated with increased TD symptom severity [67]. Both proteins also show temporal specificity for TD. CX3CR1 expression is upregulated in the blood of TD patients aged 10–12 years (FC = 1.18) [66] and in mouse striatum, Cx3cr1 shows a temporal expression pattern corresponding to developmental stages that could be linked to TD occurrence and resolution (i.e., first upregulation, then downregulation) [248]. In addition, CX3CL1 is highly expressed in the striatum (in the neonatal period, early infancy and early childhood), cortex (neonatal period, early infancy, early childhood and adolescence) and the thalamus (neonatal period and early infancy), while mouse Cxc3l1 also shows the same temporal expression pattern in the striatum than Cx3cr1 does [248]. Moreover, CX3CR1 and CX3CL1 have molecular specificity for TD, and this not only through forming a functional complex with each other but also through additional effects of CXC3L1 on multiple other landscape proteins—that were not all drawn in the landscape but can be found in Table S5—e.g., as a ligand of the ITGA4-ITGB1-complex and through upregulating the expression of extracellular POSTN and membrane-located CDHR1. Lastly, there is some evidence that the CX3CR1-CX3CL1-complex could be modulated with a beneficial effect on TD. However, both an inhibition and activation of this complex have been found to have a neuroprotective effect, depending on whether the intervention was carried out in the developing or adult brain [249]. For instance, neutralizing antibodies against (brain) CX3CR1 ameliorated exogenous CX3CL1-induced PD-like behaviors in an adult rat model [250], while another study in a mouse model of PD revealed the neuroprotective capacity of CX3CL1 that resides solely upon the soluble form but not the membrane-located form of CX3CL1 [251]. Moreover, in a mouse model of Rett syndrome, it was shown that the presence of CX3CR1 is detrimental to the neurodevelopmental trajectory and (partial) ablation of *CX3CR1* attenuated disease severity [252]. For these reasons, further studies are needed to further determine if and how CX3CR1/CX3CL1-based treatments for TD could be developed.

Furthermore, the TD landscape contains three presynaptic membrane-located opioid receptors—OPRM1, OPRK1 and OPRD1—of which we think that especially OPRM1 fits all aspects of drug target specificity for TD. OPRM1 is an opioid receptor of the μ family that mediates downstream signaling through binding both endogenous opioids (such as endorphin and endomorphin) and synthetic opioids (such as morphine, heroin, fentanyl and methadone) [253,254]. OPRM1 shows regional specificity for TD, as OPRM1 is highly expressed in the brain but its expression is specifically downregulated in the (postmortem) striatum of TD patients (FC = −1.43) [64]. OPRM1 also has temporal specificity for TD as it is highly expressed in the thalamus (in the early fetal period, early mid-fetal period, neonatal–early infancy period, adolescence and young adulthood) and the cerebellum (late fetal period and late infancy). Moreover, OPRM1 has considerable molecular specificity for TD as it interacts with multiple other landscape proteins, including the opioid receptors of the δ and κ families OPRD1 and OPRK1. In addition, OPRM1, OPRD1 and OPRK1 are involved in cAMP-mediated signaling (one of the two enriched pathways within the TD candidate genes), while OPRM1 is also a (putative) upstream regulator of multiple landscape genes (see above). As for the—putative—modulatory specificity of OPRM1, it should first be noted that abnormalities of the opioid system have been found in TD previously [255,256]. In this respect, previous reports have shown that pharmacological manipulation of the endogenous opioid system has a beneficial effect on TD (symptoms). Specifically, several case reports [257,258,259] and a randomized, double-blind, placebo-controlled study [260] have suggested that TD symptom reduction may be achieved with an opioid receptor antagonist such as naloxone or naltrexone that both have a high binding affinity for OPRM1 [261]. In addition, some studies have indicated dose-dependent effects of naloxone in patients with TD, with low doses causing a decrease in tics while higher doses cause increased tics [262,263]. Conversely, case reports have also shown that the full OPRM1 agonist methadone and the partial OPRM1 agonist buprenorphine are successful in alleviating symptoms of TD [264,265]. Furthermore, impaired OPRM1 function has been suggested to lead to decreased release of gonadotropin-releasing hormone (GnRH) [266] that is produced by the PROK2-PROKR2 complex in the landscape. In turn, this leads to a reduced secretion of the gonadotrophins LH and FSH that have been found to be especially lower in the plasma of male TD patients, and this associated with the onset of puberty [267]. Given the above, further studies are required to assess which opioid receptor agonists and/or antagonists could be used to treat TD and when these drugs would need to be administered to have the best effect and least side effects. 

The last putative drug target from the landscape that we would like to discuss in some detail is HRH2, a (neuronal) membrane receptor of histamine. Firstly, HRH2 shows regional specificity for TD, as it is highly expressed in both excitatory and inhibitory neurons [268], and its expression in blood has been found to be negatively correlated with symptom severity in TD symptoms [67]. As for its temporal specificity, HRH2 expression is upregulated in the blood of TD patients aged 10–12 years (FC = 1.12) [66] and in mouse striatum, Hrh2 shows a temporal expression pattern corresponding to developmental stages that could be linked to TD occurrence and resolution (i.e., first upregulation, then downregulation) [248]. In addition, HRH2 is highly expressed in the striatum (in the neonatal period, early infancy, early childhood and adolescence) and cortex (neonatal period, early infancy, early childhood, adolescence and early adulthood). Moreover, HRH2 has molecular specificity for TD, as it binds and interacts with HDC, the cytoplasmic enzyme that has been linked to TD by strong genetic evidence [32,269,270] and the results from our analyses (Table S2) and that converts histidine to the HRH2 ligand histamine. In addition, HRH2 regulates the production of arachidonic acid, one of the top metabolites emerging from the PRS-based analyses (see above) and it upregulates the expression of IL17A, a cytokine that itself has several interactions in the landscape and of which the blood levels were found to be increased in TD patients [271,272]. Furthermore, HDC uses PLP as its cofactor to synthesize histamine from histidine, with both PLP and histidine being implicated in TD through our analyses (see above). Histamine (see above) is also transported in and out of astrocytes and presynaptic neurons by the landscape transporters SLC22A3 and SLC29A3, while in astrocytes, it is also methylated and, hence, inactivated by the HNMT enzyme [273]. The blood expression of HNMT was also found to be upregulated is TD patients aged 13–16 (FC = 1.70), while that of SLC22A3 was downregulated in TD patients aged 10–13 (FC = −1.10) and positively correlated with TD severity [66,67], further suggesting a dysregulation of histamine metabolism in TD. Specifically, and linked to the putative modulatory specificity of HRH2, low brain concentrations of its ligand histamine have been reported in the *Hdc* knockout (KO) mouse model of TD, and histamine repletion ameliorated tic-like stereotypical movements in these animals [274]. In addition, histamine bound to HRH2 has been shown to have a neuroprotective effect by alleviating the NMDA glutamate receptor-induced excitotoxicity via cAMP signaling [275]. Moreover, the histamine precursor histidine was shown to promote astrocyte migration and provide neuroprotection through HRH2 [276]. Histidine and HNMT inhibitors also ameliorated methamphetamine-induced stereotyped behavior and behavioral sensitization in rodent models, while HDC inhibitors and HRH2 antagonists enhanced this behavior [277,278,279,280,281,282,283]. As histamine does not cross the BBB [284], a potential strategy to increase the brain levels of histamine—that could then bind and signal through HRH2—would be to provide additional histidine through the diet. Histidine is transported across the BBB by SLC3A2 and SLC7A5 that form heterodimers [285] and are both upregulated in TD postmortem striatum [64]. This being said, further studies are needed to assess the effects of histidine supplementation and/or administering agonists of HRH2 other than histamine (that pass the BBB) on TD symptoms.

Our study should be viewed in the context of a number of strengths and limitations. Particular strengths are that, to our knowledge, we have analyzed all available omics data for TD for the first time and integrated the results from these analyses with an extensive literature search for interactions between the TD-linked genes/proteins and metabolites into a molecular landscape of the disease. In turn, this TD landscape not only provides insights into the altered molecular processes that underlie the disease but also, and importantly, it enabled the identification of biologically meaningful drug target leads for further studies. An important limitation of the study was that because of the lack of omics data for specific TD symptoms, we decided to use a ‘broad’ definition of the TD phenotype, and because of this, we could not derive any insights about the molecular mechanisms underlying these specific symptoms from our results. Moreover, a number of omics studies of which we used the data were limited in sample size and therefore likely underpowered for a meaningful statistical analysis of the individual study results. However, we tried to address this by prioritizing those genes/proteins for building the landscape that have been implicated in TD through one type of genomic evidence and at least one other type of genomics or other omics evidence (i.e., the ‘dark blue’ genes/proteins). Another limitation is that the PRS-based analyses that we conducted only consider the joint effect of (very) many common genetic variants associated with TD, but further studies are needed to elucidate whether and to what extent rare genetic variants (also) contribute to metabolite levels. In addition, more advanced methods would need to be applied to dissect the ‘broad’ PRS-based signal containing hundreds of thousands of SNPs into genetic loci and even individual genes. Furthermore, the PRS-based approach only represents a starting point for identifying genetically determined levels of blood or CSF biomarkers, and further studies using for example Mendelian Randomization and metabolomics could help identify any causal or pleiotropic effects of specific metabolites on TD, and vice versa [286]. Lastly, both the protein–protein interaction databases that we used and the extensive literature search that we conducted for building the landscape are—by default—incomplete and more interactions may become known and be experimentally validated in the future. Therefore, additional studies are required to follow up on and validate/corroborate the main findings and leads from our landscape. For example, future in vivo and interventional studies could be carried out that modulate metabolites and/or interactions between genes/proteins and metabolites through existing medications or dietary changes, and that may provide new ways to lessen the burden of TD.

## 4. Materials and Methods

### 4.1. Literature Search and Selection of Omics Datasets

We searched public databases (i.e., PubMed and Europe PMC [287], which include peer-reviewed articles and preprints, and GWAS Catalog [56]) for human ‘omics’ studies in subjects with Tourette’s disorder (TD) or tic disorders, as well as pediatric acute onset neuropsychiatric syndrome (PANS) including pediatric autoimmune neuropsychiatric disorders associated with streptococcal infection (PANDAS), which have been proposed as etiological subtypes of TD [288]. The ‘omics’ notion refers to system-wide data derived from high-throughput assays measuring simultaneously all or nearly all molecules of the same type at the various level of cellular functions. These comprise: (I) genomics—the study of sequence level DNA variation associated with the disorder that can be identified through linkage analysis in family-based studies and through association studies in family and population-based data, including (a) common single-nucleotide sequence variation (base changes/substitutions/insertions/deletion), referred to as single-nucleotide polymorphisms (SNPs), found at frequencies greater than 1% in a population and investigated in genome-wide association studies (GWASs), (b) rare single-nucleotide variants (SNVs), usually exceedingly rare or unique to an individual, investigated in next-generation sequencing (NGS) studies, most often focused on protein-coding regions of the genome, known as exome sequencing, (c) rare structural variation, including copy number variation (CNV), referring collectively to differences that are at least 50 bp in length between two individual genomes; (d) chromosomal aberrations; (II) epigenomics—the study of non-sequence-level DNA modifications (changes that are heritable and do not entail a change in DNA sequence [289]), including: DNA methylation, histone modifications, and chromatin modelling; (III) transcriptomics—the study of RNA transcript abundance and expression using microarrays or RNA sequencing (RNA-seq), including protein-coding messenger RNA (mRNA) and two types of noncoding RNA with regulatory roles: long noncoding RNA (lncRNA) and microRNA (miRNA), with the latter also recognized as a type of epigenetic machinery; (IV) proteomics—the study of protein abundance and expression using antibody-based arrays and mass spectrometry (MS); (V) metabolomics—the study of low-molecular-weight compounds (metabolites) using techniques such as liquid chromatography–mass spectrometry (LC-MS) or nuclear magnetic resonance spectroscopy (NMR); (VI) microbiomics—the study of fecal microbiota as a proxy for microbiota of the gastrointestinal tract (gut microbiota). Microbiome studies were included if they reported bioactive microbiota-derived metabolites related to the alternations in the microbial composition, as microbial metabolites could affect brain activity in the microbiota–gut–brain bidirectional communication [290]. We conducted this literature search for eligible data up to 1 December 2021; otherwise-eligible studies published after this date were not included in our analyses. Additionally, reference lists from reviews were used for reference checking. Studies were excluded if (i) the study was published in a language other than English, (ii) the molecules assayed or analyzed were limited to those in candidate genes/molecules, (iii) data were not accessible. Findings considered significant in the primary publication by the study authors, using their experimental design and thresholds, are included in our main lists, while subthreshold findings are listed in the extended lists. 

### 4.2. Analyses of TD GWAS summary Statistics

We obtained the summary statistics of the largest TD GWAS meta-analysis of 14,307 individuals from the PGC website (https://www.med.unc.edu/pgc/, accessed on 24 March 2021). Prior to further analyses, we applied filters to the summary statistics, as implemented in the munge_sumstats.py script (version 1.0.1), available within the LD score regression package (ldsc) [291]; https://github.com/bulik/ldsc, accessed on 10 April 2021): (a) imputation quality INFO score above 0.9; (b) sample MAF above 1%; (c) remove indels and structural variants; (d) remove strand-ambiguous SNPs; (e) remove SNPs whose alleles do not match the alleles in 1000 Genomes. 

To better understand the mechanism by which variations at GWAS loci influence susceptibility to TD, we used different methods to dissect GWAS loci and identify TD-relevant genes. We focused on strategies aiming to prioritize genes rather than variants to arrive at more interpretable results.

#### 4.2.1. Genome-Wide Gene-Based Analysis in MAGMA

We used Multi-marker Analysis of GenoMic Annotation (MAGMA), v1.09 [292], to perform gene-based analysis of the TD GWAS meta-analysis summary statistics. MAGMA combines multiple variants that are mapped to a gene, while adjusting for the linkage disequilibrium (LD) between those variants and tests the joint association of all variants in the gene with the phenotype. This approach reduces the number of tests that need to be performed and enables the identification of effects consisting of multiple weaker associations that would be missed in the individual variant analysis. Specifically, for each of 19,427 protein-coding genes included in the NCBI 37.3 database, we considered all single-nucleotide polymorphisms (SNPs) located in the gene body (0 kb) and 100 kb windows on both sides. Then, using an updated SNP-wise Mean model, we combined the resulting SNP *p*-values into a gene test statistic (the sum over squared SNP Z-statistics) and computed the corresponding gene *p*-value. We used the 1000 Genome Project Phase 3 European population as reference data to account for the LD-induced covariance of SNP *p*-values. For further analyses, we considered genes with the *p*-value < 1.0 × 10^−3^, a less stringent cut-off to enable the retrieval of suggestive associations. Previous studies have shown that SNPs in (the vicinity of) a gene with sub-threshold *p*-values as high as 1.00 × 10^−4^ are likely to carry a significant biological signal affecting gene expression and function and may reach significance in later higher-powered studies [293,294].

#### 4.2.2. SNP Functional Annotation and Gene Mapping in FUMA

- SNP functional annotation

We used the FUMA online platform (v1.3.6b, [295], http://fuma.ctglab.nl/, accessed on 16 August 2021) for the identification of genomic risk loci and functional annotation of SNPs from the TD GWAS meta-analysis summary statistics. We first identified independent significant SNPs with a *p*-value < 1.0 × 10^−5^ and which are independent of each other at r^2^ < 0.6. These SNPs were further represented by lead SNPs, which are a subset of the independent significant SNPs in approximate linkage equilibrium with each other at r^2^ < 0.1 (based on LD information calculated from 1000 genomes). We then defined associated genomic loci by merging any physically overlapping lead SNPs (linkage disequilibrium (LD) blocks < 250 kb apart). We selected all candidate SNPs in associated genomic loci that were in LD (r^2^ > 0.6) with one of the independent significant SNPs, had a *p*-value < 5 × 10^−2^ and minor allele frequency (MAF) > 0.0001, for functional annotation. Functional consequences for these SNPs were obtained by matching SNPs’ chromosomes, base-pair positions, and reference and alternate alleles to databases containing known functional annotations, including ANNOVAR categories [296], Combined Annotation-Dependent Depletion (CADD) scores [297], RegulomeDB scores [298], and chromatin states [299,300]. ANNOVAR categories identify the SNP’s genic position (e.g., intron, exon, and intergenic) and associated function. CADD scores predict how deleterious the effect of a SNP is likely to be for a protein structure/function, with higher scores referring to higher deleteriousness. A CADD score above 12.37 is the threshold to be potentially pathogenic [297]. The RegulomeDB score is a categorical score based on information from expression quantitative trait loci (eQTLs) and chromatin marks, ranging from 1a to 7, with lower scores indicating a higher probability of having a regulatory function.

- Gene mapping

Subsequently, we used FUMA to map functionally annotated SNPs to genes by combining three mapping strategies: positional, eQTL and 3D chromatin interaction mappings. For positional mapping, SNPs were mapped to known protein-coding genes in the human reference assembly (GRCh37/hg19) based on the physical distance of 10 kb windows on both sides. For eQTL and chromatin interaction mappings, we performed analyses (1) across all available tissue/cell types—enabling full extracting of possible candidate genes and (2) within brain—to prioritize brain-specific candidate genes by eQTLs and chromatin interactions. Specifically, for brain-specific eQTL mapping, we used only brain-related eQTL data available within FUMA: eQTL Catalogue [301]: BrainSeq (DLPFC) [302] and Schwartzentruber_2018 (Sensory neurons) [303], PsychENCODE (PFC, TC, CB) [304], xQTL (DLPFC) [305], The CommonMind Consortium (CMC) (DLPFC) [306], BRAINEAC (10 brain regions) (http://www.braineac.org/), and GTExv8 Brain (13 brain regions). We used a false discovery rate (FDR) *p*-value of 5 × 10^−2^ to define significant eQTL associations. FUMA annotates those significant eQTLs with candidate SNPs and those SNPs are mapped to the gene whose expression is potentially affected by the SNPs. In brain-specific chromatin interaction mapping, we identified significant chromatin loops (FDR *p*-value < 1.0 × 10^−6^) using built-in chromatin interaction data from: the dorsolateral prefrontal cortex and hippocampus [307], adult and fetal cortex [308], prefrontal cortex from PsychENCODE [304], FANTOM5 [309]. In FUMA, the candidate SNPs are required to be overlapped with one end of the loop and transcription start sites (TSS) of genes (500 bp up- and 250 bp downstream from the TSS) with the other end of the loop to be mapped. Since HiC is designed to measure the physical interactions of two genomic regions, not all significant loops necessarily contain functional interactions. We further limited chromatin interaction mapping to those where SNPs overlap with enhancer regions and gene TSSs overlap with promoter regions predicted by Roadmap consortium (http://egg2.wustl.edu/roadmap/data/byDataType/dnase/). In brain-specific analyses, we used only E053-E082 brain [300] for those annotations. For all analyses, we also performed additional filtering of SNPs based on functional annotations (CADD and RegulomeDB), as it affects gene prioritization (setting a CADD score threshold will cause FUMA to use only highly deleterious SNPs or filtering SNPs by RegulomeDB score prioritizes SNPs which are likely to affect regulatory elements per one of the mapping strategies).

#### 4.2.3. Transcriptome-Wide Association Study

Under the assumption that the effect of genetic variation on a phenotype is mediated by gene expression, we performed a transcriptome-wide association study (TWAS) to integrate TD GWAS meta-analysis summary statistics and cis-eQTL signals and prioritize candidate risk genes for TD. TWAS was implemented in FUSION [310] using the FUSION.assoc.test.R script with default settings over all autosomal chromosomes. Pre-computed SNP-expression weights from all tissue reference samples from GTEx Consortium (GTEx v7) were obtained from the FUSION website (http://gusevlab.org/projects/fusion/, accessed on 23 April 2021). We applied this recommended agnostic approach to scan all tissues models to improve our ability to detect relevant regulatory mechanisms that mediate the phenotypic association [311]. To discover genes whose expression is regulated by the same variants that underlie GWAS hits, we performed colocalization analysis using the interface to the coloc R package [312] available in FUSION for all genes below TWAS *p*-value threshold of 5 × 10^−4^ (Fusion.assoc_test.R--coloc_P flag). This Bayesian approach evaluates the posterior probability (PP) that genetic associations within a locus for two outcomes are driven by a shared causal variant. It enables the distinction between associations driven by horizontal pleiotropy (1 causal SNP affecting both gene expression and phenotype; posterior probability PP4) and linkage (2 causal SNPs in LD affecting gene expression and phenotype separately; posterior probability PP3). Significant features were considered as colocalized based on their low PP3 (<0.2) used as a less stringent threshold for evidence of non-independent association signal, as applied previously [313]. Of note, while TWAS tests for association between gene expression and a phenotype, it accounts only for genetically predicted expression (common cis eQTLs) and constitutes only a small fraction of total expression that also includes environmental and technical components [314].

#### 4.2.4. Shared Genetic Etiology Analyses with Levels of Blood and Cerebrospinal Fluid Metabolites

- Polygenic risk score (PRS)-based analyses

Polygenic risk score (PRS) is used to summarize the aggregated risk from common variants across the genome, and it is a valuable tool for comparing the shared genetic basis of different traits. To test for genetic sharing between TD and levels of blood metabolites, cytokines and metals, as well as levels of CSF metabolites, we performed polygenic risk score (PRS)-based analyses in PRSice (v1.25) [315] using the summary-summary statistic based approach. As ‘base phenotype’, we used TD GWAS meta-analysis summary statistics. As ‘target phenotypes’, we used publicly available GWAS summary statistics for a total of 993 blood (serum and/or plasma) traits reported in six separate studies, including 941 metabolites [23,77,78,80], 41 cytokines [76] and 11 metals [316], as well as 338 CSF metabolic traits [79]. First, we performed clumping in PLINK (v1.90) [317] to remove SNPs in linkage disequilibrium (LD, based on R^2^ > 0.25 within 500 kb window) with the SNP with the smallest *p*-value in the base phenotype and generated sets of independent SNPs. Subsequently, we calculated PRS in PRSice using clumped SNPs whose *p*-value in the base phenotype were below seven broad *p*-value thresholds (P_T_) (0.001, 0.05, 0.1, 0.2, 0.3, 0.4, and 0.5) to select the one that maximized the variance explained (R^2^) for the base phenotype in the target phenotypes. PRS are estimated as a sum of risk alleles across SNPs with GWAS *p*-values below a given *p*-value threshold, weighted by the effect sizes estimated by the GWAS. Finally, we performed regression to test the association between the PRS and target phenotypes, i.e., the extent to which combined SNPs from each of the seven P_T_-linked PRS for TD predict each of the target phenotypes (993 blood and 338 CSF metabolic traits’ levels). To account for the large number of tests, we applied Bonferroni correction and set *p*-value thresholds of 7.19 × 10^−6^ (0.05/(993 tests × 7 P_Ts_)) and 2.11 × 10^−5^ (0.05/(338 tests × 7 P_Ts_)) to designate statistically significant results for blood and CSF metabolic traits, respectively. We also calculated Benjamini–Hochberg-adjusted (FDR) *p*-value and set a less stringent cut-off of FDR *p*-value < 1 × 10^−2^ to retrieve suggestive associations [318].

- SNP effect concordance analyses (SECA)

For the statistically significant findings from the PRS-based analyses, we performed SNP Effect Concordance analysis (SECA) [319] to test for the genetic concordance (i.e., the same SNP effect directions across both traits) between TD and blood/CSF metabolite levels. We applied Bonferroni correction to account for the number of tests performed in SECA and to designate statistically significant results. 

### 4.3. Integration, Annotation, and Prioritization of Omics Studies Results

#### 4.3.1. Integration of Omics Studies Results

We unified gene symbols and Entrez Gene identifiers (Entrez ID) across different genomic, transcriptomic and epigenomic datasets using ‘HGNChelper’ [320,321] and ‘org.Hs.eg.db’ [322] R packages. The ‘HGNChelper’ package identifies known aliases and outdated gene symbols based on the HUGO Gene Nomenclature Committee (HGNC) database [323], as well as common mislabeling introduced by spreadsheets, and provides corrections where possible. We used the most current available maps of aliases for correcting gene symbols. The ‘org.Hs.eg.db’ annotation package extracts Entrez Gene identifiers for gene symbols using data provided by Entrez Gene ftp://ftp.ncbi.nlm.nih.gov/gene/DATA (date stamp from the source: 13 September 2021). Gene lists were merged into a master table by unified Entrez ID and gene symbol. Genes for which Entrez ID was not identified were not included in our subsequent analyses. All analyzes were conducted in R [324]. 

We unified metabolite names across different studies using information from the Human Metabolome Database (HMDB) [325]. Some metabolites were classified as ‘Unknown’, indicating that their chemical identity was not yet determined at the time of analysis. Metabolites were assigned to metabolic groups—superpathways (amino acids, carbohydrates, cofactors and vitamins, energy, lipids, nucleotides, peptides, and xenobiotic metabolism) and pathways, based on the description in the Kyoto Encyclopedia of Genes and Genomes database (KEGG) [326]. Given that some metabolites are differently preserved in blood plasma and serum, and that platforms may differ in extraction protocols, we examined all metabolites included in the original studies and have not selected the largest sample available for a particular metabolite. 

#### 4.3.2. Gene-Level Annotation of Omics Studies Results

We annotated genes with a set of molecular features that would facilitate building of the molecular landscape and provide a rationale to further prioritize genes for therapeutic targeting. Specifically, we used information contained in the UniProt Knowledgebase (UniProtKb) [327] (http://www.uniprot.org, accessed on 27 September 2021) to extract functional and subcellular localization annotations for genes/proteins. We used Human Protein Atlas (HPA) version 21.0 [268] (http://v21.proteinatlas.org, accessed on 18 February 2021) to obtain data on tissue and cell expression of RNA/protein, as well as their subcellular location. Genes were considered to be expressed in the brain if they were detected on at least RNA level in the mammalian brain (integrated data from human, pig and mouse). In HPA, protein expression is based on immunohistochemical data. Each subcellular location is given one of the four reliability scores (Enhanced, Supported, Approved, or Uncertain) based on available protein/RNA/gene characterization data from both HPA and the UniProtKB/Swiss-Prot database. Furthermore, we extracted information on the potential functional importance of a gene/protein, such as essentiality and druggability. A gene is considered essential when it is indispensable for the reproductive success of an organism and, thus, the loss of its function compromises the viability or fitness of the organism. In humans, essentiality is estimated based on loss-of-function (LoF) mutation intolerance, either from population exome sequencing (in vivo) data—statistical estimates of unexpected mutational depletion identify genes presumed to be subjected to functional constraints [328]; or (2) CRISPR-based in vitro perturbation experiments—systematic testing of gene-silencing effects on human cell cultures identifies genes that affect cell viability or optimal fitness upon perturbation. To this end, we used human gene essentiality estimations based on different measures of tolerance to LoF mutations provided by Bartha et al., 2018 [329] (extracted from Supplementary Information S2). Estimates include the following scores based on the Exome Aggregation Consortium (ExAC) sample of 60,706 human exomes [328]: residual variation intolerance score (RVIS) [330], Evo-Tol [331], missense Z-score [332], LoFtool [333], probability of haploinsufficiency (Phi) [334], probability of loss-of-function intolerance (pLI) [328] and selection coefficient against heterozygous loss-of-function (shet) [335]. Scores based on cell culture perturbation-based experiments include data from KBM7, Raji, Jiyoye, HCT116 and K562 cell lines [336]; the KBM7 cell line [337], and RPE1, GBM514, HeLa and DLD1 cell lines [338]. Furthermore, we used information on gene druggability that could give scope for drug repurposing or redesign. The druggable genome can be defined as the genes/gene products known or predicted to interact with drugs, ideally with a therapeutic benefit to the patient. To prioritize druggable genes, we used the list of 4479 genes defined by Finan et al. as the ‘druggable genome’ ([339], provided in Table S1). Genes reported by Finan et al. are divided into 3 tiers corresponding to their position in the drug development pipeline: Tier 1 contains genes encoding targets of approved or clinical trial drugs; Tier 2 genes encoding targets with high sequence similarity to Tier 1 proteins or targeted by small drug-like molecules; and Tier 3 contains genes encoding secreted and extracellular proteins, genes encoding proteins with more distant similarity to Tier 1 targets, and genes belonging to the main druggable gene families not already included in Tier 1 and Tier 2 (GPCRs, nuclear hormone receptors, ion channels, kinases, and phosphodiesterases).

#### 4.3.3. Prioritization of Omics Studies Results

Since the DNA sequence remains unaltered throughout life and is not influenced by environment or development (apart from somatic mutations), genetic variants associated with the disorder are thought to contribute to/precede, and not be a consequence of, disease development. Moreover, previous studies have shown that drug candidates are more likely to pass clinical trials and be approved for patients if they target genes linked to human disease [13,14], highlighting the importance of human genetics in target identification and drug discovery. Given the above, we prioritized the results of genomics studies of TD and used them as an anchor point for further analyses exploring molecular mechanisms implicated in TD etiology and modeling interactions of other omics data. Specifically, we compiled the primary list of TD candidate genes for enrichment analyses (referred to as ‘TD candidate genes’ throughout the text), which included genes reported as primary significant findings (main lists) from: GWAS-based analyses (MAGMA, FUMA, TWAS, and cross-disorder), preliminary MAGMA of the newest TD GWAS, rare single-nucleotide variants (SNVs), copy number variations (CNVs), chromosomal aberrations, as well as genes reported as subthreshold findings (included in the extended lists)—only if they were reported in at least two separate studies. We classified studies based on assigned evidence level as: guiding (genomics studies), corroborating (epigenomic and transcriptomic studies) and additional (metabolomics, microbiome). Of note, given the paucity of data, we decided not to consider evidence about the (putative) regulations of mRNAs/proteins by miRNAs. 

### 4.4. Tissue and Cell Type Specificity Analyses

To test the assumption that genes associated with disease are more likely to be highly expressed in the tissues and cells afflicted by the disease, we performed tissue and cell type specificity analyses. 

We used the Tissue-Specific Expression Analysis (TSEA, v1.0: Updated 3 March 2014) [340] and the Cell-Specific Expression Analysis (CSEA) [341,342] web tools to test whether genes preferentially expressed in any given tissue or cell type were enriched in the set of 872 TD genes. For the TSEA, we used the gene expression data for 25 broad human tissue types derived from the Genotype-Tissue Expression (GTEx) project [343] and human brain region- and time-specific gene expression RNA seq data obtained from the BrainSpan Atlas [344]. For the CSEA, we used the mouse-cell-type-specific gene expression profiling experiments that were conducted on a single platform, most using published translating ribosome affinity purification (TRAP) data, as described in [342]. The TRAP method estimates a rate of protein synthesis and is a better predictor of actual protein levels than measurements of mRNA levels [345]. The specificity of expression was represented as a specificity index probability (pSI) statistic at thresholds 0.05 to 0.0001, with a smaller value indicating higher specificity. For details on pSI score calculation, we refer to the original publication [340]. We considered genes with pSI statistics smaller than 0.05 as significantly enriched in the tissue or cell type. The overlap between TD genes and the genes enriched in each tissue or cell type was estimated using Fisher’s exact test followed by false discovery rate (FDR) correction with Benjamini–Hochberg method. The significance threshold was defined as FDR *p*-value < 5 × 10^−2^. 

We additionally applied CSEA to the results of differential expression analysis of postmortem transcriptome data from the striatum of TD patients [64] to investigate which cell types are particularly affected by the lifelong TD. These analyses were performed separately for down- and up-regulated genes from combined analysis of caudate and putamen, as well as for the top modules from the weighted gene co-expression network analysis (WGCNA) that were most significantly enriched for down- and up-regulated genes. Such joint analyses can further improve the power to detect cellular composition alterations from transcriptomic data [74].

### 4.5. Functional Enrichment Analyses

We used Ingenuity Pathway Analysis (IPA) software (QIAGEN, Hilden, Germany) to identify canonical pathways, diseases and functions, and upstream regulators that were enriched in the set of 872 TD candidate genes. The significance of the association between our dataset and the given pathway, disease/function, and upstream regulator was measured using the right-tailed Fisher’s Exact Test, followed by false discovery rate (FDR) calculation using the Benjamini-Hochberg method to correct for multiple-testing. A threshold of FDR *p*-value < 5 × 10^−2^ (−log (FDR *p*-value) > 1.3) was used to designate statistically significant findings, while results with unadjusted *p*-value < 1 × 10^−2^ are reported as suggestive associations. For statistical calculations, all genes associated with pathways, functions, and regulators in the Ingenuity Knowledge Base (IKB) were used as the reference set.

Canonical pathways are well-characterized metabolic and cell signaling cascades derived from the literature and public and third-party databases compiled in the IKB. For canonical pathways, apart from the *p*-value of overlap, a ratio indicating the strength of the association is also provided (the number of genes from the dataset that map to the pathway divided by the total number of genes that map to the canonical pathway). Pathways with high ratios and low *p*-values may be the most likely candidates for an explanation of the observed phenotype.

In the diseases and functions analysis, IPA identifies diseases and biological processes associated with the dataset based on the prior knowledge of expected causal effects between genes/proteins and the diseases/functions contained in the IKB. We report results organized in the three main categories: ‘Diseases and Disorders’, ‘Molecular and Cellular Functions’, and ‘Physiological System Development and Function’, along with the FDR *p*-value of overlap and (number of) molecules associated with each function.

Upstream regulator analysis identifies ‘upstream regulators’—molecules that may control the expression of target genes in our dataset, based on the expected causal effects derived from the literature. We report upstream regulators classified into two main groups: ‘Drugs and Chemicals’ and ‘Genes, RNAs and Proteins’.

### 4.6. Molecular Landscape of TD

First, we filtered the TD candidate genes (see above) based on the number of lines of supporting evidence, prioritizing genes that (a) are present in at least two independent main genetic lists (studies/analyses) or where a genetic finding had corroborating evidence in epigenetic or transcriptomic studies (requiring evidence from two independent blood studies or one brain study), (b) genes that are expressed in brain (tissues) based on the data in HPA, and (c) genes that are protein-coding. This step resulted in the list of TD ‘prioritized’ candidate genes and their encoded proteins that we focused on for building the landscape, with the remaining genes/proteins from the candidate list—for which less omics evidence was available—only being used in a second stage (see below).

Second, we filtered the metabolites linked to TD through the PRS-based analyses and/or (other metabolome/microbiome studies), based on the strength of the supporting (genetic) evidence. We included HMDB-annotated metabolites implicated through PRS-based analyses with (a) a Bonferroni-adjusted *p*-value < 5 × 10^−2^, (b) an FDR *p*-value < 1 × 10^−2^, if they were also implicated through other metabolome/microbiome studies, or (c) an FDR *p*-value < 5 × 10^−2^, if they were replicated in PRS-based analyses and implicated through metabolome/microbiome studies. We also included metabolites linked through PRS-based analyses (FDR *p*-value < 5 × 10^−2^) or metabolome/microbiome studies if they could be directly linked to a TD-associated protein through a functional or metabolic interaction (e.g., the TD-associated protein is a transporter or receptor for the metabolite). 

Subsequently, to build the actual molecular landscape of TD, we applied an approach that was used previously for other neuropsychiatric diseases [20,346]. The UniProt Protein Knowledge Base (http://www.uniprot.org, accessed on 27 September 2021) [327] was used to gather basic information on the function(s) and subcellular localization(s) of all the landscape candidate genes/proteins. We also used PubMed (https://pubmed.ncbi.nlm.nih.gov) to identify the functional, experimental evidence-based interactions between the landscape candidate proteins. This included assembling protein–protein interactions (PPIs) and protein–metabolite interactions data from several literature-curated resources that contain high-quality interactions with experimental evidence. These included primary and secondary databases, such as the Ingenuity Knowledge Base available in IPA, OmniPath [347], The Human Reference Interactome (HuRI) [348] (http://www.interactome-atlas.org, accessed on 18 April 2022), High-quality INTeractomes (HINT) [349] (http://hint.yulab.org, accessed on 11 April 2022), and The Integrated Interactions Database (IID) [350] (http://iid.ophid.utoronto.ca, accessed on 17 April 2022). These protein–protein interaction resources differ in the number and types of relationships they capture, e.g., physical binary interactions, enzymatic reactions, or functional relationships, and taken together, the resources provide good coverage of the protein interactome. From this interactome data, we then selected the interactions between the proteins encoded by the prioritized TD candidate genes (see above) as well as—and in a second stage—between proteins encoded by prioritized TD candidate genes and proteins encoded by other genes from the list of candidate genes for which less omics evidence was available, proteins/genes implicated in TD through transcriptomics data and/or other functional evidence, as well as the metabolites emerging from our PRS-based analyses.

Furthermore, we annotated interacting proteins with their contextual information, including cell expression from HPA and subcellular localization from UniProtKb. Biological processes carried out by interacting proteins are separated in the cellular and subcellular space, which helps their precise regulation [351]. Therefore, we (also) curated assembled interactome data to retain interactions that are biologically likely to occur in a given (sub)cellular location. For example, if in a binary interaction both proteins did not share the same localization or at least one compartment in multiple localized proteins, the interaction was ruled out as likely not occurring, an approach that has been used before [352]. Furthermore, all self-interactions were removed and not considered for the landscape. In addition, we determined the most likely cell type for each interaction based on the expression profiles contained in the HPA and the results of our cell type specificity analyses. 

Lastly, we used the program Serif DrawPlus version 4.0 (www.serif.com, Nottingham, UK) to draw the figure depicting the molecular landscape of TD. We tried to avoid repetitive drawing of a protein or protein–protein interactions as much as possible. If multiple locations of a protein–protein interaction were possible, functional interaction and/or expression data or other protein–protein interactions were used to identify the (most) appropriate location.

### 4.7. Selection of Putative Drug Targets from the Built Molecular Landscape of TD

After building the molecular landscape, we selected some putative drug targets based on four broad aspects of target specificity. First, a good drug target should be highly expressed in the (brain) tissues and cell types that are affected in the disease [353,354] (in this case TD)—and preferably differentially expressed in comparison with healthy controls—constituting the regional specificity of the target. To evaluate this aspect of target specificity, we analyzed the available postmortem brain data, although the differential expression of the genes/proteins in these data may cause TD or represent a consequence of TD (including compensatory mechanisms). Linked to the regional specificity, putative drug targets should also be temporally associated with the onset and/or progression of TD. To assess this temporal specificity, we again looked at the available data, including transcriptional data during striatal development [248]—which correspond to different stages of brain development and function that in turn could be linked to TD symptom occurrence, peak and resolution—and temporal gene expression data in (normal) brain tissue [344] and the blood of TD patients [66]. A third aspect of an ideal drug target for TD—that is linked to the molecular landscape—is its molecular specificity, i.e., whether it is involved in (multiple) biological processes and protein interactions in the landscape. Lastly, a suitable drug target needs to have sufficient modulatory specificity, in that it should be inherently druggable [339] and modulating the target in a certain direction—e.g., because disease-associated variants are eQTLs that (up- or down-) regulate the expression of the target [355], which is especially the case for essential genes that are relatively depleted for eQTLs [356]—has a (putative) beneficial aspect on TD (symptoms).

## 5. Conclusions

In conclusion, through integrating the results from multiple analyses of TD-linked genes derived from different types of omics data with an extensive literature search, we built a molecular landscape of TD. This landscape provides insights into the altered subcellular, molecular, and metabolic pathways and processes that are underlying the disease, including cAMP signaling, endocannabinoid signaling, multiple metabolic pathways (e.g., involving polyunsaturated fatty acids such as arachidonic acid, butyrate, NAAG, and myo-inositol), and synaptic functioning. Importantly, the landscape also yields clues towards potential drug targets (FLT3, NAALAD2, CX3CL1-CX3CR1, OPRM1, and HRH2) that can be further developed into TD treatments.

## Figures and Tables

**Table 1:** Canonical Pathways enriched within the TD candidate genes.

Canonical Pathway	FDR *p*-Value	Ratio	Dataset Genes in the Pathway
cAMP-mediated signaling	1.95 × 10^−2^	22/235	*ADCY2*, *AKAP9*, *CAMK1D*, *CRHR1*, *DRD2*, *DUSP6*, *FFAR3*, *FPR1*, *FPR2*, *GRK4*, *HRH2*, *MAPK3*, *MPPE1*, *OPRD1*, *OPRK1*, *OPRM1*, *PALM2AKAP2*, *PDE4A*, *PDE6B*, *PDE9A*, *PRKAR2A*, *RGS12*
Endocannabinoid Neuronal Synapse Pathway	2.19 × 10^−2^	16/149	*ADCY2*, *CACNA1D*, *CACNA1I*, *CACNA1S*, *CACNA2D3*, *DAGLA*, *DNAH1*, *DNAH10*, *DNAH3*, *GNB1L*, *GRIN2A*, *MAPK3*, *PLCH1*, *PRKAG1*, *PRKAR2A*, *RIMS1*

**Table 2:** Biological functions enriched within TD candidate genes.

Molecular and Cellular Functions			Physiological System Development and Function		
Functional Annotation	FDR *p*-Value	Genes	Functional Annotation	FDR *p*-Value	Genes
Cell movement of neurons	5.73 × 10^−9^	40	Cognition	8.33 × 10^−12^	72
Development of neurons *	2.00 × 10^−8^	92	Learning	4.23 × 10^−9^	62
Neuritogenesis *	2.03 × 10^−8^	48	Morphology of nervous system	9.54 × 10^−9^	107
Migration of neurons *	2.11 × 10^−8^	38	Development of head	1.32 × 10^−8^	108
Development of neural cells *	2.90 × 10^−8^	95	Morphogenesis of nervous tissue	1.95 × 10^−8^	76
Neurotransmission *	7.57 × 10^−8^	54	Development of body axis	2.66 × 10^−8^	112
Organization of cytoplasm	2.01 × 10^−7^	148	Morphology of brain	2.94 × 10^−8^	66
Organization of cytoskeleton	4.55 × 10^−7^	136	Morphology of central nervous system	4.82 × 10^−8^	70
Microtubule dynamics	6.08 × 10^−7^	121	Organismal death	5.02 × 10^−8^	220
Formation of cellular protrusions	9.93 × 10^−7^	38	Development of central nervous system	1.29 × 10^−6^	73
Migration of neural cells	2.02 × 10^−6^	40	Formation of brain	1.54 × 10^−6^	60
Quantity of neurotransmitter	5.87 × 10^−6^	27	Abnormal morphology of brain	1.38 × 10^−5^	50
Transport of molecule	6.29 × 10^−6^	141	Spatial learning	1.44 × 10^−5^	28
Synaptic transmission *	2.07 × 10^−5^	41	Emotional behavior	2.01 × 10^−5^	38
Cell movement of brain cells	3.39 × 10^−5^	18	Conditioning	2.17 × 10^−5^	30
Axonogenesis *	3.68 × 10^−5^	37	Abnormal morphology of nervous system	2.23 × 10^−5^	81
Branching of cells	4.59 × 10^−5^	75	Morphology of head	2.26 × 10^−5^	101
Action potential of cells *	4.61 × 10^−5^	23	Morphology of nervous tissue	2.44 × 10^−5^	72
Proliferation of neural cells *	5.92 × 10^−5^	70	Abnormal morphology of central nervous system	2.46 × 10^−5^	53
Length of cells	6.46 × 10^−5^	31	Prepulse inhibition	2.68 × 10^−5^	17
Morphology of neurons *	7.13 × 10^−5^	38	Quantity of neurons	3.93 × 10^−5^	51
Sprouting	1.06 × 10^−4^	99	Vocalization	4.81 × 10^−5^	12
Proliferation of neuronal cells *	1.11 × 10^−4^	56	Vertical rearing	9.39 × 10^−5^	16
Length of neurons *	1.83 × 10^−4^	47	Movement of rodents	1.35 × 10^−4^	26
Shape change of neurites	2.85 × 10^−4^	20	Social exploration	2.34 × 10^−4^	12
Abnormal quantity of neurotransmitter	3.28 × 10^−4^	9	Social behavior	2.61 × 10^−4^	18
Branching of neurons *	3.36 × 10^−4^	18	Exploratory behavior	2.65 × 10^−4^	16
Length of neurites *	3.52 × 10^−4^	16	Abnormal morphology of body cavity	3.43 × 10^−4^	126
Migration of brain cells	3.80 × 10^−4^	15	Nest-building behavior	4.37 × 10^−4^	8
Branching of neurites *	4.15 × 10^−4^	65	Abnormal morphology of head	4.81 × 10^−4^	86
Organization of cells	5.56 × 10^−4^	22	Locomotion	5.03 × 10^−4^	39
Quantity of monoamines	5.90 × 10^−4^	24	Morphology of body cavity	6.05 × 10^−4^	139
Quantity of catecholamine	5.95 × 10^−4^	20	Development of body trunk	6.47 × 10^−4^	104
Action potential of neurons *	7.23 × 10^−4^	19	Quantity of cells	7.26 × 10^−4^	157
Uptake of dopamine	8.48 × 10^−4^	7	Self-abusive behavior	7.51 × 10^−4^	4
			Passive avoidance learning	9.72 × 10^−4^	9

Table 2 presents the enriched biological functions, the FDR *p*-value of overlap, and the number of genes involved in each function. * Functional annotations that are shared between the two main functional categories but are reported only once.

**Table 3:** Results from the PRS-based and SECA analyses for the levels of 37 blood and 2 CSF metabolites that show evidence for genetic sharing with TD at a Bonferroni-corrected *p*-value < 5 × 10^−2^.

Metabolite	Superpathway	Pathway	P_T_	*p*-Value	R^2^	N SNPs	Concordance *p*-Value	Concordance with TD	GWAS	N GWAS	Biofluid
Betaine ^a^	Amino acid	Glycine, Serine, and Threonine Metabolism	0.5	4.05 × 10^−7^	1.34%	160,355	9.99 × 10^−4^	+	Rhee	1802	P
Indoxyl sulfate ^a^	Amino acid	Tryptophan Metabolism	0.5	7.08 × 10^−6^	1.29%	160,350	9.99 × 10^−4^	-	Rhee	1455	P
Homocitrulline	Amino acid	Urea cycle; Arginine and Proline Metabolism	0.5	4.41 × 10^−6^	0.50%	135,356	9.99 × 10^−4^	-	Shin	3950	P, S
Valine ^a^	Amino acid	Valine, leucine, and isoleucine metabolism	0.4	4.44 × 10^−6^	0.30%	130,625	9.99 × 10^−4^	+	Draaisma	6538	S
Pyridoxate ^a^	Cofactors and vitamins	Vitamin B6 Metabolism	0.05	4.91 × 10^−6^	1.34%	25,642	9.99 × 10^−4^	-	Rhee	1453	P
Tumor necrosis factor-beta	Cytokine	NA	0.3	3.75 × 10^−6^	1.28%	148,461	9.99 × 10^−4^	+	Ahola-Olli	1559	P, S
Phosphatidylcholine diacyl c38:4	Lipid	Glycerophospholipids	0.4	1.54 × 10^−8^	0.41%	130,941	9.99 × 10^−4^	+	Draaisma	7474	S
Phosphatidylcholine 32:1 ^a^	Lipid	Glycerophospholipids	0.5	2.94 × 10^−7^	1.38%	160,355	9.99 × 10^−4^	-	Rhee	1797	P
Phosphatidylcholine 38:5 ^a^	Lipid	Glycerophospholipids	0.5	3.19 × 10^−7^	1.37%	160,355	9.99 × 10^−4^	+	Rhee	1797	P
1-arachidonoylglycerophosphoethanolamine *	Lipid	Glycerophospholipids	0.4	3.47 × 10^−7^	0.33%	115,933	9.99 × 10^−4^	+	Shin	7350	P, S
1-stearoylglycerophosphoethanolamine	Lipid	Glycerophospholipids	0.05	1.50 × 10^−6^	0.31%	22,467	9.99 × 10^−4^	+	Shin	6929	P, S
Phosphatidylcholine diacyl c38:6	Lipid	Glycerophospholipids	0.5	6.30 × 10^−6^	0.25%	153,336	9.99 × 10^−4^	+	Draaisma	7475	S
1-arachidonoylglycerophosphocholine *	Lipid	Glycerophospholipids	0.3	6.35 × 10^−6^	0.27%	93,688	9.99 × 10^−4^	+	Shin	7063	P, S
Phosphatidylcholine diacyl c36:4	Lipid	Glycerophospholipids	0.4	6.58 × 10^−6^	0.25%	130,937	9.99 × 10^−4^	+	Draaisma	7476	S
Myo-inositol	Lipid	Inositol Metabolism	0.5	8.25 × 10^−10^	0.49%	135,380	9.99 × 10^−4^	-	Shin	7354	P, S
Total lipids in very small VLDL	Lipid	Lipid ratios	0.4	1.73 × 10^−6^	0.11%	390,060	9.99 × 10^−4^	+	Shin	2859	P, S
Concentration of very small VLDL particles	Lipid	Lipid ratios	0.2	3.27 × 10^−6^	0.11%	223,305	9.99 × 10^−4^	+	Kettunen	19,273	P, S
Phospholipids in very small VLDL	Lipid	Lipid ratios	0.4	3.37 × 10^−6^	0.11%	390,017	9.99 × 10^−4^	+	Kettunen	19,273	P, S
Sum SM	Lipid	Lipid ratios	0.3	4.21 × 10^−6^	1.10%	109,794	9.99 × 10^−4^	+	Rhee	1797	P
Ratio total PC: total LPC	Lipid	Lipid ratios	0.3	5.96 × 10^−6^	1.06%	109,794	9.99 × 10^−4^	-	Rhee	1797	P
Stearate (18:0)	Lipid	Long Chain Fatty Acid	0.2	3.46 × 10^−8^	0.39%	68,520	9.99 × 10^−4^	+	Shin	7355	P, S
X-12442—5,8-tetradecadienoate	Lipid	Long Chain Fatty Acid	0.1	8.00 × 10^−7^	0.31%	39,528	9.99 × 10^−4^	+	Shin	7334	P, S
Palmitate (16:0)	Lipid	Long Chain Fatty Acid	0.3	2.73 × 10^−6^	0.28%	93,694	9.99 × 10^−4^	+	Shin	7352	P, S
Laurate (12:0)	Lipid	Medium Chain Fatty Acid	0.1	3.77 × 10^−7^	0.33%	39,530	9.99 × 10^−4^	+	Shin	7346	P, S
Linoleate (18:2n6) ^a^	Lipid	Polyunsaturated Fatty Acid (n3 and n6)	0.2	4.67 × 10^−8^	0.39%	68,518	9.99 × 10^−4^	+	Shin	7333	P, S
Arachidonate (20:4n6)	Lipid	Polyunsaturated Fatty Acid (n3 and n6)	0.5	1.09 × 10^−7^	0.36%	135,383	9.99 × 10^−4^	+	Shin	7367	P, S
Linolenate [alpha or gamma; (18:3n3 or 6)]	Lipid	Polyunsaturated Fatty Acid (n3 and n6)	0.2	1.38 × 10^−6^	0.30%	68,524	9.99 × 10^−4^	+	Shin	7338	P, S
Dihomo-linoleate (20:2n6)	Lipid	Polyunsaturated Fatty Acid (n3 and n6)	0.3	6.47 × 10^−6^	0.26%	93,696	9.99 × 10^−4^	+	Shin	7353	P, S
Sphingomyelin 18:1 ^a^	Lipid	Sphingolipid Metabolism	0.4	3.87 × 10^−10^	2.08%	136,596	9.99 × 10^−4^	+	Rhee	1797	P
Sphingomyelin 18:0 ^a^	Lipid	Sphingolipid Metabolism	0.3	4.64 × 10^−7^	1.33%	109,794	9.99 × 10^−4^	+	Rhee	1797	P
Triacylglycerol 50:2	Lipid	Triacylglycerol	0.5	2.52 × 10^−6^	1.15%	160,355	9.99 × 10^−4^	-	Rhee	1797	P
Triacylglycerol 48:0	Lipid	Triacylglycerol	0.5	5.66 × 10^−6^	1.07%	160,355	9.99 × 10^−4^	-	Rhee	1797	P
Triacylglycerol 48:1	Lipid	Triacylglycerol	0.5	7.18 × 10^−6^	1.04%	160,355	9.99 × 10^−4^	-	Rhee	1797	P
X-11381	Unknown	Unknown	0.1	1.06 × 10^−6^	0.31%	39,527	9.99 × 10^−4^	-	Shin	7308	P, S
X-04494	Unknown	Unknown	0.4	1.21 × 10^−6^	0.47%	115,916	9.99 × 10^−4^	-	Shin	4689	P, S
X-12116	Unknown	Unknown	0.5	2.21 × 10^−6^	0.73%	135,350	9.99 × 10^−4^	-	Shin	2859	P, S
X-09706	Unknown	Unknown	0.5	3.32 × 10^−6^	0.28%	135,378	9.99 × 10^−4^	-	Shin	7256	P, S
*N*-acetyl-aspartyl-glutamate (NAAG)	Amino acid	Glutamate Metabolism	0.5	1.80 × 10^−8^	9.90%	277,618	9.99 × 10^−4^	-	Panyard	291	CSF
Butyrate (4:0)	Lipid	Short Chain Fatty Acid	0.5	2.29 × 10^−6^	6.97%	277,618	9.99 × 10^−4^	-	Panyard	291	CSF

Note: Superpathway and pathway annotations are given for metabolites with known chemical identity. Metabolites were classified as ‘Unknown’ if their chemical identity was not yet determined at the time of analysis. Abbreviations: P_T_—the most predictive SNP *p*-value threshold in the base sample (TD); R^2^—the variance explained in the target sample (metabolite levels); N SNPs—the number of SNPs; Concordance *p*-value—all concordance analyses yielded significant results (i.e., Bonferroni-corrected *p*-value < 0.05/39 tests = 1.28 × 10^−3^); Concordance with TD—direction of effect estimated in SECA: ‘+’ positive association, ‘-’ negative association; GWAS—source of the target sample phenotypes (metabolite traits) GWAS data—Ahola-Olli [76], Draaisma [77], Kettunen [78], Panyard [79], Rhee [80], Shin [23]; N GWAS—sample size of the target sample phenotypes (metabolite traits); *—indicates metabolites for which identities were inferred based on their fragmentation spectrum and other biochemical evidence; ^a^ metabolite was also measured in different datasets, but the results did not pass the Bonferroni-corrected *p*-value threshold; PC—phosphatidylcholine; LPC—lysophosphatidylcholine; VLDL—very-low-density lipoproteins; SM—sphingomyelin; NA—not available; P—plasma; S—serum; CSF—cerebrospinal fluid.

## Data Availability

All key data that support the findings of this study are available in the main text or the Supplementary Materials.

## Supplementary Materials

The following supporting information can be downloaded at: https://www.mdpi.com/article/10.3390/ijms24021428/s1.
