# Supplementary material for: Molecular Landscape of Tourette’s Disorder
Source: Int J Mol Sci. 2023 Jan 11;24(2):1428. doi: 10.3390/ijms24021428 (PMC9865021; doi:10.3390/ijms24021428)
Supplement: Supplementary file 1 [file supplementary Figure S1.pdf › supplementary Figure S1.pdf]

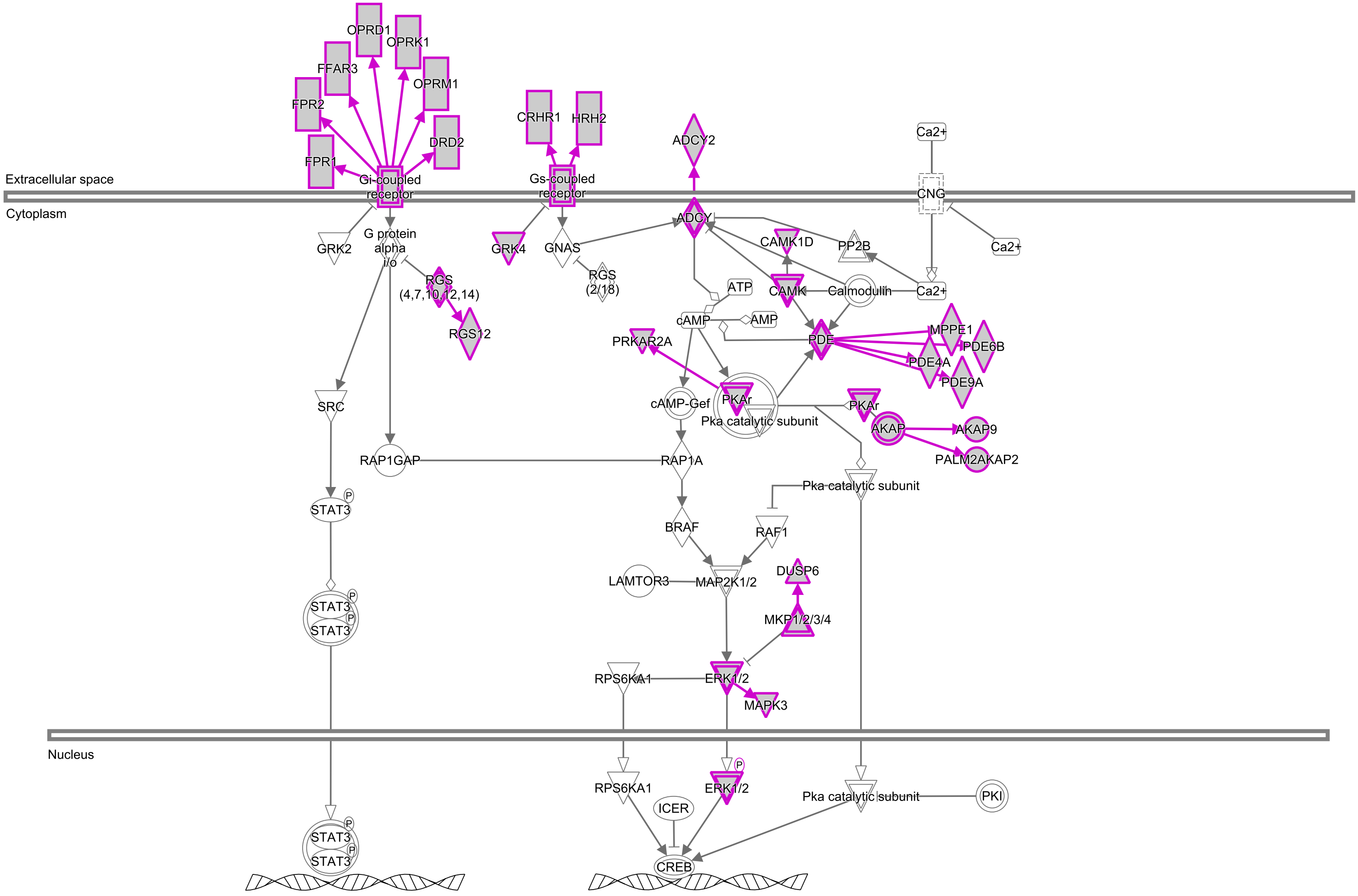

© 2000-2022 QIAGEN. All rights reserved.

Figure S1. cAMP-mediated Signaling Pathway identified by IPA
The cAMP-mediated Signaling Pathway was identified as the most significantly enriched canonical pathway in the set of 872 TD candidate genes (FDR P-value <0.05). The diagram represents the cAMP-mediated Signaling Pathway at the cellular level. It depicts the genes/proteins involved, their interactions, and the cellular and metabolic reactions in which this pathway is involved. Molecules are represented as nodes, and the biological relationship between two nodes is represented as an edge (line). Molecules highlighted with pink-outline and gray color represent genes/proteins/protein complexes which members appear in the set of 872 TD candidate genes. Abbreviations: P – phosphorylation.
