# Supplementary material for: Molecular Landscape of Tourette’s Disorder
Source: Int J Mol Sci. 2023 Jan 11;24(2):1428. doi: 10.3390/ijms24021428 (PMC9865021; doi:10.3390/ijms24021428)
Supplement: Supplementary file 1 [file supplementary File S2.pdf › supplementary File S2.pdf]

Of the inputed 872 genes,
729 genes exist in the brain region and development expression dataset, 143 genes do not, which are:
UBE2J1,TLCD4,RNU6-1228P,NELFA,HEXA-AS1,BPIFB3,URAD,PKP4,NKX2-2,GPR75-ASB3,CEP128,CERS5,NAPSB,TMEM191A,DGCR5,ZNF260,P3H2,UFD1,TMEM178B,EIF4EBP2P3,THEM7P,KMT5A,TRIQK,LINC02694,SPPL3,MTUS2,SNU13,KIRREL1,HNRNPLL,FAM193B,MARCHF7,TLNRD1,VPS9D1-AS1,SAXO2,EFL1,MTRFR,KLC1,OFCC1,ASS1P2,SNORC,COPS9,CORO7-PAM16,ICE1,GFUS,TANGO2,RTL10,LY75-CD302,RNA5SP172,NPRL2,SRSF4,KANSL1,YJEFN3,ST20-MTHFS,POM121L4P,RAMAC,KAT14,ARIH2OS,PALM2AKAP2,ATP5IF1,ARHGEF33,MAU2,DPCD,ZC2HC1C,SLC35G1,SNORA79,HYKK,PCDHGA2,CEP295NL,PEX2,RBFOX1,CDHR1,LRATD1,CFAP99,PCDHGA9,STING1,NSD3,WDPCP,CEP85L,IHO1,TENM2,C20ORF204,TENM4,P2RX6P,DYNC2I1,PROB1,CIBAR1,ESS2,SLC49A3,CFAP251,TMEM200B,MRTFA,BOLA3P2,OR8T1P,PI4KAP1,SUGP1,NOTCH2NLA,PRB1,CARF,CAMKMT,FAXC,MIR2113,SOWAHA,EXOC3L4,SEPTIN5,METTL24,CDC45,HSP90AA1,INSYN1,TADA3,IGIP,MARF1,DESI1,ABHD16A,SPATA24,DCAF12,SYCE1L,MICU3,NCKAP5,SEL1L3,GOLGA6L9,MINAR1,SELENOK,BCDIN3D-AS1,COP1,DTX2P1,NCKAP5L,SELENOF,KMT2C,COA8,KMT2D,TMEM231,SRSF7,KBTBD12,GPR42,MESD,SRSF3,MPIG6B,ST20-AS1,DOP1B,DGCR11,DCAF1,KARS1,COL6A6

Interpretation of SEA brain region and development results table:
The first row lists the pSI thresholds, and the first column lists the adult brain regions and development
The values in the tables are the Fisher's Exact p-values, followed by the Benjamini-Hochberg (BH) corrected values in parentheses.
Click the linked p-values to see the overlapped genes for the corresponding adult brain regions and development and thresholds.
The BH corrected p-values are also illustrated graphically in the figure at the end.

Brain Regions and Development and P-Values	0.05	0.01	0.001	0.0001
Amygdala.Adolescence	0.121 (0.388)	0.920 (1.000)	1.000 (1.000)	1.000 (1.000)
Cerebellum.Adolescence	0.095 (0.356)	0.619 (1.000)	0.916 (1.000)	1.000 (1.000)
Cortex.Adolescence	9.642e-04 (0.014)	0.069 (0.808)	1.000 (1.000)	1.000 (1.000)
Hippocampus.Adolescence	0.565 (0.821)	0.418 (1.000)	1.000 (1.000)	1.000 (1.000)
Striatum.Adolescence	0.248 (0.596)	0.923 (1.000)	1.000 (1.000)	1.000 (1.000)
Thalamus.Adolescence	0.027 (0.135)	0.362 (1.000)	0.568 (1.000)	1.000 (1.000)
Amygdala.Early.Childhood	0.882 (0.941)	1.000 (1.000)	1.000 (1.000)	1.000 (1.000)
Cerebellum.Early.Childhood	0.030 (0.137)	0.069 (0.808)	0.479 (1.000)	0.298 (1.000)
Cortex.Early.Childhood	0.317 (0.636)	0.654 (1.000)	1.000 (1.000)	1.000 (1.000)
Hippocampus.Early.Childhood	0.629 (0.821)	0.527 (1.000)	1.000 (1.000)	1.000 (1.000)
Striatum.Early.Childhood	0.800 (0.905)	0.485 (1.000)	1.000 (1.000)	1.000 (1.000)
Thalamus.Early.Childhood	0.609 (0.821)	0.653 (1.000)	1.000 (1.000)	1.000 (1.000)
Amygdala.Early.Fetal	0.780 (0.901)	0.970 (1.000)	1.000 (1.000)	1.000 (1.000)
Cerebellum.Early.Fetal	0.003 (0.028)	0.062 (0.808)	0.763 (1.000)	0.710 (1.000)
Cortex.Early.Fetal	0.294 (0.636)	0.258 (1.000)	0.549 (1.000)	1.000 (1.000)
Hippocampus.Early.Fetal	0.764 (0.901)	0.889 (1.000)	1.000 (1.000)	1.000 (1.000)
Striatum.Early.Fetal	0.974 (0.974)	0.875 (1.000)	0.890 (1.000)	0.166 (1.000)
Thalamus.Early.Fetal	0.558 (0.821)	0.535 (1.000)	0.436 (1.000)	1.000 (1.000)
Amygdala.Early.Mid.Fetal	0.118 (0.388)	0.895 (1.000)	1.000 (1.000)	1.000 (1.000)
Cerebellum.Early.Mid.Fetal	0.157 (0.429)	0.525 (1.000)	0.887 (1.000)	1.000 (1.000)
Cortex.Early.Mid.Fetal	1.317e-04 (0.004)	0.017 (0.808)	0.412 (1.000)	1.000 (1.000)
Hippocampus.Early.Mid.Fetal	0.023 (0.135)	0.284 (1.000)	1.000 (1.000)	1.000 (1.000)
Striatum.Early.Mid.Fetal	0.005 (0.043)	0.348 (1.000)	0.568 (1.000)	1.000 (1.000)
Thalamus.Early.Mid.Fetal	0.318 (0.636)	0.860 (1.000)	1.000 (1.000)	1.000 (1.000)
Amygdala.Late.Fetal	0.902 (0.941)	0.625 (1.000)	1.000 (1.000)	1.000 (1.000)
Cerebellum.Late.Fetal	0.181 (0.473)	0.471 (1.000)	0.229 (1.000)	0.025 (1.000)
Cortex.Late.Fetal	0.123 (0.388)	0.095 (0.817)	0.025 (1.000)	1.000 (1.000)
Hippocampus.Late.Fetal	0.530 (0.821)	0.298 (1.000)	1.000 (1.000)	1.000 (1.000)
Striatum.Late.Fetal	0.300 (0.636)	0.409 (1.000)	1.000 (1.000)	1.000 (1.000)
Thalamus.Late.Fetal	0.967 (0.974)	0.859 (1.000)	0.895 (1.000)	1.000 (1.000)
Amygdala.Late.Infancy	0.876 (0.941)	0.975 (1.000)	1.000 (1.000)	1.000 (1.000)
Cerebellum.Late.Infancy	0.025 (0.135)	0.910 (1.000)	0.392 (1.000)	0.436 (1.000)
Cortex.Late.Infancy	0.621 (0.821)	1.000 (1.000)	1.000 (1.000)	1.000 (1.000)
Hippocampus.Late.Infancy	0.822 (0.913)	0.575 (1.000)	0.549 (1.000)	1.000 (1.000)
Striatum.Late.Infancy	0.680 (0.850)	0.986 (1.000)	1.000 (1.000)	1.000 (1.000)
Thalamus.Late.Infancy	0.372 (0.676)	0.836 (1.000)	1.000 (1.000)	1.000 (1.000)
Amygdala.Late.Mid.Fetal	0.131 (0.393)	0.349 (1.000)	1.000 (1.000)	1.000 (1.000)
Cerebellum.Late.Mid.Fetal	0.052 (0.222)	0.567 (1.000)	0.415 (1.000)	0.197 (1.000)
Cortex.Late.Mid.Fetal	0.007 (0.052)	0.289 (1.000)	1.000 (1.000)	1.000 (1.000)
Hippocampus.Late.Mid.Fetal	0.330 (0.639)	0.183 (1.000)	0.109 (1.000)	0.124 (1.000)
Striatum.Late.Mid.Fetal	0.393 (0.694)	0.331 (1.000)	0.246 (1.000)	0.162 (1.000)
Thalamus.Late.Mid.Fetal	0.715 (0.876)	0.467 (1.000)	0.441 (1.000)	0.886 (1.000)
Amygdala.Middle.Late.Childhood	0.910 (0.941)	1.000 (1.000)	1.000 (1.000)	1.000 (1.000)
Cerebellum.Middle.Late.Childhood	0.012 (0.079)	0.111 (0.835)	1.000 (1.000)	1.000 (1.000)
Cortex.Middle.Late.Childhood	0.587 (0.821)	0.730 (1.000)	0.549 (1.000)	1.000 (1.000)
Hippocampus.Middle.Late.Childhood	0.621 (0.821)	0.653 (1.000)	1.000 (1.000)	1.000 (1.000)
Striatum.Middle.Late.Childhood	0.774 (0.901)	0.993 (1.000)	1.000 (1.000)	1.000 (1.000)
Thalamus.Middle.Late.Childhood	0.521 (0.821)	0.994 (1.000)	1.000 (1.000)	1.000 (1.000)
Amygdala.Neotal.Early.Infancy	0.541 (0.821)	0.327 (1.000)	0.085 (1.000)	1.000 (1.000)
Cerebellum.Neotal.Early.Infancy	0.286 (0.636)	0.612 (1.000)	0.916 (1.000)	0.768 (1.000)
Cortex.Neotal.Early.Infancy	2.281e-04 (0.005)	0.770 (1.000)	0.485 (1.000)	1.000 (1.000)
Hippocampus.Neotal.Early.Infancy	0.577 (0.821)	0.359 (1.000)	1.000 (1.000)	1.000 (1.000)
Striatum.Neotal.Early.Infancy	0.095 (0.356)	0.205 (1.000)	1.000 (1.000)	1.000 (1.000)
Thalamus.Neotal.Early.Infancy	0.002 (0.021)	0.081 (0.808)	0.390 (1.000)	1.000 (1.000)
Amygdala.Young.Adulthood	0.664 (0.847)	0.778 (1.000)	1.000 (1.000)	1.000 (1.000)
Cerebellum.Young.Adulthood	0.355 (0.666)	0.774 (1.000)	1.000 (1.000)	1.000 (1.000)
Cortex.Young.Adulthood	8.368e-06 (5.021e-04)	0.054 (0.808)	1.000 (1.000)	1.000 (1.000)
Hippocampus.Young.Adulthood	0.513 (0.821)	0.644 (1.000)	0.638 (1.000)	1.000 (1.000)
Striatum.Young.Adulthood	0.138 (0.395)	0.712 (1.000)	0.197 (1.000)	1.000 (1.000)
Thalamus.Young.Adulthood	0.199 (0.498)	0.195 (1.000)	1.000 (1.000)	1.000 (1.000)

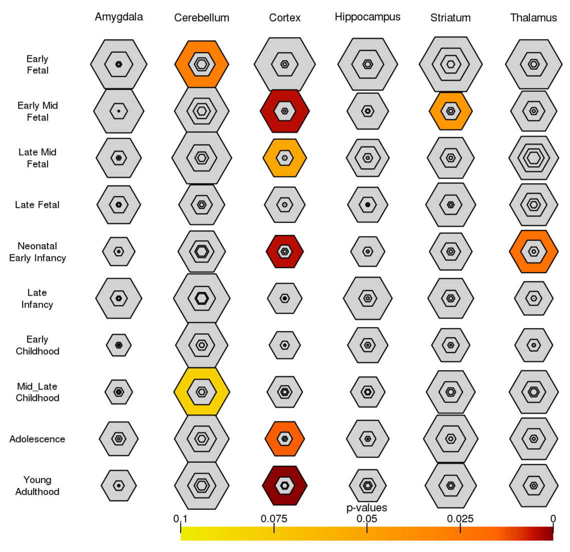

The overlapped genes for every brain region and development (pSI threshold 0.05 **) are:**
Amygdala.Adolescence : CHST6, CPNE5, GRIN2A, FAM153B, MCHR1, DYSF, KCNH3, DIO2, PCDH7, DNAJC22, MAPK3, P2RX2, GPR37, NCKIPSD, SYNJ2, PREX1, GABRA2, ABCA2, SLC7A4, OPRD1, MPL, PITPNM2, SLITRK1, MCHR2
Cerebellum.Adolescence : ANKRD34C, FLT3, GPLD1, PDZK1, KCNJ11, RTN4R, CALN1, CCDC134, LAMA5, FARP2, AMH, UNC13C, ZFYVE28, CDH23, MAP3K6, PRSS3, CRHR1, KCNC3, ABCA7, YPEL4, COL8A1, PLA2R1, TOB2, EPB41, CNTN6, RELN, FGF17, KCNIP4, ARHGAP26, ZDHHC11, RASGRP1, DHDDS, TEF, GPR63, NR4A2, PAN3, FAM153B, KCNK1, YIPF7, ARVCF, CHGB, CADPS2, CDH7, RIMS1, STARD5
Cortex.Adolescence : ANKRD34C, PI4KA, GRIN2A, FAM153B, MCHR1, KCNH3, FHL2, SULT4A1, PCDH7, KCNC3, RHEBL1, NSF, HRH2, P2RX6, BHLHE40, NCKIPSD, CX3CL1, OLFM1, RASGRP1, HCN1, SLC24A2, DAGLA, AGBL4, SLC4A10, DUSP6, RIMBP2, OPRD1, MPL, PITPNM2, SLITRK1, MCHR2, NGEF, PPP1R16B, KALRN
Hippocampus.Adolescence : CHST6, PI16, SORT1, DYSF, KCNH3, DNAJC22, CHST5, P2RX2, P2RX6, HPD, SLC39A12, CHADL, AEBP1, ABCA2, PDE6B, SLC7A4, IGSF10, OPRD1, CLCA4, CHDH, PRODH, GEM
Striatum.Adolescence : CHST6, COL9A2, DYSF, PLEK, ZDHHC11, CALN1, GPR75, SORT1, CPNE5, KLHL32, CPS1, BBX, MYO10, ETV7, STON2, DDN, DNAJC22, DLC1, GPR37, HRH2, CACNA1S, BHLHE40, OBSCN, TJP1, DRD2, FMO2, DLX6, SLC24A2, PREX1, RBMS1, CTNNA3, ABCA2, TRMT61A, OPRD1, KLB, GPR149, YIPF7, CLCA4, CMTM5, NGEF, PPP1R16B
Thalamus.Adolescence : ANKRD34C, PDCD1, FLT3, OPRM1, CPEB1, WNT3, HCN4, DHRS11, TNFAIP2, KCNH5, SAMD11, CRHR1, CLTCL1, TANC1, SLC23A2, NSF, DPP6, ADM, P2RX6, BHLHE40, HDC, CNTNAP5, SYNJ2, ADAM23, HPD, FMO2, RASGRP1, PAM, SLC24A2, MEI1, SGPP2, SEMA3E, PTPRU, VWC2, ALPK3, MAP3K6, HOMER2, PDE4A, EMB
Amygdala.Early.Childhood : CMTM5, GRIN2A, CXCL14, CLDN5, DPEP2
Cerebellum.Early.Childhood : FLT3, GPLD1, PDZK1, KCNJ11, RTN4R, CALN1, LAMA5, FARP2, AMH, UNC13C, ZFYVE28, ZNF385D, CDH23, OAS3, CRHR1, KCNC3, ABCA7, SPRY3, MUC6, YPEL4, CD160, PLA2R1, POLE, RANBP10, EPB41, CNTN6, RELN, FGF17, ARHGAP26, TOB2, SPRY2, PRSS3, DAGLA, DHDDS, PAN3, MXD4, KCNK1, YIPF7, ARVCF, ANK3, CHGB, CADPS2, CDH7, RIMS1, STARD5
Cortex.Early.Childhood : ANKRD34C, SLC39A12, CXCL14, MPL, PITPNM2, CX3CL1, HRH2, GRIN2A, KALRN, MCHR2, OLFM1, DIO2, CACNA1I, FHL2
Hippocampus.Early.Childhood : SLC39A12, PI16, CLDN5, DNAJC22, DLEC1, RASL11A, SLC27A2, HPD, CHADL, P2RX2, SORT1, CLCA4, SELE, GPR37, SERPIND1, CMTM5, EPSTI1, COL8A1, SGCZ
Striatum.Early.Childhood : DRD2, NGEF, DDN, CX3CL1, VGF, CACNA2D3, OPRD1, GPR149, DLC1, CPNE5, DLX6, HRH2, MPPE1, CMTM5, PPP1R16B
Thalamus.Early.Childhood : KCNH5, HDC, SAMD11, MGST1, PDCD1, VWC2, AIFM3, EMB, WNT3, TANC1, SLC24A2, HOMER2, ANKRD34C, ADM, RASL11A, MEI1, ADAM23
Amygdala.Early.Fetal : PCDHB13, TROAP, SCN5A, WDR62, MEX3B, NOTCH1, RRM1, RBM12B, KIF24, BEND4, ZNF518B, XYLT1, SENP1, RNF169, IGSF3, TEAD2, GABPB1, CAPN6, PIM1, DLX6, PTBP2, KIF20B, EGFR, HMGN2, EPHB2, RFWD3, SERINC2, KDM5B, GPR149, LTBP1, POLD1, IGF2BP1, COL5A1, ZNF681, CD276
Cerebellum.Early.Fetal : DHX57, POU3F2, COL4A2, TBX1, FOXP1, KDM5B, CNTNAP2, CHRNB4, AMH, PRKAR2A, KLHL5, HCN4, FBN2, ROBO3, ZNF385D, ZNF33B, SPRY3, RBM12B, SLC6A4, COL6A3, NFE2L3, BEND4, NPAS3, EPB41, RBMS1, IGSF3, CABLES2, TRIO, CELSR3, ABCC1, ADAMTS6, PRPH, POU4F2, COL5A1, KIF26B, DCC, BAZ2B, RAB42, PABPC5, PCDH17, EPHB2, KIDINS220, DOK3, DNAJB7, IGSF10, BCL11A, WNT1, PHOX2B, CNTNAP4, IGF2BP1, C1QL4, POSTN, RUNX1T1
Cortex.Early.Fetal : LMO3, BCL7A, TROAP, BCL11A, KCNH7, NEO1, DPP4, AFF3, MEX3B, FBN2, YTHDF2, NOTCH1, RRM1, ZNF33B, ZNF529, LIMCH1, CSGALNACT2, RBM12B, RNF169, PAPOLG, KIF24, ZNF518B, RCAN1, SENP1, ASPN, IGSF3, CELSR3, ADAMTS6, PTPRU, COL6A3, NR4A2, KDM5B, DCC, ANKRD45, PIM1, SLC26A2, PTBP2, KIF20B, FBN3, KIDINS220, TBR1, RFWD3, HS3ST4, DAP, SERINC2, XRN2, POLD1, MATN1, IGF2BP1, ZNF681
Hippocampus.Early.Fetal : TROAP, THBS1, KDM5B, ALDH18A1, WDR62, CHRNB4, HS2ST1, HTRA3, MEX3B, FBN2, NOTCH1, RRM1, LY6G6D, PKN2, PPP1R3B, PROKR2, KIF24, SERPIND1, SENP1, LY6G6F, COL6A3, STON2, DCC, TSHR, WNT5A, KIF20B, TEAD2, LOXL1, HMGN2, EPHB2, TBR1, RFWD3, SERINC2, XRN2, LTBP1, POLD1, PIM1, IGF2BP1, COL5A1, ZNF681
Striatum.Early.Fetal : POU3F2, TROAP, SCN5A, KCNH7, WDR62, NOTCH1, RRM1, PKN2, RBM12B, DNAH11, KIF24, CXXC4, GEM, BEND4, XYLT1, SENP1, RNF169, TEAD2, GABPB1, CAPN6, PIM1, DLX6, PTBP2, KIF20B, RAB42, HMGN2, EPHB2, RFWD3, ZBTB20, SERINC2, GPR149, POLD1, IGF2BP1, ZNF681, POLE, MPHOSPH9, CD276
Thalamus.Early.Fetal : POU3F2, WNT3, OPRM1, TEAD2, MAPT, PRKAR2A, MEX3B, HCN4, CNTNAP2, RRM1, ERC2, RBM12B, PPP1R3B, NEO1, CXXC4, BEND4, ZNF518B, SENP1, IGSF3, CABLES2, KLHL5, TUBA1A, RAB42, PABPC5, SLC26A2, PTBP2, KIF20B, OPRK1, HMGN2, EPHB2, KIDINS220, DUSP6, ZNF681, KDM5B, SDC3, IGF2BP1, LOXL1, RUNX1T1, CD276
Amygdala.Early.Mid.Fetal : POU3F2, BCL7A, TROAP, WDR62, NIPBL, AFF3, THBS1, MEX3B, FBN2, NOTCH1, RRM1, NINL, KIF24, POLE, SF3A2, IGSF3, CELSR3, PRPH, TEAD2, GTF2IRD1, GABPB1, PIM1, DLX6, KIF20B, EGFR, ZNF74, CENPJ, HMGN2, FBN3, EPHB2, VPS37B, TBR1, SERINC2, LTBP1, POLD1, IGF2BP1, DBN1, CD276
Cerebellum.Early.Mid.Fetal : TROAP, ADAMTS6, TEAD2, CHRNB4, FBN2, HTRA3, HCN4, C1QL4, COL4A2, ROBO3, SPAG17, SAMD11, SLC6A4, PPP1R3B, KIF24, NFE2L3, POLE, SERPIND1, NPAS3, LOXL1, RBMS1, COL6A3, ABCC1, RELN, PRPH, POU4F2, GTF2IRD1, SLC27A2, DCC, RAB42, PCDH17, ZNF536, EGFR, EPHB2, WNT5A, SCARF2, IGSF10, WNT1, GPR157, IGF2BP1, COL5A1, POSTN, CD276
Cortex.Early.Mid.Fetal : POU3F2, LMO3, BCL7A, MYT1L, PCDH11X, NEO1, DPP4, MAPT, TNIK, AFF3, THBS1, FBN2, YTHDF2, ZNF33B, LIMCH1, CSGALNACT2, TUBA1A, PROKR2, PAPOLG, RCAN1, IGSF3, ZNF557, TRIO, CELSR3, PTPRU, COL6A3, OBSCN, DNAH10, KDM5B, DCC, FLG, EEF2, GPRIN1, EFNA5, MEX3B, NR4A2, ZNF74, FBN3, KIDINS220, SEMA3E, TBR1, HS3ST4, DAP, SERINC2, BCL11A, XRN2, TCF20, IGF2BP1, RUNX1T1, DBN1, SH3BP2
Hippocampus.Early.Mid.Fetal : BCL7A, TROAP, THBS1, FBN2, NOTCH1, LY6G6D, SELE, SERPIND1, LOXL1, IGSF3, LY6G6F, COL6A3, POU4F2, KIF26B, DCC, TSHR, FLG, PIM1, MEX3B, EGFR, ZNF74, TBR1, ZBTB20, SERINC2, KDM5B, LTBP1, PROKR2, IGF2BP1, COL5A1, DBN1, CD276
Striatum.Early.Mid.Fetal : PCDHB13, PDCD1, FOXP1, BCL11A, SCN5A, CNTNAP5, CHRNB4, CNTNAP2, RIMBP3, NINL, KDM5B, CXXC4, LMO4, IGSF3, TRIO, CELSR3, HDC, RELN, GTF2IRD1, SORCS3, KIF26B, DCC, ANKRD45, DRD2, DLX6, PCDH17, ZNF74, FBN3, KIDINS220, MYT1L, WNT1, GPR149, TCF20, IGF2BP1, RUNX1T1, SH3BP2
Thalamus.Early.Mid.Fetal : KLHL5, OPRM1, CHRNB4, MAPT, PRKAR2A, WNT3, CNTNAP2, CHRNA7, ERC2, TUBA1A, PPP1R3B, CXXC4, OPRK1, CNTN6, CABLES2, HDC, CACNA2D3, PABPC5, MDGA2, RUNX1T1, PCDH17, FLG, IGSF10, IGSF3, PCDHAC1, CDH7, HOMER2, NXPH1, SDC3
Amygdala.Late.Fetal : POU3F2, LY6G6D, TBR1, PI16, LY6G6F, SERINC2, PLEK, FPR1, SLC10A4, GCG, NOTCH1, LTBP1, PRPH, THBS1, CAPN6, LAPTM5, GEM, MPO
Cerebellum.Late.Fetal : THBS1, OPRM1, FPR1, MCHR1, ADAMTS6, MPO, CRHR1, ITGB6, NEO1, IL17RB, NPAS3, EPB41, RELN, PRPH, POU4F2, KCNIP4, CAPN6, PABPC5, PLEKHH2, EGFR, EPHB2, FLG, WNT5A, CTNNA3, PAN3, CSMD3, COL5A1, ANK3, CADPS2, CDH7, STARD5
Cortex.Late.Fetal : POU3F2, LMO3, GPRIN1, DPP4, PCDHAC1, AFF3, THBS1, TNFAIP2, MPO, TUBA1A, NEO1, MACROD1, CXXC4, NFE2L3, LMO4, OPRK1, PTBP2, NR4A2, TBR1, WNT10B, TM6SF1, PROKR2, WWC1, MCHR2, POSTN, PCDH11X
Hippocampus.Late.Fetal : SEMA3E, PRPH, SIGLEC14, SLC23A1, PLEK, TSHR, TUBA1A, FPR2, FPR1, RAB42, LTBP1, PROKR2, PI16, SPAG17, C1QL4, EGFR, LAPTM5, C10ORF67, FSIP1, MPO
Striatum.Late.Fetal : PCDHB13, PDCD1, TROAP, FOXP1, CNTNAP2, SCN5A, WDR62, TNFRSF13C, CPNE5, UBAP2, C1QL4, NOTCH1, MPO, PROKR2, ZNF837, KIF24, CXXC4, LMO4, RRM1, CNTNAP5, SORCS3, RAB42, DRD2, DLX6, GABPB1, FBN3, CHRNB4, RFWD3, SCARF2, KLB, GPR149, POLD1, SLC29A3, PCDH11X
Thalamus.Late.Fetal : HDC, RHEBL1, WNT10B, B3GNT4, CACNA2D3, LAPTM5, PLEK, FPR1, TUBA1B, OLFM1, SYT17, UCHL1, STARD3NL, CSDC2, SLC29A3, C1QL4, NXPH1, CNTN6, TUBA1A, MPO
Amygdala.Late.Infancy : LY6G6D, ZNF577, ZNF799, KCNMB3, CLDN5, CD302, VRK2, CXCL14, AIFM3, PHF14, FPR1, DPEP2, APOM, RPS21, NDUFB1, FKBP11, IFIH1, CMTM5, CBR3, EPSTI1, ASPN
Cerebellum.Late.Infancy : CDH23, FLT3, RHEBL1, PDZK1, NDUFB1, KCNJ11, OPRM1, RTN4R, TNFRSF13C, LAMA5, FARP2, CDH7, AMH, UNC13C, CENPT, ZNF385D, DGCR8, DCAF4L1, ZNF577, ADRM1, CRHR1, KCNC3, ABCA7, MUC6, GPLD1, PLA2R1, RANBP10, TOB2, EPB41, CNTN6, LY6G6F, NUDT17, RELN, LY6G6D, VPS13A, ZDHHC11, ST20, PRSS3, FAM153B, AQP5, NR4A2, CCDC66, MXD4, KCNK1, YIPF7, RPS21, CHGB, CADPS2, IP6K2, NOP14, RIMS1, STARD5
Cortex.Late.Infancy : IFIH1, FKBP11, RHEBL1, KCNMB3, MTIF3, UNC13C, GTF3A, CXCL14, ST20, NDUFB1, MCHR2, CBR3, SLC39A12, EPSTI1
Hippocampus.Late.Infancy : NDUFB1, CDK2AP1, SLC39A12, SLC43A1, ASPN, ZNF577, MTIF3, P2RX2, IL17RB, FSIP1, KCNMB3, CLDN5, OXNAD1, CXCL14, ST20, POSTN, DPEP2, CBR3, IFIH1, AIFM3, DNAJC15, RPS21, CMTM5, EPSTI1, APOM
Striatum.Late.Infancy : NDUFA4, CD302, NDUFB1, PI16, MRPL40, LY6G6D, POLN, MTIF3, POLR2I, RBX1, KCNMB3, CLDN5, CXCL14, ST20, PNKD, DPEP2, AQP5, IFIH1, NDUFA13, PHF14, RPS21, CMTM5, CBR3, APOM
Thalamus.Late.Infancy : HDC, AIFM3, SAMD11, MGST1, PDCD1, CLTCL1, VWC2, PDGFRL, HPD, SLC39A12, NDUFB1, CDKN1A, KCNMB3, FHIT, VGF, HOMER2, ADM, CNTN6, AQP5, MEI1
Amygdala.Late.Mid.Fetal : STK38, TROAP, CHRNB4, THBS1, DPP4, SPAG17, ZNF33B, LMBR1L, NINL, ZNF83, MYO19, BEND4, ABCC1, STARD9, PRPH, FGF17, DNAH7, FLG, ZNF382, GPRIN1, LY6G5B, CENPJ, FBN3, NR4A2, TBR1, DNAJB7, IGSF10, PROKR2, TRIO, POSTN, PCDH11X
Cerebellum.Late.Mid.Fetal : STARD9, KCTD20, TROAP, MCHR1, NEO1, SCN5A, ADAMTS6, WDR62, CHRNB4, KIF20B, SYNJ2, THBS1, PREX1, FBN2, WBP2NL, ROBO3, ZNF33B, NINL, PPP1R3B, RFWD3, KIF24, NFE2L3, XYLT1, POLE, LOXL1, RBMS1, LIX1L, TEAD2, RELN, PRPH, POU4F2, OBSCN, KCNIP4, TNFRSF13C, DCC, BAZ2B, MPHOSPH9, PABPC5, ZNF169, PCDH17, PLCH1, EGFR, CENPJ, FBN3, EPHB2, FLG, WNT5A, PLEKHH2, PAN3, SERINC2, ARVCF, IGF2BP1, COL5A1, COL4A2, CDH7
Cortex.Late.Mid.Fetal : POU3F2, BCL7A, DPP4, MAPT, AFF3, THBS1, HCN4, TNFAIP2, LY6G6D, NINL, NEO1, MACROD1, CXXC4, NFE2L3, CTRL, LMO4, TRIO, FGF17, OBSCN, DNAH10, ZNF169, ZNF382, GPRIN1, MEX3B, FBN3, TBR1, MYT1L, TM6SF1, PROKR2, WWC1, PCDH11X, MCHR2, RUNX1T1, LY6G6F, CACNA1I, SH3BP2
Hippocampus.Late.Mid.Fetal : DLEC1, DNAH7, DYNC2H1, THBS1, SPAG17, C10ORF67, DNAH11, SERPIND1, FSIP1, DNAH10, KIF26B, TSHR, FLG, DNAH3, FBN3, SEMA3E, ZBTB20, IGSF10, TSNAXIP1, LTBP1, PROKR2, CASP8, BNC1, TMEM67, DNAH5
Striatum.Late.Mid.Fetal : PDCD1, TROAP, FOXP1, WDR62, RGS12, CPNE5, C1QL4, SF3A2, DGCR8, RIMBP3, NINL, ZNF837, LMO4, FGF17, SORCS3, MPPE1, DRD2, DLX6, RAB42, FBN3, PCDH17, SCARF2, BCL11A, WNT1, GPR149, TCF20, NGEF, SH3BP2, GSC2
Thalamus.Late.Mid.Fetal : DNAH1, DLEC1, SLC23A1, DNAH7, DYNC2H1, C1QL4, SPAG17, C10ORF67, DNAH11, KIF24, NEGR1, FSIP1, HDC, ADAMTS6, GABRG1, DNAH10, LRRC43, PCDH12, FMO2, DNAH3, COL8A1, ODF2L, TSNAXIP1, ALPK3, CASP8, TMEM67, SDC3, DNAH5
Amygdala.Middle.Late.Childhood : SLC7A4, MAPK3, P2RX2, CLCA4, MCHR2, SGCZ
Cerebellum.Middle.Late.Childhood : FLT3, GPLD1, KCNJ11, ASH1L, ZDHHC11, CALN1, CCDC134, LAMA5, PDZK1, CDH7, AMH, UNC13C, ZFYVE28, ZNF385D, CDH23, MAP3K6, PHC3, CRHR1, KCNC3, SPRY3, TMED3, YPEL4, COL8A1, PLA2R1, POLE, RANBP10, EPB41, SGPP2, INO80D, RELN, OBSCN, ARHGAP26, TOB2, WWOX, SPRY2, RASGRP1, HCN1, ATN1, PRSS3, DHDDS, TEF, NR4A2, PAN3, FAM153B, KCNK1, RERE, ARVCF, ANK3, CHGB, CADPS2, NOP14, RIMS1, STARD5
Cortex.Middle.Late.Childhood : AGBL4, SULT4A1, RHEBL1, VAMP7, MDH1, HCN1, MPL, GRIN2A, MCHR1, MCHR2, OLFM1, NGEF, ARHGEF3, P2RX6, FHL2
Hippocampus.Middle.Late.Childhood : SLC4A10, NDUFA4, CTNNA3, SLC27A2, MDH1, HPD, RASL11A, NSF, P2RX2, SORT1, CLCA4, GPR37, IMPA1, SLC39A12
Striatum.Middle.Late.Childhood : PDCD1, NDUFA4, WBP2NL, PDGFRL, PLEK, FPR1, CPNE5, CPS1, FHIT, NUDT17, RASL11A, IL17RB, CACNA1S, MGST1, BST1, DRD2, AQP5, TEF, NDUFA13, GPR75, GPR149, YIPF7, CLCA4, CBR3, NGEF, C10ORF67
Thalamus.Middle.Late.Childhood : ANKRD34C, PDCD1, MDH1, ZDHHC11, SLC10A4, CPEB1, WNT3, OAS3, KCNH5, NAPSA, CLTCL1, RASL11A, ADAM23, NSF, OPRK1, ADM, TLK1, MGST1, FPR1, DRD2, PAM, MEI1, SGPP2, SEMA3E, CACNA1S, XPNPEP3, GFM2, HOMER2, NXPH1, EMB
Amygdala.Neotal.Early.Infancy : SCARF2, DDN, MUC6, SCN5A, WNT1, TSHR, DIO2, GP1BB, KCNH3, CCDC85A, SLC23A1, CACNA1I, SGCZ
Cerebellum.Neotal.Early.Infancy : FLT3, RTN4R, TNFRSF13C, LAMA5, PDZK1, AMH, ZNF385D, ASPN, CDH23, CRHR1, KCNC3, SLC6A4, NUP85, SERPIND1, CNTN6, COL6A3, RELN, LY6G6D, KCNIP4, ARHGAP26, CAPN6, GPR63, WNT5A, KCNK1, BNC1, COL5A1, CHGB, EPSTI1, CADPS2, CDH7, STARD5
Cortex.Neotal.Early.Infancy : SLC10A4, GRIN2A, KALRN, THBS1, KCNH3, UNC13C, TM6SF1, ARHGEF3, ASPN, ERC2, DDN, VGF, RHEBL1, LINGO1, GP1BB, HRH2, SULT4A1, ADCY2, SHANK2, CX3CL1, OLFM1, SGCZ, PLCH1, HS3ST4, RIMBP2, WNT10B, PITPNM2, SYT17, PROKR2, WWC1, CCDC85A, MCHR2, CACNA1I
Hippocampus.Neotal.Early.Infancy : ASPN, COL6A3, TRAF3, MUC6, CXCL14, P2RX2, SYT17, FMO2, TSHR, SERPIND1, NPAS3, GAL3ST2, SLC23A1, DIO2
Striatum.Neotal.Early.Infancy : PDCD1, SLC10A4, SLC23A1, ASPN, HTRA3, GAL3ST2, ETS1, ETV7, FN1, DDN, DLC1, GP1BB, HRH2, CACNA1S, FGF17, LRRC43, PCDH12, MPPE1, DRD2, DLX6, COL8A1, SGCZ, CX3CL1, OPRD1, WNT1, GPR149, PRR7, NGEF, PPP1R16B
Thalamus.Neotal.Early.Infancy : ANKRD34C, PDCD1, MCHR1, PDGFRL, UCHL1, CDKN1A, WNT3, HCN4, FBXO10, CHRNA7, KCNH5, NRCAM, SAMD11, VGF, TANC1, SLC23A2, OLFM1, OPRK1, CNTN6, SLC45A1, HDC, CNTNAP5, OPRM1, TLK1, DGKQ, KCNIP4, NLGN4X, CX3CL1, ADAM23, SLC24A2, MEI1, SGPP2, GPR63, WNT5A, VWC2, ABCB9, ALPK3, TLN2, CCDC85A, RIMS1, SLC29A3, CLTCL1, HOMER2, IMPA1, NXPH1, EMB
Amygdala.Young.Adulthood : PI16, CTNNA3, ABCA2, PDE6B, MAPK3, DYSF, SYNJ2, P2RX2, FMO2, CLCA4, MATN1, GPR37, CPS1, MCHR2
Cerebellum.Young.Adulthood : ANKRD34C, FLT3, GPLD1, KCNJ11, RTN4R, CALN1, CCDC134, LAMA5, PDZK1, UNC13C, ZFYVE28, CDH23, PRSS3, CRHR1, KCNC3, ABCA7, SPRY3, YPEL4, NUP85, PLA2R1, SLC26A2, RELN, FGF17, OBSCN, ARHGAP26, MAP3K6, RASGRP1, FAM153B, DHDDS, TEF, VWC2, MXD4, KCNK1, ARVCF, CHGB, CADPS2, CDH7, RIMS1, STARD5
Cortex.Young.Adulthood : DNAH1, RHEBL1, CAMK1D, CACNA1D, PI4KA, SORT1, GRIN2A, CPS1, MCHR1, KCNH3, NAT8L, SULT4A1, PCDH7, KCNC3, YPEL4, NSF, HRH2, FHL2, P2RX6, BHLHE40, NCKIPSD, SYNJ2, CSMD1, ARHGAP26, SLC4A10, OLFM1, NGEF, HCN1, SLC24A2, FAM153B, KLHL38, PDE4A, AGBL4, PREPL, TBR1, RIMBP2, OPRD1, WNT1, MPL, PITPNM2, CPEB1, SLITRK1, TLN2, MCHR2, CADPS2, CACNA1I, PPP1R16B, KALRN
Hippocampus.Young.Adulthood : SLC39A12, SYNJ2, CTNNA3, ABCA2, SLC27A2, IGSF10, HPD, FAM153B, P2RX2, SORT1, CPS1, DYSF, KCNH3, PRODH, COL8A1, P2RX6, CPEB1
Striatum.Young.Adulthood : CHST6, DNAH1, COL9A2, PDGFRL, PLEK, ZDHHC11, SLC10A4, WNT1, CPNE5, KLHL32, CPS1, DYSF, ETV7, STON2, POLN, DNAJC22, ZNF83, MUC6, DLC1, GPR37, STARD9, SYNJ2, OBSCN, TJP1, NAALAD2, DRD2, FMO2, CHADL, CTNNA3, ABCA2, PDE6B, KLB, FAM153B, GPR149, CLCA4, CHDH, CMTM5, NGEF, PPP1R16B
Thalamus.Young.Adulthood : ANKRD34C, FLT3, OPRM1, CPEB1, WNT3, DYSF, TLK1, TNFAIP2, KCNH5, SAMD11, NIPA1, CRHR1, CLTCL1, TANC1, ADM, P2RX6, BHLHE40, SYNJ2, HCN4, ADAM23, HPD, FMO2, SLC24A2, MEI1, SGPP2, CTNNA3, ABCA2, VWC2, ALKBH6, MAP3K6, CNTNAP4, PDE4A, RASL11A, EMB

The overlapped genes for every brain region and development (pSI threshold 0.01 **) are:**
Amygdala.Adolescence : ABCA2
Cerebellum.Adolescence : FLT3, CADPS2, KCNC3, RELN, ARHGAP26, KCNK1, MAP3K6, PDZK1, CHGB, ZFYVE28
Cortex.Adolescence : OPRD1, MPL, GRIN2A, MCHR1, MCHR2, FHL2
Hippocampus.Adolescence : KCNH3, IGSF10, HPD, P2RX2
Striatum.Adolescence : DLC1, KLB, DRD2, GPR37, DYSF, SLC24A2, PPP1R16B
Thalamus.Adolescence : ANKRD34C, PDCD1, SAMD11, KCNH5, EMB, WNT3, SLC24A2, MEI1
Amygdala.Early.Childhood : none
Cerebellum.Early.Childhood : CDH23, FLT3, STARD5, CRHR1, KCNC3, CALN1, KCNK1, CADPS2, PDZK1, AMH, CHGB, CD160, PLA2R1
Cortex.Early.Childhood : GRIN2A
Hippocampus.Early.Childhood : SLC39A12, SELE, HPD
Striatum.Early.Childhood : DRD2, HRH2, NGEF, OPRD1
Thalamus.Early.Childhood : KCNH5, SAMD11
Amygdala.Early.Fetal : TROAP, BEND4, DLX6, IGF2BP1, LTBP1
Cerebellum.Early.Fetal : EPHB2, PCDH17, PRPH, POU4F2, SLC6A4, PHOX2B, CHRNB4, POSTN, C1QL4, HCN4, NPAS3, ROBO3, RBMS1, DCC
Cortex.Early.Fetal : LMO3, TBR1, NR4A2, SERINC2, DCC, BCL11A, AFF3, FBN2, KIF20B, IGSF3, IGF2BP1
Hippocampus.Early.Fetal : STON2, SERINC2, TSHR, LTBP1, PROKR2, IGF2BP1, FBN2, LOXL1, TEAD2
Striatum.Early.Fetal : TROAP, WDR62, CAPN6, RFWD3, KIF24, POLD1, PIM1, BEND4, IGF2BP1, KIF20B, RAB42, NOTCH1, RRM1
Thalamus.Early.Fetal : POU3F2, EPHB2, RAB42, CXXC4, IGF2BP1, WNT3, MEX3B, RUNX1T1, OPRK1
Amygdala.Early.Mid.Fetal : EGFR, TROAP
Cerebellum.Early.Mid.Fetal : COL6A3, ADAMTS6, PRPH, POU4F2, SLC6A4, C1QL4, WNT1, POSTN, GPR157, COL5A1, FBN2
Cortex.Early.Mid.Fetal : POU3F2, NR4A2, LIMCH1, TBR1, LMO3, BCL11A, DPP4, BCL7A, AFF3, THBS1, MEX3B, IGSF3
Hippocampus.Early.Mid.Fetal : LTBP1, PROKR2, POU4F2
Striatum.Early.Mid.Fetal : RIMBP3, FOXP1, DCC, GPR149, DLX6, RUNX1T1
Thalamus.Early.Mid.Fetal : HDC, OPRK1, OPRM1, SDC3
Amygdala.Late.Fetal : CAPN6, MPO, LAPTM5, GCG
Cerebellum.Late.Fetal : RELN, WNT5A, POU4F2, ITGB6, CAPN6, CSMD3
Cortex.Late.Fetal : TBR1, PROKR2, THBS1, GPRIN1, LMO4, PCDH11X
Hippocampus.Late.Fetal : PI16, MPO, FPR2, SIGLEC14, TSHR
Striatum.Late.Fetal : PDCD1, TROAP, FOXP1, CHRNB4, DLX6, PCDH11X, MPO
Thalamus.Late.Fetal : WNT10B, B3GNT4, CACNA2D3, C1QL4, NXPH1, MPO
Amygdala.Late.Infancy : EPSTI1
Cerebellum.Late.Infancy : DCAF4L1, CDH23, MUC6, PDZK1, AMH, STARD5
Cortex.Late.Infancy : none
Hippocampus.Late.Infancy : POSTN, IFIH1, KCNMB3, P2RX2
Striatum.Late.Infancy : DPEP2
Thalamus.Late.Infancy : PDCD1, SAMD11
Amygdala.Late.Mid.Fetal : POSTN, IGSF10, PROKR2, PRPH
Cerebellum.Late.Mid.Fetal : EPHB2, ADAMTS6, PRPH, POU4F2, SCN5A, STARD9, FLG, WNT5A, POLE, COL5A1, EGFR
Cortex.Late.Mid.Fetal : DPP4, CXXC4, FBN3, THBS1, DNAH10
Hippocampus.Late.Mid.Fetal : DLEC1, SERPIND1, TSNAXIP1, TSHR, DNAH7, PROKR2, BNC1, TMEM67, DNAH3, SPAG17, DNAH5
Striatum.Late.Mid.Fetal : PDCD1, RIMBP3, FOXP1, WNT1, DLX6, FBN3, GSC2
Thalamus.Late.Mid.Fetal : HDC, GABRG1, DNAH10, DLEC1, TSNAXIP1, DNAH11, SLC23A1, DNAH7, C1QL4, TMEM67, DNAH3, SPAG17, DNAH5
Amygdala.Middle.Late.Childhood : none
Cerebellum.Middle.Late.Childhood : CDH23, FLT3, PRSS3, YPEL4, RELN, ARHGAP26, CALN1, CADPS2, FAM153B, PDZK1, CHGB, UNC13C, ZFYVE28, CDH7, STARD5
Cortex.Middle.Late.Childhood : MPL, MCHR2
Hippocampus.Middle.Late.Childhood : HPD, SLC27A2
Striatum.Middle.Late.Childhood : CBR3, MGST1
Thalamus.Middle.Late.Childhood : ADM, SLC10A4
Amygdala.Neotal.Early.Infancy : DIO2, SLC23A1
Cerebellum.Neotal.Early.Infancy : COL6A3, KCNC3, SLC6A4, KCNK1, BNC1, CAPN6, ASPN
Cortex.Neotal.Early.Infancy : CX3CL1, MCHR2
Hippocampus.Neotal.Early.Infancy : DIO2, SLC23A1
Striatum.Neotal.Early.Infancy : PDCD1, DDN, SLC23A1, DRD2, ETV7, NGEF, ASPN
Thalamus.Neotal.Early.Infancy : ANKRD34C, KCNH5, SAMD11, HDC, VGF, VWC2, ADAM23, ALPK3, CCDC85A, MEI1, HOMER2, CHRNA7, EMB
Amygdala.Young.Adulthood : CLCA4
Cerebellum.Young.Adulthood : FLT3, CADPS2, FGF17, YPEL4, KCNK1, PDZK1, ZFYVE28, STARD5
Cortex.Young.Adulthood : NCKIPSD, OPRD1, PI4KA, FAM153B, GRIN2A, HCN1, KLHL38, P2RX6, MCHR2
Hippocampus.Young.Adulthood : SLC39A12, HPD, P2RX2
Striatum.Young.Adulthood : PDE6B, FMO2, CLCA4, GPR37, CPS1, DYSF
Thalamus.Young.Adulthood : ANKRD34C, KCNH5, SYNJ2, CTNNA3, NIPA1, TANC1, SLC24A2, MEI1

The overlapped genes for every brain region and development (pSI threshold 0.001 **) are:**
Amygdala.Adolescence : none
Cerebellum.Adolescence : CADPS2
Cortex.Adolescence : none
Hippocampus.Adolescence : none
Striatum.Adolescence : none
Thalamus.Adolescence : ANKRD34C
Amygdala.Early.Childhood : none
Cerebellum.Early.Childhood : CDH23, PDZK1
Cortex.Early.Childhood : none
Hippocampus.Early.Childhood : none
Striatum.Early.Childhood : none
Thalamus.Early.Childhood : none
Amygdala.Early.Fetal : none
Cerebellum.Early.Fetal : C1QL4, PHOX2B
Cortex.Early.Fetal : TBR1
Hippocampus.Early.Fetal : none
Striatum.Early.Fetal : TROAP, IGF2BP1, CAPN6
Thalamus.Early.Fetal : RUNX1T1, OPRK1
Amygdala.Early.Mid.Fetal : none
Cerebellum.Early.Mid.Fetal : POSTN, POU4F2
Cortex.Early.Mid.Fetal : TBR1
Hippocampus.Early.Mid.Fetal : none
Striatum.Early.Mid.Fetal : FOXP1
Thalamus.Early.Mid.Fetal : none
Amygdala.Late.Fetal : none
Cerebellum.Late.Fetal : POU4F2, ITGB6
Cortex.Late.Fetal : PROKR2, THBS1
Hippocampus.Late.Fetal : none
Striatum.Late.Fetal : none
Thalamus.Late.Fetal : MPO
Amygdala.Late.Infancy : none
Cerebellum.Late.Infancy : CDH23, STARD5, DCAF4L1
Cortex.Late.Infancy : none
Hippocampus.Late.Infancy : P2RX2
Striatum.Late.Infancy : none
Thalamus.Late.Infancy : none
Amygdala.Late.Mid.Fetal : none
Cerebellum.Late.Mid.Fetal : ADAMTS6, PRPH, POU4F2
Cortex.Late.Mid.Fetal : none
Hippocampus.Late.Mid.Fetal : DNAH3, BNC1, SPAG17
Striatum.Late.Mid.Fetal : RIMBP3, GSC2
Thalamus.Late.Mid.Fetal : HDC, DLEC1, SLC23A1, DNAH3, SPAG17, DNAH5
Amygdala.Middle.Late.Childhood : none
Cerebellum.Middle.Late.Childhood : none
Cortex.Middle.Late.Childhood : MCHR2
Hippocampus.Middle.Late.Childhood : none
Striatum.Middle.Late.Childhood : none
Thalamus.Middle.Late.Childhood : none
Amygdala.Neotal.Early.Infancy : SLC23A1
Cerebellum.Neotal.Early.Infancy : CAPN6
Cortex.Neotal.Early.Infancy : MCHR2
Hippocampus.Neotal.Early.Infancy : none
Striatum.Neotal.Early.Infancy : none
Thalamus.Neotal.Early.Infancy : VGF, MEI1
Amygdala.Young.Adulthood : none
Cerebellum.Young.Adulthood : none
Cortex.Young.Adulthood : none
Hippocampus.Young.Adulthood : HPD
Striatum.Young.Adulthood : CPS1, DYSF
Thalamus.Young.Adulthood : none

The overlapped genes for every brain region and development (pSI threshold 0.0001 **) are:**
Amygdala.Adolescence : none
Cerebellum.Adolescence : none
Cortex.Adolescence : none
Hippocampus.Adolescence : none
Striatum.Adolescence : none
Thalamus.Adolescence : none
Amygdala.Early.Childhood : none
Cerebellum.Early.Childhood : PDZK1
Cortex.Early.Childhood : none
Hippocampus.Early.Childhood : none
Striatum.Early.Childhood : none
Thalamus.Early.Childhood : none
Amygdala.Early.Fetal : none
Cerebellum.Early.Fetal : PHOX2B
Cortex.Early.Fetal : none
Hippocampus.Early.Fetal : none
Striatum.Early.Fetal : IGF2BP1, CAPN6
Thalamus.Early.Fetal : none
Amygdala.Early.Mid.Fetal : none
Cerebellum.Early.Mid.Fetal : none
Cortex.Early.Mid.Fetal : none
Hippocampus.Early.Mid.Fetal : none
Striatum.Early.Mid.Fetal : none
Thalamus.Early.Mid.Fetal : none
Amygdala.Late.Fetal : none
Cerebellum.Late.Fetal : ITGB6, POU4F2
Cortex.Late.Fetal : none
Hippocampus.Late.Fetal : none
Striatum.Late.Fetal : none
Thalamus.Late.Fetal : none
Amygdala.Late.Infancy : none
Cerebellum.Late.Infancy : DCAF4L1, STARD5
Cortex.Late.Infancy : none
Hippocampus.Late.Infancy : none
Striatum.Late.Infancy : none
Thalamus.Late.Infancy : none
Amygdala.Late.Mid.Fetal : none
Cerebellum.Late.Mid.Fetal : ADAMTS6, PRPH
Cortex.Late.Mid.Fetal : none
Hippocampus.Late.Mid.Fetal : BNC1
Striatum.Late.Mid.Fetal : GSC2
Thalamus.Late.Mid.Fetal : DNAH3
Amygdala.Middle.Late.Childhood : none
Cerebellum.Middle.Late.Childhood : none
Cortex.Middle.Late.Childhood : none
Hippocampus.Middle.Late.Childhood : none
Striatum.Middle.Late.Childhood : none
Thalamus.Middle.Late.Childhood : none
Amygdala.Neotal.Early.Infancy : none
Cerebellum.Neotal.Early.Infancy : CAPN6
Cortex.Neotal.Early.Infancy : none
Hippocampus.Neotal.Early.Infancy : none
Striatum.Neotal.Early.Infancy : none
Thalamus.Neotal.Early.Infancy : none
Amygdala.Young.Adulthood : none
Cerebellum.Young.Adulthood : none
Cortex.Young.Adulthood : none
Hippocampus.Young.Adulthood : none
Striatum.Young.Adulthood : none
Thalamus.Young.Adulthood : none
