# Supplementary material for: Molecular Landscape of Tourette’s Disorder
Source: Int J Mol Sci. 2023 Jan 11;24(2):1428. doi: 10.3390/ijms24021428 (PMC9865021; doi:10.3390/ijms24021428)
Supplement: Supplementary file 1 [file supplementary File S5.pdf › supplementary File S5.pdf]

Of the inputed 951 genes,
740 genes exist in the brain cell type expression dataset, 211 genes do not, which are:
PGAP1,SYTL5,ACTG1,EPB41L4B,GUCY1A2,LDLRAD4,HIPK4,TOMM70,NAALADL1,CERS6,MAP10,C1ORF115,RXFP1,FSTL4,GRK5,DGCR5,SCN2A,ELAPOR1,LMBRD2,TMEM178A,FADS6,DRD5,BICDL1,DIAPH2,DLX6-AS1,SIMC1,TDRD6,FAAP20,KLHL2,SNU13,ATP2B3,CYP4X1,CENPS,ADCY5,KCNMB3,TLNRD1,TMEM241,HCN4,GLT1D1,SRSF12,PAK5,C11ORF87,NYAP1,SH3BP1,CCDC88C,KCNG3,PIANP,RNF212,FAM13B,KIAA1217,MC4R,SPTSSB,ZNF169,MYCL,SRSF2,PCDHAC2,PCDHAC1,LINC00523,GRM4,MFSD4A,CDIN1,ATP5IF1,KLHL34,ARHGEF33,TINCR,EIPR1,DNAJC30,ASIC2,PCDHA10,PCDHA11,PHF24,PCDHA13,PTPN7,PCNX2,KCNH4,HACD2,ADGRB3,CEP170B,RBFOX1,MAP7D2,HARS1,RIPOR2,LRATD1,TOMM20,EMC9,TMEM196,PNMA8A,SOGA3,GARS1,CBARP,TRERF1,C5ORF22,CYRIA,PPP6R1,TLE5,RFPL1,TENM2,TIGD3,UTP4,FABP3,CHAT,EID2B,CHMP3,BCAS4,PCDHA12,NIBAN2,CARNMT1,STRIP2,NLRP1,SLC10A4,SKIDA1,TMEM200B,AP2M1,NEURL1,C10ORF88,RAPGEFL1,SGCZ,RIMS4,ERICH5,ERICH3,RIMS1,ADGRB2,KIAA0513,SPATS2,RIMBP2,LRRC8B,OBSCN-AS1,EFNA3,ANKRD34C,KCNH6,CERT1,AKR1B1,RBFOX2,FAXC,C20ORF27,LRRC32,LMO3,SOWAHA,BABAM1,BABAM2,FAM136A,MYL6B,TUBGCP4,CDC40,FOXO6,PCDHA9,SLC2A11,ANKH,PCDHA8,PRKN,FAM220A,NFYC,RTL10,ATP5F1B,GPR55,ATP5MC1,ATP5MC3,DRD1,MICU3,CRACDL,DIAPH1,RNF175,CIART,KAZN,SIAH3,YWHAH,GRIN2A,NRG1,C12ORF40,MPPE1,OGDHL,ABHD12B,ZNF540,UQCC1,SNURF,PIGL,CHRM2,PRKCG,DMTN,YARS1,FAM216A,SNRPN,EPHA10,LINC00242,ZNF804A,SHANK2,BMERB1,PCDHA2,PCDHA3,PCDHA1,PCDHA6,PCDHA7,PCDHA4,PCDHA5,DOP1B,PIP4P1,PCP4,DRD2,B3GLCT,OAZ1,CACNG8,NUDT11,MAP3K15,FAR2,PTPRO,RELCH,EIF4A2,CACNG4,ZNF467,GPI,LZTS1

Interpretation of CSEA results table:
The first row lists the pSI thresholds, and the first column lists the brain cell types
The values in the tables are the Fisher's Exact p-values, followed by the Benjamini-Hochberg (BH) corrected values in parentheses.
Click the linked p-values to see the overlapped genes for the corresponding brain cell types and thresholds.

Brain Cell Types and P-Values	0.05	0.01	0.001	0.0001
RetR.Rods	0.999 (1.000)	1.000 (1.000)	0.996 (1.000)	1.000 (1.000)
Hyp	0.472 (0.787)	0.851 (1.000)	0.758 (1.000)	1.000 (1.000)
Hyp.Hcrt	0.093 (0.217)	0.041 (0.204)	0.461 (1.000)	0.426 (1.000)
BF	2.636e-05 (1.004e-04)	0.223 (0.547)	1.000 (1.000)	1.000 (1.000)
BF.Chat	0.002 (0.006)	0.001 (0.009)	0.007 (0.057)	9.593e-04 (0.017)
BS	1.000 (1.000)	1.000 (1.000)	1.000 (1.000)	1.000 (1.000)
BS.Chat	0.655 (1.000)	0.911 (1.000)	0.981 (1.000)	1.000 (1.000)
BS.Slc6a4	0.907 (1.000)	0.822 (1.000)	1.000 (1.000)	1.000 (1.000)
Cb	0.999 (1.000)	0.999 (1.000)	1.000 (1.000)	1.000 (1.000)
Cb.Septin4	1.000 (1.000)	1.000 (1.000)	0.998 (1.000)	1.000 (1.000)
Cb.Pcp2	0.108 (0.237)	0.227 (0.547)	0.944 (1.000)	0.865 (1.000)
Cb.Neurod1	0.369 (0.679)	0.982 (1.000)	1.000 (1.000)	1.000 (1.000)
Cb.Lypd6	0.011 (0.030)	0.120 (0.396)	0.526 (1.000)	0.878 (1.000)
Cb.Grp	0.985 (1.000)	0.697 (1.000)	0.365 (1.000)	0.713 (1.000)
Cb.Grm2	0.002 (0.006)	0.134 (0.396)	0.863 (1.000)	1.000 (1.000)
Cb.Fthfd	1.000 (1.000)	1.000 (1.000)	0.990 (1.000)	1.000 (1.000)
Cb.Cnp	1.000 (1.000)	1.000 (1.000)	1.000 (1.000)	1.000 (1.000)
Cpu	1.377e-20 (1.606e-19)	3.445e-06 (4.019e-05)	0.365 (1.000)	1.000 (1.000)
Cpu.D2	2.767e-30 (9.683e-29)	2.068e-13 (3.619e-12)	5.163e-08 (1.807e-06)	5.872e-08 (2.055e-06)
Cpu.D1	5.075e-27 (8.881e-26)	6.296e-19 (2.204e-17)	6.595e-06 (1.154e-04)	0.040 (0.323)
Cpu.Chat	1.071e-06 (6.250e-06)	4.434e-05 (3.880e-04)	6.626e-04 (0.008)	0.002 (0.018)
Ctx	9.008e-09 (6.305e-08)	0.051 (0.223)	1.000 (1.000)	1.000 (1.000)
Ctx.Pdgfrajd340	1.000 (1.000)	1.000 (1.000)	0.997 (1.000)	1.000 (1.000)
Ctx.Etv1_ts88	2.869e-05 (1.004e-04)	0.991 (1.000)	0.975 (1.000)	1.000 (1.000)
Ctx.Pnoc	0.037 (0.094)	0.288 (0.593)	0.613 (1.000)	1.000 (1.000)
Ctx.Ntsr	5.476e-06 (2.738e-05)	0.288 (0.593)	0.522 (1.000)	1.000 (1.000)
Ctx.Glt25d2	4.252e-13 (3.721e-12)	0.003 (0.020)	0.159 (1.000)	1.000 (1.000)
Ctx.Fthfd	1.000 (1.000)	1.000 (1.000)	0.997 (1.000)	0.971 (1.000)
Ctx.Cort	2.588e-05 (1.004e-04)	0.087 (0.337)	0.240 (1.000)	0.288 (1.000)
Ctx.Cnp	1.000 (1.000)	1.000 (1.000)	1.000 (1.000)	1.000 (1.000)
Epi	0.179 (0.368)	0.136 (0.396)	0.284 (1.000)	0.046 (0.323)
Epi.Chat	0.350 (0.679)	0.235 (0.547)	0.524 (1.000)	0.188 (1.000)
Spc	1.000 (1.000)	0.999 (1.000)	1.000 (1.000)	1.000 (1.000)
Spc.Chat	0.432 (0.756)	0.925 (1.000)	0.984 (1.000)	0.871 (1.000)
RetC.Cones	0.982 (1.000)	0.984 (1.000)	0.980 (1.000)	0.851 (1.000)

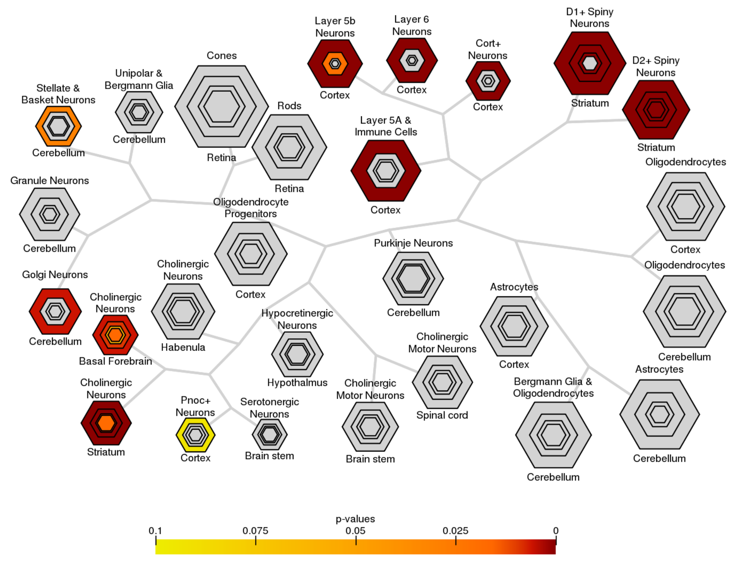

The overlapped genes for every cell type (pSI threshold 0.05 **) are:**
RetR.Rods : UBL7, GRM8, DLC1, ZFYVE28, CACNB2, ATP8A2, ARG2, ULK1, STAC2, ATP1A3, CRABP1, DPYSL4, CDR2, PITPNM3, ICMT, IMPG1
Hyp : NNAT, VASH2, ZDBF2, MAGEL2, HRH3, PNMA1, TRPM2, FRS3, PRKCD, GPX3, GBX2, ECEL1, RASD1, CPNE7
Hyp.Hcrt : NNAT, RESP18, FBXL2, CRHR2, PCSK1, ZCCHC12, PTPRN, VGF, SYT5, PNMA1, PRKCD, GPX3, NECAB2, RIMS3, CALY, CPNE7, ACHE, NELL1, RAB27B, LONRF2
BF : ACTN2, PDE10A, OPRK1, ZCCHC12, GPR83, GPRIN3, PRKAR2B, RAP2B, BCL11B, DMKN, HTR2C, SERTAD4, MEIS2, CSRNP3, RASD1, PCDH8, GPR149, GAD2, WIPF3, AKAP5, SLMAP, LHX8, RGS2, ZDBF2, SYNPO, DLX6, FRAS1, GNG7, DDN
BF.Chat : UCHL1, PCDH8, GPX3, DLL3, CRABP1, NTRK1, LHFPL5, SLC5A7, ECEL1, PCSK1, DGKB, ACHE, SNTG1, GPR83, RIMS3, SLC7A14, ISL1, LHX8, RESP18, GBX2, RPS27, YJEFN3
BS : P2RX5, CDH7, MCF2
BS.Chat : SLC7A14, ANXA6, PAQR9, SYT3, KCNC3, ECEL1, ADAM23, SLC45A1, HABP4, SLIT3, MXRA7, P2RX5, ACHE, RIMKLA, FBXO31, SLC5A7
BS.Slc6a4 : PCSK1, RESP18, GPX3, LHFPL5
Cb : CDC14B, KCNC1, ST3GAL2, EPHA8, PIP5K1C, CACNA1A, DIRAS2, HCN1, SHANK3, PCLO, PITPNM3, ADCY1, ITPR1, WSCD2
Cb.Septin4 : GPLD1, RPUSD3, GNAO1, ABCB9, GRIA1, KLF15, RASL11A, ARID5A, CDH22
Cb.Pcp2 : STEAP2, ORAI2, ARHGAP26, CLEC4G, FAM83D, CACNA2D2, KCNIP4, NUP93, ITGB1BP1, PPP4R4, ECM1, RAB40C, NCOA7, ICMT, KCNIP1, UBASH3B, SLC12A8, DUSP2, CDR2, ITPR1, NPTX1, FAM174B, DAGLA, TFG, DGKZ, MEF2D
Cb.Neurod1 : ST3GAL5, ARHGEF7, KLC2, JPH3, DAB2IP, KCNC1, EPN1, CAMK4, SYT2, EIF4E3, SPHKAP, CADM3, CAMKK2, ST3GAL2, KCNH3, ADCY1, ANKRD34A, WSCD2, FGF14, TFG, DPYSL4, DIRAS2
Cb.Lypd6 : GRM8, CAMK1G, GDPD5, SLC24A3, GABRA1, MGAT5B, TRPM2, EPHA8, MAPK13, BZW2, PAM, HUNK, STAC2, PRKCD, HCN1, PDE4A, CABP1, CNTN4, STEAP2, SORCS3
Cb.Grp : NNAT, TPBG, WNT10A, GSG1L, RPUSD3, RASD1
Cb.Grm2 : SYT13, SLC26A8, STEAP2, RCAN2, LHFPL5, RGS7, KLC2, UGCG, DIRAS1, VASH2, FGF12, KRT222, EFNA5, MGAT5B, MAGEL2, GAD1, KCNH3, CPLX3, PTPRN2, MXRA7, PLCL2, RASGRP2, GDPD5, NCS1, CLVS1, PAM, RAP1GAP2
Cb.Fthfd : GPLD1, RPUSD3, ABCB9, ABHD14A, RASL11A, CDH22
Cb.Cnp : FHDC1, RPP40, DUSP19, SEZ6L2, SRD5A1, GNAO1, PLD6, ARID5A
Cpu : COCH, NETO2, ACTN2, PDE10A, GNAL, GLCE, PRKAR2B, RIC8B, LINGO3, GPRIN3, CRABP1, PPP3CA, DOCK4, PRKCH, RIN1, BCL11B, DMKN, KCNA4, FBXL16, BAIAP2, PTPN5, KCNQ5, RAP2B, SERTAD4, DDIT4L, NGEF, ABLIM2, GPR83, GABRA4, SCN4B, MEIS2, MN1, RGS2, CPNE5, ARG2, MCTP1, WIPF3, MYT1L, SEZ6, PDP1, AKAP5, CHSY3, LHX8, RASGRP2, SH3RF2, SH2D5, DBN1, FGF14, RUNX1T1, KCNS2, SYNPO, FOXP1, DLX6, FRAS1, GNG7, DDN, EGR1, DCC
Cpu.D2 : SPEG, TANC2, CDK17, RIC8B, LINGO3, JPH3, DOCK4, RASGEF1A, NETO2, MATK, SMPD3, RASGEF1B, NGEF, TRIM46, CAMKK2, MEIS2, AFF3, WIPF3, MYT1L, AKAP5, CCM2, DOCK3, EXTL1, SH3RF2, SH2D5, CACNA2D3, CACNB2, HRH3, SYNPO, DLX6, DLX5, ORAI2, GNG7, FBXO41, ACTN2, DGAT2, GNAL, PRKAR2B, TEF, CAP2, ICAM5, GPRIN3, PPP3CA, DPF1, DUSP8, DMKN, SIPA1L1, PTPN5, SERTAD4, RPRML, CALN1, CPNE5, RAB15, MCTP1, ITPR1, RGS14, HIVEP2, CYLD, PAM, FBXL16, NECAB2, BAIAP2, MARK1, DLG2, ATXN1, PRKCH, RIN1, DTNB, LDB2, PMEPA1, DACT2, SHANK3, SCN4B, GPR149, ARG2, COCH, RASGRP2, CRTC1, RUNX1T1, FOXP1, DDN, PDE10A, SLITRK5, RASL10B, BCL11B, NPAS2, KCNA4, ZFYVE28, ABLIM2, MN1, PLCL2, LHX8, DAPK1, SNX27
Cpu.D1 : SPEG, PIK3R2, RIC8B, LINGO3, MPPED1, JPH3, DOCK4, RASGEF1A, PPP4R4, BAIAP2, MARK1, SMPD3, F12, RASGEF1B, NGEF, DGKB, TRIM46, CAMKK2, MEIS2, WIPF3, MYT1L, AKAP5, DOCK3, EXTL1, SH3RF2, SH2D5, HRH3, CACNB2, SYNPO, DLX6, DLX5, ORAI2, GNG7, ACTN2, DGAT2, GNAL, ADD2, PRKAR2B, TEF, CPLX2, ICAM5, GPRIN3, PPP3CA, SEH1L, DMKN, SIPA1L1, PTPN5, SERTAD4, DDIT4L, RPRML, CALN1, CPNE5, RAB15, MCTP1, SEZ6, ITPR1, RGS14, CAMSAP1, HIVEP2, CYLD, NECAB2, MAPK1, MATK, ATXN1, ENC1, KCNV1, PRKCH, RIN1, DTNB, LDB2, SHANK3, GABRA4, SCN4B, ARG2, COCH, ISL1, RASGRP2, CRTC1, FOXP1, DDN, PDE10A, SLITRK5, RASL10B, BCL11B, NPAS2, KCNA4, KCNQ5, DUSP8, RASGRP1, ZFYVE28, CDO1, ABLIM2, PIK3CB, MN1, DAPK1, CAMK2A
Cpu.Chat : PBX3, RCAN2, PCSK1, GPX3, SNTG1, CRABP1, DIRAS1, NTRK1, FGF12, VAMP2, DLL3, LHFPL5, CPNE7, ACHE, MGAT5B, SLC5A7, GPR83, RIMS3, MCTP1, VSTM2L, ACTL6B, MXRA7, UCHL1, SLC7A14, ISL1, LHX8, MAP1LC3A, CACNB3, GSG1L, ECEL1, TRPC7, DLX6, GBX2, CDK5R2
Ctx : ENC1, TANC2, MPPED1, PGM2L1, OPCML, UNC5D, KCNV1, RAP2B, RASGEF1A, LDB2, SIPA1L1, PSD3, STX1A, KCNQ5, DDIT4L, CSRNP3, NGEF, EFNA5, MAL2, PCLO, CAMKK2, WIPF3, LINGO1, DLGAP1, KCNH1, SYT16, EXTL1, DCLK1, LRRC4, GSG1L, GPRIN1, SYNPO, CAMK2A, EGR1, DDN
Ctx.Pdgfrajd340 : CDO1, PLEKHA2
Ctx.Etv1_ts88 : SLC7A8, DLGAP2, SLITRK3, ADD2, UNC5A, ICAM5, CACNA1A, MPPED1, DPYSL4, CX3CL1, KCNV1, RIN1, CRMP1, SLIT3, SLC8A2, STX1A, BAIAP2, KCNQ5, SYT5, DDIT4L, RASGRP1, NGEF, ENC1, CPNE7, RGS14, TRIM46, AP3B2, ALOX12B, ANXA6, GRIN1, L1CAM, CAMK1G, DLG3, LINGO1, DRP2, KCNH1, COCH, PITPNM3, DOCK3, KCNH3, NPTX1, CCKBR, SLC7A4, PAM, EPHA4, ATP1A3, CAMK2A, SORCS3, GPRIN1
Ctx.Pnoc : KCNIP1, DLGAP2, TMEM130, THY1, BASP1, PARM1, SYT1, CPLX3, GAD2, UGCG, CRABP1, CORT, YJEFN3, SLMAP
Ctx.Ntsr : NRXN3, DLGAP2, GABRG2, UNC5A, SLC4A10, PRKCE, ICAM5, CHSY3, CPNE4, OPCML, KCNV1, RIN1, SLIT3, SLC8A2, STX1A, CACNA1A, RTN4RL1, RASGRP1, NELL1, CDHR1, PCDH8, GRIN1, MCTP1, CPLX3, LINGO1, SYT16, SV2B, NPTX1, GRIA3, SLC8A1, EPHA4, FRAS1, SORCS3
Ctx.Glt25d2 : OPA1, SLC6A7, SLITRK3, DGAT2, ADD2, UNC5A, SLC6A17, CAP2, ICAM5, MPPED1, DPYSL4, GPRIN1, KCNV1, IGSF3, PLD3, LONRF2, CAMK1G, SLIT3, NPAS2, DACT2, SLC8A2, UNC13A, MATK, PARM1, SYT1, SYT5, RASGRP1, RIN1, ENC1, DLGAP2, PARG, NELL1, PNMA1, LDB2, DLG3, FAM131A, LINGO1, COCH, AKAP5, SV2B, EXTL1, GRIA3, CCKBR, SLC7A4, PAM, CX3CL1, ATP6V1H, MAPK1, CAMK2A, SORCS3, ATP8A1
Ctx.Fthfd : ARID5A, DDIT4L, KLF15
Ctx.Cort : DLGAP2, RCAN2, SLC4A10, KLC2, ICAM5, DLL3, CORT, KCNV1, WNT10A, EPN1, CAMK4, KCNQ5, PCLO, GRIA3, HECW1, KCNH3, DLGAP1, PITPNM3, DACT2, CCKBR, EPHA4, GPD1L, FRAS1, REPS2, MEF2D, GPRIN1
Ctx.Cnp : FHDC1, SEZ6L2, PLEKHA2, GNAO1, SRD5A1, FAM83D, TMOD1, ATP8A1
Epi : FHDC1, DMKN, VASH2, GABRA4, GPR151, KCNS2, ZCCHC12, RASD1, HTR2C, MCF2, PRKCD, GBX2, CPNE4, UBL7, RASL11A, CIT, CPNE7, PDP1, CDH8, PRKCH
Epi.Chat : KCTD8, PAQR9, CPNE4, ANKRD34A, PRKCH, UBL7, KCNS2, GDAP1L1, RTN4RL1, RASGEF1B, DPP6, SLC5A7, KCNIP1, ADAM11, GPR151, CNNM2, RASSF7, VSTM2L, NNAT, GDPD5, CACNB3, NECAB2, CDH8, YJEFN3
Spc : GPLD1, SCN4B, STEAP2, HECW1, GABRA5, SLC8A1, MCF2, HTR2C, P2RX5, ATP8A1
Spc.Chat : CDHR1, RIMKLA, SLIT3, KCNH2, ATP8A2, P2RX5, MXRA7, SYT3, SYT2, ECEL1, CHGA, ADAM23, SLC45A1, GRIK4, LHFPL5, STMN2, CDR2, SLC7A14, CNTN4, SEMA3A, SLC5A7
RetC.Cones : OPA1, PBX3, STEAP2, SESN3, UGCG, TEF, LINGO3, RGMB, SLK, WDR47, HYOU1, NDFIP1, UNC13A, SLC8A3, ZFAND5, SYT1, BEND4, ADARB1, SPHKAP, PREPL, CDHR1, CPLX3, HCN1, MXRA7, ARHGAP32, GPR162, ISL1, SLMAP, CACNA1D, SV2B, DBN1, CACNA2D3, SRPRB, EPHA8, CCKBR

The overlapped genes for every cell type (pSI threshold 0.01 **) are:**
RetR.Rods : DPYSL4, IMPG1, UBL7
Hyp : RASD1, MAGEL2
Hyp.Hcrt : NNAT, RESP18, CRHR2, PCSK1, ZCCHC12, PTPRN, VGF, SYT5, PNMA1, GPX3, RIMS3, CPNE7
BF : LHX8, OPRK1, HTR2C, GAD2
BF.Chat : LHX8, ACHE, NTRK1, SLC5A7, RIMS3, DLL3, GPX3, GBX2, ECEL1, CRABP1, SLC7A14, ISL1, RPS27
BS : none
BS.Chat : P2RX5, ACHE, SYT3, ECEL1, SLC7A14
BS.Slc6a4 : PCSK1, GPX3
Cb : ADCY1
Cb.Septin4 : RASL11A, ARID5A
Cb.Pcp2 : KCNIP1, DUSP2, STEAP2, FAM174B, DAGLA, SLC12A8, CLEC4G, ECM1, FAM83D, ITPR1, DGKZ, ITGB1BP1, ICMT
Cb.Neurod1 : KLC2, SYT2, WSCD2
Cb.Lypd6 : MAPK13, CNTN4, TRPM2, EPHA8, PRKCD, HCN1, CABP1, MGAT5B, SORCS3
Cb.Grp : NNAT, GSG1L, TPBG, WNT10A
Cb.Grm2 : RASGRP2, SLC26A8, CLVS1, LHFPL5, KCNH3, KLC2, MXRA7, EFNA5
Cb.Fthfd : ABCB9, RPUSD3
Cb.Cnp : FHDC1, SRD5A1, PLD6
Cpu : BCL11B, DMKN, SH3RF2, SCN4B, ACTN2, PDE10A, GNAL, MEIS2, FOXP1, WIPF3, NGEF, GPRIN3, GNG7, AKAP5
Cpu.D2 : ACTN2, DGAT2, ICAM5, GPRIN3, PRKCH, RIN1, BCL11B, SMPD3, CRTC1, SIPA1L1, NPAS2, KCNA4, PDE10A, PTPN5, DUSP8, SERTAD4, GPR149, RASGEF1A, SHANK3, ABLIM2, CAMKK2, SCN4B, MEIS2, MN1, CPNE5, ARG2, WIPF3, AKAP5, SH3RF2, HRH3, CYLD, FOXP1, DLX6, DLX5, RASL10B, GNG7, DDN
Cpu.D1 : ACTN2, ATXN1, TEF, RASL10B, GPRIN3, DOCK4, PRKCH, RASGEF1A, BCL11B, DMKN, NPAS2, PPP4R4, KCNA4, MATK, PTPN5, SMPD3, F12, RASGEF1B, NGEF, RPRML, SERTAD4, SCN4B, MEIS2, MN1, CPNE5, RGS14, MCTP1, WIPF3, PDE10A, COCH, AKAP5, ITPR1, DOCK3, RIN1, SH3RF2, SH2D5, CRTC1, CYLD, SLITRK5, FOXP1, DLX6, DLX5, GNG7, HRH3, DDN
Cpu.Chat : LHX8, GPR83, ACHE, SLC5A7, SLC7A14, CACNB3, RIMS3, MXRA7, DLL3, VSTM2L, RCAN2, SNTG1, ECEL1, CRABP1, NTRK1, ISL1, GBX2
Ctx : SYT16, EGR1, SYNPO, CAMK2A, MPPED1
Ctx.Pdgfrajd340 : CDO1, PLEKHA2
Ctx.Etv1_ts88 : ADD2, LINGO1, KCNV1
Ctx.Pnoc : GAD2, CPLX3, CORT, YJEFN3, SYT1
Ctx.Ntsr : CDHR1, GRIA3, ICAM5, CHSY3, EPHA4
Ctx.Glt25d2 : SV2B, SLITRK3, UNC13A, CCKBR, SLC7A4, GPRIN1, ICAM5, MPPED1, KCNV1, SORCS3
Ctx.Fthfd : ARID5A
Ctx.Cort : WNT10A, DLGAP2, CCKBR, DLL3, KCNH3, CORT
Ctx.Cnp : SRD5A1, GNAO1, FAM83D
Epi : VASH2, CIT, GPR151, HTR2C, PRKCD, GBX2, RASD1, CPNE7, PRKCH
Epi.Chat : NNAT, GPR151, KCTD8, KCNIP1, CACNB3, YJEFN3, VSTM2L, NECAB2, CPNE4, CNNM2, CDH8, PRKCH
Spc : P2RX5, MCF2
Spc.Chat : P2RX5, SLC45A1, RIMKLA, SEMA3A, SLC5A7
RetC.Cones : CDHR1, HYOU1, PREPL, SLC8A3, ZFAND5, EPHA8, CCKBR, TEF, SYT1, CPLX3, SESN3, SLK, RGMB, SPHKAP, ISL1, SLMAP

The overlapped genes for every cell type (pSI threshold 0.001 **) are:**
RetR.Rods : IMPG1
Hyp : MAGEL2
Hyp.Hcrt : NNAT, PCSK1, GPX3, RIMS3
BF : none
BF.Chat : LHX8, ECEL1, GBX2, CRABP1, NTRK1, ISL1, SLC5A7
BS : none
BS.Chat : P2RX5
BS.Slc6a4 : none
Cb : none
Cb.Septin4 : RASL11A
Cb.Pcp2 : SLC12A8, CLEC4G, ECM1
Cb.Neurod1 : none
Cb.Lypd6 : CABP1, PRKCD, MAPK13
Cb.Grp : WNT10A, TPBG, NNAT
Cb.Grm2 : CLVS1
Cb.Fthfd : RPUSD3
Cb.Cnp : none
Cpu : GPRIN3
Cpu.D2 : BCL11B, SH3RF2, SCN4B, ACTN2, PDE10A, MN1, HRH3, ARG2, SMPD3, FOXP1, WIPF3, RASL10B, GPRIN3, DLX5, GNG7, RIN1, DDN
Cpu.D1 : BCL11B, DMKN, SH3RF2, NPAS2, ACTN2, PDE10A, MN1, SERTAD4, FOXP1, AKAP5, PRKCH, RIN1, DOCK3
Cpu.Chat : LHX8, NTRK1, SLC7A14, SNTG1, DLL3, VSTM2L, ECEL1, ACHE, SLC5A7
Ctx : none
Ctx.Pdgfrajd340 : CDO1
Ctx.Etv1_ts88 : LINGO1
Ctx.Pnoc : CORT, YJEFN3
Ctx.Ntsr : CHSY3
Ctx.Glt25d2 : SLC7A4, MPPED1
Ctx.Fthfd : ARID5A
Ctx.Cort : KCNH3, CORT
Ctx.Cnp : SRD5A1
Epi : RASD1, PRKCD, GPR151, GBX2
Epi.Chat : KCNIP1, GPR151, CNNM2, CACNB3, KCTD8, NECAB2
Spc : none
Spc.Chat : P2RX5
RetC.Cones : CDHR1, ZFAND5, SLC8A3, CCKBR, SYT1, CPLX3

The overlapped genes for every cell type (pSI threshold 0.0001 **) are:**
RetR.Rods : none
Hyp : none
Hyp.Hcrt : PCSK1, GPX3, NNAT
BF : none
BF.Chat : LHX8, ECEL1, CRABP1, NTRK1, ISL1, SLC5A7
BS : none
BS.Chat : none
BS.Slc6a4 : none
Cb : none
Cb.Septin4 : none
Cb.Pcp2 : SLC12A8, CLEC4G, ECM1
Cb.Neurod1 : none
Cb.Lypd6 : MAPK13
Cb.Grp : NNAT
Cb.Grm2 : none
Cb.Fthfd : none
Cb.Cnp : none
Cpu : none
Cpu.D2 : BCL11B, SH3RF2, SCN4B, ACTN2, PDE10A, MN1, HRH3, ARG2, FOXP1, GPRIN3, GNG7, RIN1
Cpu.D1 : DMKN, SH3RF2, PRKCH, MN1
Cpu.Chat : LHX8, SLC7A14, ECEL1, VSTM2L, ACHE, NTRK1, SNTG1
Ctx : none
Ctx.Pdgfrajd340 : none
Ctx.Etv1_ts88 : none
Ctx.Pnoc : none
Ctx.Ntsr : none
Ctx.Glt25d2 : none
Ctx.Fthfd : ARID5A
Ctx.Cort : CORT
Ctx.Cnp : none
Epi : RASD1, PRKCD, GBX2, GPR151
Epi.Chat : KCNIP1, KCTD8, CNNM2, CACNB3, NECAB2, GPR151
Spc : none
Spc.Chat : P2RX5
RetC.Cones : CDHR1, CPLX3, SLC8A3, CCKBR, SYT1
