# Supplementary material for: Molecular Landscape of Tourette’s Disorder
Source: Int J Mol Sci. 2023 Jan 11;24(2):1428. doi: 10.3390/ijms24021428 (PMC9865021; doi:10.3390/ijms24021428)
Supplement: Supplementary file 1 [file supplementary File S8.pdf › supplementary File S8.pdf]

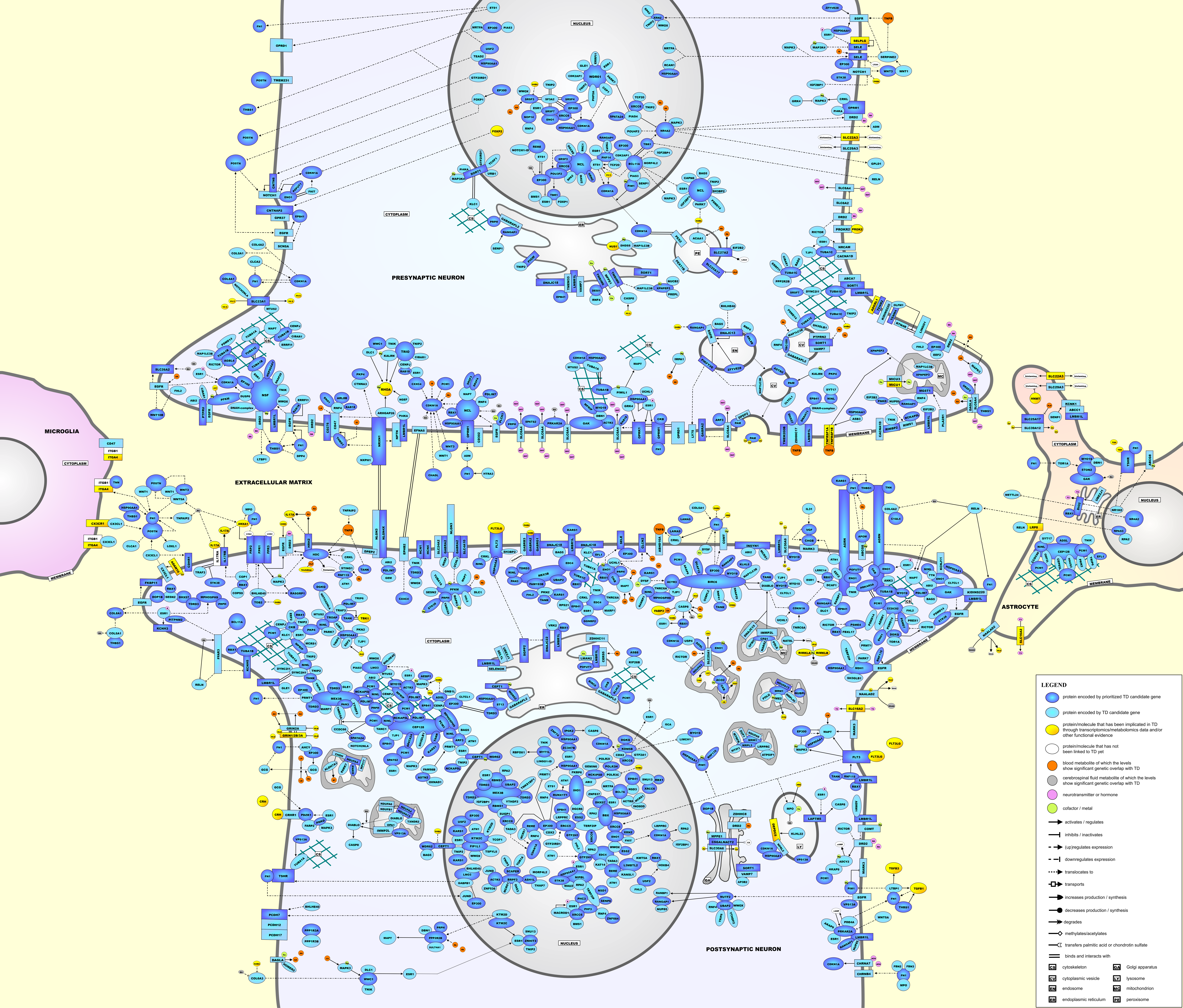

PRESYNAPTIC NEURON
POSTSYNAPTIC NEURON
EXTRACELLULAR MATRIX
MICROGLIA
ASTROCYTE
NUCLEUS
CYTOPLASM
MEMBRANE
LEGEND
protein encoded by prioritized TD candidate gene
protein encoded by TD candidate gene
protein/molecule that has been implicated in TD through transcriptomics/metabolomics data and/or other functional evidence
protein/molecule that has not been linked to TD yet
blood metabolite of which the levels show significant genetic overlap with TD
cerebrospinal fluid metabolite of which the levels show significant genetic overlap with TD
neurotransmitter or hormone
cofactor / metal
activates / regulates
inhibits / inactivates
(up)regulates expression
downregulates expression
translocates to
transports
increases production / synthesis
decreases production / synthesis
degrades
methylates/acetylates
transfers palmitic acid or chondroitin sulfate
binds and interacts with
CS cytoskeleton
CV cytoplasmic vesicle
EN endosome
ER endoplasmic reticulum
GA Golgi apparatus
LY lysosome
MC mitochondrion
PE peroxisome
