# Supplementary material for: Molecular Landscape of Tourette’s Disorder
Source: Int J Mol Sci. 2023 Jan 11;24(2):1428. doi: 10.3390/ijms24021428 (PMC9865021; doi:10.3390/ijms24021428)
Supplement: Supplementary file 1 [file ijms-24-01428-s001.zip › supplementary File S4.pdf]

Of the inputed 233 genes,
167 genes exist in the brain cell type expression dataset, 66 genes do not, which are:
PGAP1,MIR663B,KLRB1,AGBL1,RNU7-7P,IL26,EPB41L4B,MIR1252,RFPL1,SIAH3,HHIPL2,CHAT,HIPK4,NWD2,ALKAL2,MC4R,OLFM4,LINC01705,STRIP2,DLX6-AS1,KIR3DX1,SLC10A4,MIR199B,MIR1298,TAC3,SNORA35,LINC00523,PRSS35,DRD1,MIR153-2,CST1,ERICH3,OBSCN-AS1,CST4,DCHS2,DIRAS3,RIMBP2,TINCR,PNMA8A,SERTM1,SIRPB1,CHRM2,NAT16,ASIC2,CST2,MIR764,MXRA5,PHF24,ATP2B3,SLC27A6,KIAA0087,RNVU1-18,MIR1912,MIR1911,SLC17A8,DRD5,HCN4,AZU1,CACNG8,RXFP1,RNU5B-3P,GNRH1,LINC00269,C11ORF87,MIR448,RADX

Interpretation of CSEA results table:
The first row lists the pSI thresholds, and the first column lists the brain cell types
The values in the tables are the Fisher's Exact p-values, followed by the Benjamini-Hochberg (BH) corrected values in parentheses.
Click the linked p-values to see the overlapped genes for the corresponding brain cell types and thresholds.

Brain Cell Types and P-Values	0.05	0.01	0.001	0.0001
RetR.Rods	0.696 (0.761)	0.313 (0.438)	0.336 (0.657)	0.590 (0.938)
Hyp	1.080e-05 (4.199e-05)	0.039 (0.103)	0.004 (0.031)	0.150 (0.584)
Hyp.Hcrt	0.112 (0.178)	0.069 (0.151)	0.546 (0.778)	0.417 (0.729)
BF	1.789e-07 (1.565e-06)	1.059e-06 (9.269e-06)	0.107 (0.405)	1.000 (1.000)
BF.Chat	5.368e-09 (6.262e-08)	1.650e-07 (1.925e-06)	1.064e-05 (1.861e-04)	6.233e-06 (1.091e-04)
BS	0.070 (0.122)	0.085 (0.174)	1.000 (1.000)	1.000 (1.000)
BS.Chat	0.095 (0.158)	0.109 (0.212)	0.585 (0.778)	0.394 (0.727)
BS.Slc6a4	7.837e-04 (0.002)	0.505 (0.589)	1.000 (1.000)	1.000 (1.000)
Cb	0.190 (0.277)	0.483 (0.583)	0.387 (0.657)	0.150 (0.584)
Cb.Septin4	0.572 (0.646)	0.330 (0.444)	0.390 (0.657)	0.498 (0.831)
Cb.Pcp2	0.478 (0.597)	0.409 (0.511)	0.394 (0.657)	0.295 (0.722)
Cb.Neurod1	0.305 (0.427)	0.240 (0.381)	0.092 (0.405)	1.000 (1.000)
Cb.Lypd6	0.046 (0.094)	0.010 (0.037)	0.024 (0.141)	0.011 (0.094)
Cb.Grp	0.059 (0.109)	0.021 (0.068)	0.387 (0.657)	0.241 (0.722)
Cb.Grm2	0.053 (0.103)	1.000 (1.000)	1.000 (1.000)	1.000 (1.000)
Cb.Fthfd	0.532 (0.620)	0.602 (0.680)	0.267 (0.623)	0.322 (0.722)
Cb.Cnp	1.000 (1.000)	1.000 (1.000)	1.000 (1.000)	1.000 (1.000)
Cpu	3.976e-04 (0.001)	0.171 (0.286)	1.000 (1.000)	1.000 (1.000)
Cpu.D2	0.332 (0.447)	0.155 (0.271)	0.558 (0.778)	0.330 (0.722)
Cpu.D1	0.027 (0.060)	0.065 (0.151)	0.149 (0.405)	0.260 (0.722)
Cpu.Chat	3.014e-06 (1.318e-05)	5.910e-08 (1.775e-06)	1.241e-06 (4.345e-05)	2.351e-06 (8.230e-05)
Ctx	5.333e-07 (3.702e-06)	5.730e-06 (3.342e-05)	1.000 (1.000)	1.000 (1.000)
Ctx.Pdgfrajd340	0.892 (0.946)	1.000 (1.000)	1.000 (1.000)	1.000 (1.000)
Ctx.Etv1_ts88	6.346e-07 (3.702e-06)	0.041 (0.103)	0.194 (0.484)	0.371 (0.722)
Ctx.Pnoc	2.667e-06 (1.318e-05)	0.001 (0.006)	0.001 (0.013)	6.176e-05 (7.205e-04)
Ctx.Ntsr	1.221e-14 (4.273e-13)	1.014e-07 (1.775e-06)	0.150 (0.405)	1.000 (1.000)
Ctx.Glt25d2	2.022e-11 (3.539e-10)	1.698e-06 (1.189e-05)	0.150 (0.405)	1.000 (1.000)
Ctx.Fthfd	0.516 (0.620)	0.305 (0.438)	0.377 (0.657)	1.000 (1.000)
Ctx.Cort	7.461e-04 (0.002)	6.429e-04 (0.003)	0.019 (0.130)	0.072 (0.395)
Ctx.Cnp	1.000 (1.000)	1.000 (1.000)	1.000 (1.000)	1.000 (1.000)
Epi	0.001 (0.003)	0.149 (0.271)	0.459 (0.730)	0.269 (0.722)
Epi.Chat	0.004 (0.010)	0.020 (0.068)	0.143 (0.405)	0.057 (0.395)
Spc	0.358 (0.465)	0.307 (0.438)	0.505 (0.768)	0.296 (0.722)
Spc.Chat	0.006 (0.013)	0.040 (0.103)	0.600 (0.778)	0.363 (0.722)
RetC.Cones	0.188 (0.277)	0.344 (0.446)	0.129 (0.405)	0.079 (0.395)

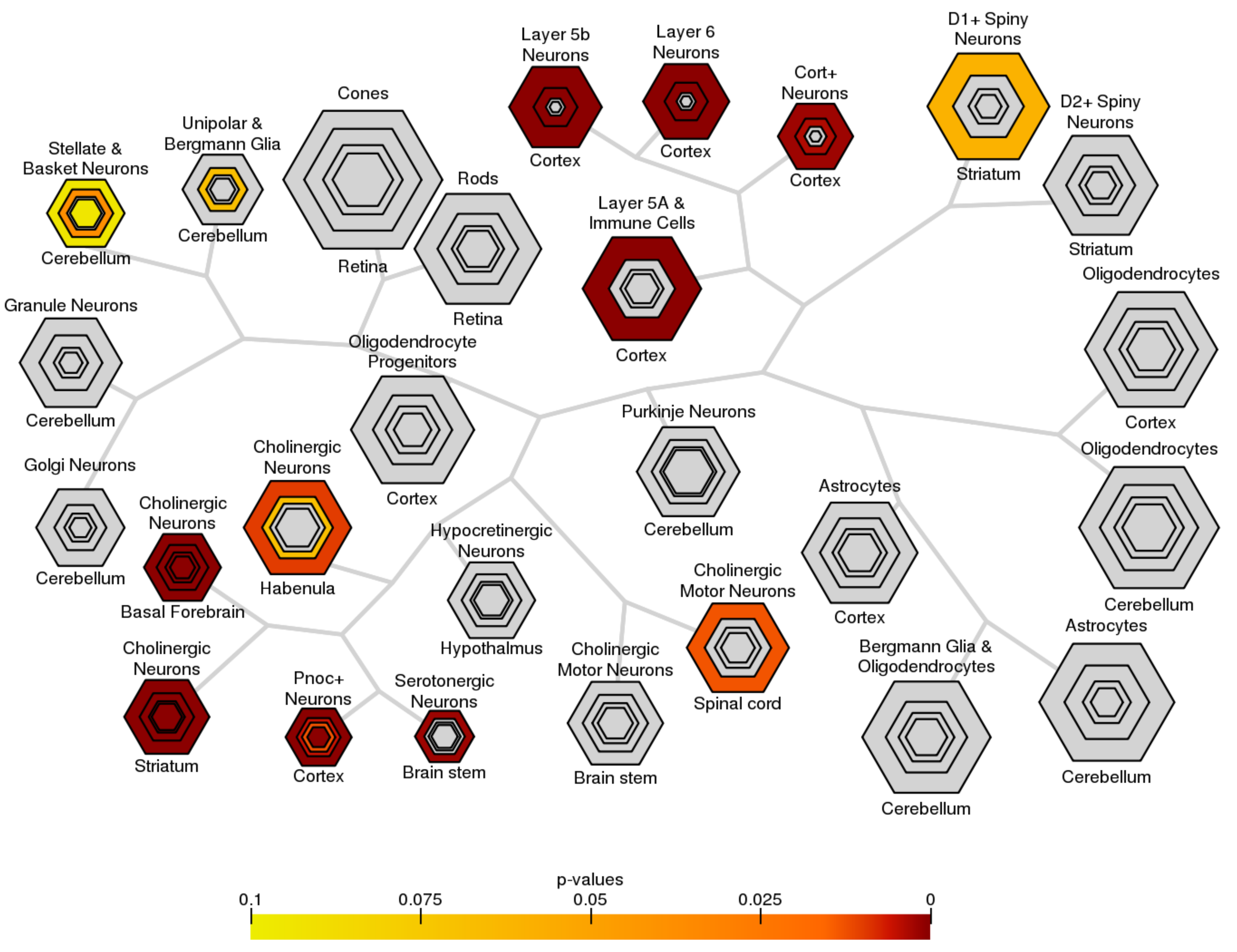

The overlapped genes for every cell type (pSI threshold 0.05 **) are:**
RetR.Rods : NT5E, C1QL2, COL20A1, PLCH1, CRABP1, SLC17A7
Hyp : NNAT, PNOC, C1QL2, NPY2R, MAGEL2, KANK4, ECEL1, SST, DGKK, PRLR, GPR101, GBX2, ADCYAP1
Hyp.Hcrt : NNAT, ZCCHC12, RESP18, VGF, DGKK, RAB27B
BF : LHX8, GJB2, PCDH8, PNOC, NPY2R, OPRK1, ZCCHC12, GPR83, HTR2C, SST, GPR101, GDA, CRYM, CRH, AKAP5
BF.Chat : LHX8, PCDH8, GNG2, NTRK1, SLC18A3, KANK4, RESP18, GPR83, ECEL1, DGKK, TRPC3, GBX2, ELAVL2, CRABP1, SLC7A14, SLC5A7
BS : GJB2, SLC27A2, KANK4, TTR, SOSTDC1, CRH
BS.Chat : SLIT3, ECEL1, LCN2, SLC18A3, ADCYAP1, SLC7A14, SLC5A7
BS.Slc6a4 : CBLN4, GNG2, SLC27A2, RESP18, DGKK, ADCYAP1, CRH
Cb : NEUROD2, GPCPD1, CASQ2, TTR, KIT, TRPC3, FAT2, HCN1, DIRAS2
Cb.Septin4 : COLEC12, DIO2, NPY, P2RY1, BTBD17, WIF1, GPD1
Cb.Pcp2 : NPTX1, FOXQ1, TRPC3, CACNA2D2, CASQ2
Cb.Neurod1 : NEUROD2, SYT2, FOS, FAT2, SPHKAP, DIRAS2
Cb.Lypd6 : GABRA1, CDH1, KIT, HCN1, PLCH1, SORCS3
Cb.Grp : NNAT, BTBD17, TRPC3, WIF1, ADCYAP1, NEUROD6
Cb.Grm2 : PNOC, SYT13, MAGEL2, FGF12, GAD1, KIT, ELAVL2
Cb.Fthfd : DIO2, NPY, COLEC12, TTR, BTBD17, HES1, WIF1, GPD1
Cb.Cnp : none
Cpu : LHX8, NT5E, GPR83, SH3RF2, SH2D5, GRIN2B, CRYM, SST, SEZ6, CRABP1, AKAP5
Cpu.D2 : LHX8, NT5E, SH3RF2, SH2D5, SLITRK5, ICAM5, AKAP5
Cpu.D1 : SH3RF2, SH2D5, ENC1, GRIN2B, CRYM, SLITRK5, ICAM5, MPPED1, SEZ6, RASGRP1, AKAP5, FREM2
Cpu.Chat : LHX8, GPR83, NTRK1, WIF1, FGF12, ECEL1, DGKK, TRPC6, TRPC3, GBX2, SLC18A3, CRABP1, SLC7A14, SLC5A7
Ctx : CCK, NPY, PDE1A, NPAS4, TRHDE, RASAL1, GDA, MPPED1, PGM2L1, SYT16, VIP, ENC1, TBR1, NEUROD6
Ctx.Pdgfrajd340 : SEMA5A, HES1, NR4A1, C1QL2
Ctx.Etv1_ts88 : NPAS4, SLC17A7, SLIT3, NPTX1, PDE1A, RASGRP1, L1CAM, CASQ2, CLSTN2, SLC22A8, ICAM5, GDA, MPPED1, RASAL1, ITIH2, ISLR, NEUROD6, ENC1, TBR1, SORCS3, MOXD1
Ctx.Pnoc : CCK, GNG2, NPY, SYT1, SST, KIT, PNOC, CRABP1, CRH, VIP, NEUROD6
Ctx.Ntsr : GABRG2, SLC4A10, ICAM5, CPNE4, SLIT3, RASGRP1, BTG2, RASAL1, ISLR, TBR1, NEUROD6, CDHR1, PCDH8, NR4A2, PDE1A, GPR101, SYT16, CBLN4, SV2B, NPTX1, NPY2R, CLSTN2, SORCS3, MOXD1
Ctx.Glt25d2 : SLC17A7, ENC1, ICAM5, MPPED1, NPAS4, SLIT3, UNC13A, SYT1, RASGRP1, GDA, RASAL1, ADCYAP1, ISLR, PDE1A, AKAP5, SV2B, CCK, KLF2, CRYM, SLC22A8, SORCS3, MOXD1
Ctx.Fthfd : DIO2, CHRDL1, BTBD17, FAM163A, HES1, ISLR
Ctx.Cort : NPY, SLC4A10, SST, TRHDE, CLSTN2, TRPC6, ICAM5, CRH, MOXD1
Ctx.Cnp : RASAL1
Epi : CBLN4, WIF1, SEMA5A, ZCCHC12, HTR2C, FAM163A, SOSTDC1, GBX2, CPNE4, CCDC141, CDH8
Epi.Chat : NNAT, SLC18A3, NR4A2, CBLN4, PDE1A, SEMA5A, SOSTDC1, FAM163A, CPNE4, CCDC141, CDH8, SLC5A7
Spc : SEMA5A, GPCPD1, GJB2, SLC27A2, KANK4, MPZ, HTR2C, GPR101
Spc.Chat : CDHR1, PNOC, C1QL2, SYT2, ECEL1, LCN2, SLIT3, SLC18A3, SLC7A14, SEMA3A, SLC5A7
RetC.Cones : CDHR1, SV2B, DIO2, GPCPD1, GABRA3, UNC13A, KLF2, SYT1, MYOCD, HCN1, CRYM, BTG2, SPHKAP, MOXD1

The overlapped genes for every cell type (pSI threshold 0.01 **) are:**
RetR.Rods : C1QL2, NT5E, SLC17A7, PLCH1
Hyp : PRLR, GPR101, MAGEL2
Hyp.Hcrt : NNAT, ZCCHC12, VGF, RESP18
BF : LHX8, NPY2R, OPRK1, HTR2C, GPR101, CRYM, CRH
BF.Chat : LHX8, NTRK1, ECEL1, DGKK, TRPC3, GBX2, SLC18A3, CRABP1, SLC7A14, SLC5A7
BS : KANK4, TTR
BS.Chat : SLC7A14, ECEL1, SLC18A3, LCN2
BS.Slc6a4 : DGKK
Cb : KIT, FAT2
Cb.Septin4 : P2RY1, GPD1, WIF1, BTBD17
Cb.Pcp2 : CASQ2, FOXQ1, TRPC3
Cb.Neurod1 : NEUROD2, SYT2, FAT2
Cb.Lypd6 : CDH1, KIT, HCN1, PLCH1, SORCS3
Cb.Grp : NNAT, TRPC3, WIF1, BTBD17
Cb.Grm2 : none
Cb.Fthfd : COLEC12, WIF1, BTBD17
Cb.Cnp : none
Cpu : SH3RF2, AKAP5
Cpu.D2 : NT5E, ICAM5, SH3RF2, AKAP5
Cpu.D1 : SLITRK5, SH3RF2, AKAP5, SH2D5, FREM2
Cpu.Chat : LHX8, GPR83, SLC7A14, ECEL1, TRPC6, TRPC3, GBX2, SLC18A3, CRABP1, NTRK1, SLC5A7
Ctx : MPPED1, GDA, SYT16, NPAS4, VIP, TBR1
Ctx.Pdgfrajd340 : none
Ctx.Etv1_ts88 : RASAL1, NPAS4, ISLR, TBR1, CLSTN2
Ctx.Pnoc : VIP, CRH, PNOC, SYT1, NPY
Ctx.Ntsr : CDHR1, NPY2R, GPR101, ICAM5, RASAL1, ISLR, MOXD1, TBR1, NEUROD6
Ctx.Glt25d2 : SV2B, PDE1A, UNC13A, CRYM, ICAM5, MPPED1, NPAS4, SORCS3
Ctx.Fthfd : FAM163A, CHRDL1, DIO2, BTBD17
Ctx.Cort : MOXD1, SST, TRPC6, CLSTN2, NPY
Ctx.Cnp : none
Epi : CBLN4, GBX2, HTR2C
Epi.Chat : CBLN4, NR4A2, SOSTDC1, CPNE4, NNAT, CDH8
Spc : SLC27A2, KANK4, MPZ
Spc.Chat : SLC5A7, C1QL2, SLC18A3, SEMA3A, LCN2
RetC.Cones : CDHR1, KLF2, SYT1, MYOCD, BTG2, SPHKAP, MOXD1

The overlapped genes for every cell type (pSI threshold 0.001 **) are:**
RetR.Rods : NT5E, SLC17A7
Hyp : PRLR, GPR101, MAGEL2
Hyp.Hcrt : NNAT
BF : NPY2R
BF.Chat : LHX8, ECEL1, GBX2, CRABP1, NTRK1, SLC5A7
BS : none
BS.Chat : SLC18A3
BS.Slc6a4 : none
Cb : FAT2
Cb.Septin4 : WIF1, BTBD17
Cb.Pcp2 : CASQ2, FOXQ1
Cb.Neurod1 : NEUROD2, FAT2
Cb.Lypd6 : CDH1, KIT, PLCH1
Cb.Grp : NNAT
Cb.Grm2 : none
Cb.Fthfd : COLEC12, BTBD17
Cb.Cnp : none
Cpu : none
Cpu.D2 : SH3RF2
Cpu.D1 : SH3RF2, AKAP5
Cpu.Chat : LHX8, NTRK1, ECEL1, TRPC3, SLC18A3, SLC7A14, SLC5A7
Ctx : none
Ctx.Pdgfrajd340 : none
Ctx.Etv1_ts88 : ISLR, TBR1
Ctx.Pnoc : VIP, CRH, PNOC, NPY
Ctx.Ntsr : NEUROD6
Ctx.Glt25d2 : MPPED1
Ctx.Fthfd : DIO2, BTBD17
Ctx.Cort : SST, MOXD1
Ctx.Cnp : none
Epi : GBX2
Epi.Chat : SOSTDC1, CBLN4, NR4A2
Spc : MPZ
Spc.Chat : LCN2
RetC.Cones : CDHR1, BTG2, MYOCD, SYT1, MOXD1

The overlapped genes for every cell type (pSI threshold 0.0001 **) are:**
RetR.Rods : NT5E
Hyp : GPR101
Hyp.Hcrt : NNAT
BF : none
BF.Chat : LHX8, CRABP1, NTRK1, ECEL1, SLC5A7
BS : none
BS.Chat : SLC18A3
BS.Slc6a4 : none
Cb : FAT2
Cb.Septin4 : WIF1
Cb.Pcp2 : CASQ2, FOXQ1
Cb.Neurod1 : none
Cb.Lypd6 : CDH1, KIT, PLCH1
Cb.Grp : NNAT
Cb.Grm2 : none
Cb.Fthfd : BTBD17
Cb.Cnp : none
Cpu : none
Cpu.D2 : SH3RF2
Cpu.D1 : SH3RF2
Cpu.Chat : LHX8, NTRK1, ECEL1, TRPC3, SLC18A3, SLC7A14
Ctx : none
Ctx.Pdgfrajd340 : none
Ctx.Etv1_ts88 : TBR1
Ctx.Pnoc : CRH, VIP, PNOC, NPY
Ctx.Ntsr : none
Ctx.Glt25d2 : none
Ctx.Fthfd : none
Ctx.Cort : SST
Ctx.Cnp : none
Epi : GBX2
Epi.Chat : CBLN4, SOSTDC1, NR4A2
Spc : MPZ
Spc.Chat : LCN2
RetC.Cones : CDHR1, MYOCD, SYT1, BTG2
