# Supplementary material for: Molecular Landscape of Tourette’s Disorder
Source: Int J Mol Sci. 2023 Jan 11;24(2):1428. doi: 10.3390/ijms24021428 (PMC9865021; doi:10.3390/ijms24021428)
Supplement: Supplementary file 1 [file ijms-24-01428-s001.zip › supplementary File S6.pdf]

Of the inputed 692 genes,
506 genes exist in the brain cell type expression dataset, 186 genes do not, which are:
HSPA6,NCF2,IGHG4,HBD,IGHG2,HBB,LILRB1-AS1,LDLRAD2,CHI3L2,FCGR1A,S100A11,IGKC,S100A12,MICB,C1ORF112,C1RL,NAPSB,TMEM92,LYZ,IGKJ1,IGKJ4,FAM110D,VSIR,HLA-DQB1,CEACAM4,HLA-DPB1,SMCO1,RNASE2,PRR29,CLEC17A,HLA-DRB1,OASL,TRIM22,HLA-DRB5,SIGLEC14,SRARP,SIGLEC10,SERPINB1,MS4A4A,LINC02590,MT1G,CXCL6,SYK,CXCL8,CLEC2B,SECTM1,MT1A,ARPC1B,CD300C,YBX3,CD300E,C10ORF105,CGAS,CLEC12A,TNFSF18,SNORD60,LILRB4,MT1X,LILRB2,LILRB1,TUBA1C,MT1M,MT1H,C4A,C4B,PIRT,C19ORF38,MT1B,FCN1,MT1JP,IL4R,TFEC,VNN2,AGAP11,GBP5,TNFRSF10A-AS1,PPP1R18,BTNL3,MS4A6A,TUBAL3,DEPP1,UBE2C,SIRPB2,MS4A6E,TRIM5,METTL2B,TESPA1,TNFRSF10C,TNFRSF10A,CD44-AS1,PTAFR,IGLC1,TNFRSF10D,PLP2,RNASET2,KIF19,NIBAN2,CIBAR2,SLC52A3,SPI1,TASL,CEACAM21,IRAG1,IFI16,KNL1,C7ORF61,TNFRSF6B,IGKJ5,ZBED2,IGHA1,ADGRE1,TMEM156,IL6R,C1ORF162,ADGRL4,C5ORF58,PLAAT4,SHC1,LILRA2,C16ORF54,MCEMP1,PCLAF,LRRC32,BCL2A1,OR7A5,SIGLEC7,PSMB8-AS1,HLA-DOA,HLA-DPA1,NABP1,LPAR5,MKI67,LCN6,MT2A,FAM111B,CPAMD8,FCGR3A,RNASE3,HILPDA,ADGRE2,ARL11,LRRC25,C1ORF87,CMAHP,FYB1,BIN2,SERPINA1,HLA-DRA,SERPINA3,TMIGD2,TLR5,TMEM255B,LILRA1,SIGLEC9,APOL4,APOL1,GNGT2,IGFLR1,HSPB1,TLDC2,TMPRSS3,FCGR2A,FAM216B,IL32,C1R,HBA1,MT1CP,HBA2,IGHM,JCHAIN,SBNO2,TAX1BP3,CASP5,MS4A14,CA1,ACKR1,NAMPTP1,HLA-DQA1,TYMP,SIGLEC8,ADH1B,ALOX15B,LILRA5,HLA-DMB,LILRA3,HLA-DMA

Interpretation of CSEA results table:
The first row lists the pSI thresholds, and the first column lists the brain cell types
The values in the tables are the Fisher's Exact p-values, followed by the Benjamini-Hochberg (BH) corrected values in parentheses.
Click the linked p-values to see the overlapped genes for the corresponding brain cell types and thresholds.

Brain Cell Types and P-Values	0.05	0.01	0.001	0.0001
RetR.Rods	0.529 (1.000)	0.239 (0.696)	0.010 (0.089)	0.051 (0.228)
Hyp	0.570 (1.000)	0.025 (0.134)	0.013 (0.094)	0.011 (0.198)
Hyp.Hcrt	0.198 (0.652)	0.504 (0.882)	0.213 (0.610)	0.486 (0.971)
BF	0.006 (0.037)	0.024 (0.134)	0.292 (0.610)	0.038 (0.228)
BF.Chat	0.369 (0.992)	0.442 (0.882)	1.000 (1.000)	1.000 (1.000)
BS	0.015 (0.071)	0.181 (0.633)	0.236 (0.610)	1.000 (1.000)
BS.Chat	0.082 (0.320)	0.313 (0.842)	0.271 (0.610)	0.063 (0.244)
BS.Slc6a4	0.946 (1.000)	0.884 (0.980)	0.719 (0.991)	1.000 (1.000)
Cb	0.975 (1.000)	0.728 (0.950)	0.435 (0.734)	1.000 (1.000)
Cb.Septin4	2.521e-04 (0.002)	5.312e-04 (0.006)	0.008 (0.088)	0.017 (0.198)
Cb.Pcp2	0.937 (1.000)	0.924 (0.980)	0.921 (1.000)	1.000 (1.000)
Cb.Neurod1	0.889 (1.000)	0.759 (0.950)	0.461 (0.734)	0.500 (0.971)
Cb.Lypd6	0.528 (1.000)	0.555 (0.883)	0.292 (0.610)	0.162 (0.516)
Cb.Grp	7.521e-04 (0.005)	0.041 (0.180)	0.181 (0.610)	0.201 (0.585)
Cb.Grm2	0.978 (1.000)	0.867 (0.980)	0.382 (0.704)	0.480 (0.971)
Cb.Fthfd	2.849e-06 (4.986e-05)	0.027 (0.134)	0.365 (0.704)	0.697 (1.000)
Cb.Cnp	0.895 (1.000)	0.498 (0.882)	0.651 (0.949)	0.555 (1.000)
Cpu	0.646 (1.000)	0.395 (0.882)	0.265 (0.610)	1.000 (1.000)
Cpu.D2	1.000 (1.000)	0.760 (0.950)	0.446 (0.734)	0.708 (1.000)
Cpu.D1	0.994 (1.000)	0.896 (0.980)	0.880 (1.000)	0.603 (1.000)
Cpu.Chat	0.735 (1.000)	0.547 (0.883)	0.823 (1.000)	1.000 (1.000)
Ctx	0.966 (1.000)	0.409 (0.882)	1.000 (1.000)	1.000 (1.000)
Ctx.Pdgfrajd340	0.762 (1.000)	0.201 (0.640)	0.043 (0.213)	0.052 (0.228)
Ctx.Etv1_ts88	5.949e-08 (2.082e-06)	8.425e-11 (2.949e-09)	1.020e-07 (3.570e-06)	5.678e-09 (1.987e-07)
Ctx.Pnoc	0.926 (1.000)	0.709 (0.950)	0.759 (0.991)	1.000 (1.000)
Ctx.Ntsr	0.969 (1.000)	1.000 (1.000)	1.000 (1.000)	1.000 (1.000)
Ctx.Glt25d2	0.939 (1.000)	1.000 (1.000)	1.000 (1.000)	1.000 (1.000)
Ctx.Fthfd	0.016 (0.071)	0.011 (0.095)	0.018 (0.103)	0.029 (0.228)
Ctx.Cort	0.940 (1.000)	0.629 (0.950)	1.000 (1.000)	1.000 (1.000)
Ctx.Cnp	0.949 (1.000)	0.889 (0.980)	0.764 (0.991)	0.793 (1.000)
Epi	0.300 (0.876)	0.378 (0.882)	0.556 (0.847)	0.243 (0.653)
Epi.Chat	0.984 (1.000)	0.467 (0.882)	0.205 (0.610)	0.271 (0.677)
Spc	3.502e-05 (4.085e-04)	1.658e-06 (2.901e-05)	2.236e-04 (0.004)	0.087 (0.305)
Spc.Chat	0.205 (0.652)	0.117 (0.457)	0.296 (0.610)	0.045 (0.228)
RetC.Cones	0.709 (1.000)	0.660 (0.950)	0.289 (0.610)	0.366 (0.855)

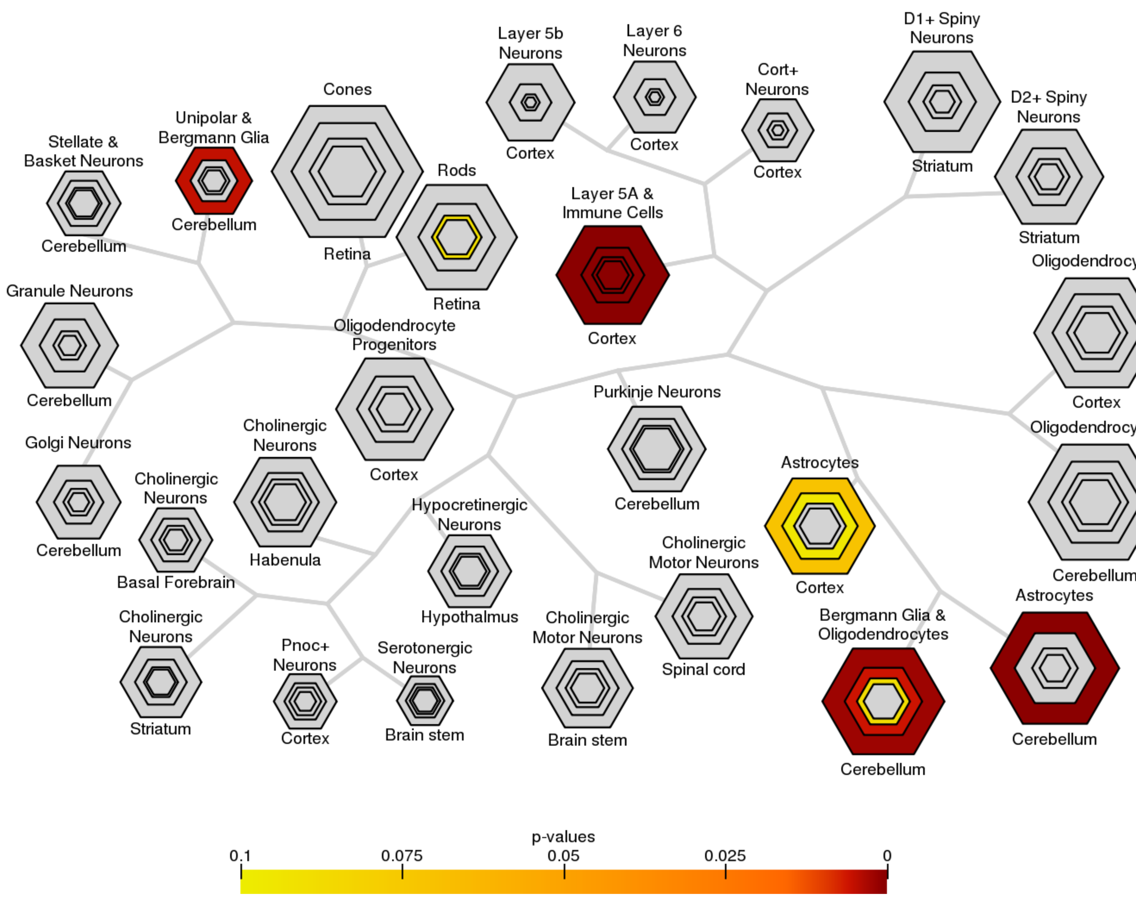

The overlapped genes for every cell type (pSI threshold 0.05 **) are:**
RetR.Rods : ALPL, SERPINH1, SLC26A2, PHYHD1, SLC16A3, CXCL16, HCLS1, AQP1, TRIP10, SLCO4A1, MYO7A, SEMA3F, TGFB3, TNC, ROM1, TNFAIP3, STC1, SUSD3, RTEL1, RHBDF2, CP
Hyp : OXT, AVP, C1QC, SYTL4, MYO7A, RAMP3, RASD1, SELPLG, RNASE6
Hyp.Hcrt : SLPI, TNFRSF12A, C2CD4B, EMP3, CASP1, IGFBP3, UCP2, IGHG1, PIK3R5, BACE2, ACSL5, HYAL2, LY96
BF : FCER1G, OXT, TAGLN, AVP, NUPR1, CD68, MGP, TYROBP, INHBA, IDO1, LY86, C1QA, IFITM1, IFITM3, RASD1, SRGN, CD163
BF.Chat : SLPI, CD44, LST1, IL1R2, CDA, RGS10, CDKN1A, TES, IGHG1
BS : TYROBP, ADAMTSL4, TAL1, MGP, GFAP, ELOVL7, TMPRSS5, FCER1G, IFITM1, CALCB, IGFBP2, C1QB, LY86, SRGN, RASSF4, C3
BS.Chat : CALCB, MAFF, TNFRSF12A, TSPO, TAGLN2, BIRC5, CDA, TNFRSF1A, CLIC1, LCP1, ANXA2, TUBB6, LGI2, PIK3R5, TES, C3, IRX3
BS.Slc6a4 : GPR4, LST1
Cb : PLSCR1, LYN, ITPKC, PIK3R3, SLA, PXN, RBMS2, RPS6KA1, CNKSR3, RTEL1, CCDC69, MYBPC1
Cb.Septin4 : CHI3L1, PHYHD1, KLF15, SLCO4A1, GPR146, UCP2, FGFRL1, PLSCR1, GSTT1, VAMP8, FXYD3, GLI1, DENND3, MYBPC1, RHOD, SLC14A1, IFITM2, EMP2, EMP3, STARD8, CNN2, LGR6, PXN, HCK, NPL, TNC, IRX3, HK2, TNFRSF1A, CLIC1, ANGPTL4, CASP1, PTH1R, CYBA, CDKN1A, TUBB6, ARID5A, CEBPD, TIMP1
Cb.Pcp2 : ALOX5AP, RHOD, IGFBP3, LCP1, STXBP2, IER3, RTEL1, CCDC69, LY96
Cb.Neurod1 : UNC13D, VAMP8, TNFRSF12A, ETS2, RAMP3, RPS6KA1, BCL6, CNKSR3, CCDC69, IRX3
Cb.Lypd6 : TMEM204, SLA, GADD45G, IFITM1, ST14, TIFA, PLVAP, ROM1
Cb.Grp : CHI3L1, RHOD, TAGLN, CTXN3, PLSCR1, HK2, IFITM2, CLIC1, VAMP8, ANGPTL4, SLCO4A1, GPR146, CXCL11, UCP2, SERPING1, RASD1, CLEC1A, CASP1, SLC14A1
Cb.Grm2 : COTL1, ST14, LGI2, GMNN, TNFRSF12A
Cb.Fthfd : MSX1, CHI3L1, GADD45G, PDLIM1, SLCO4A1, GPR146, CHSY1, UCP2, ANGPTL4, IFI30, ID3, CASP1, FGFRL1, PLSCR1, GSTT1, PTGS1, VAMP8, NUPR1, FXYD3, CTXN3, GLI1, IFITM2, VAMP5, DENND3, EIF4EBP1, MYBPC1, RHOD, SLC14A1, GFAP, EMP2, EMP3, PTH1R, CNN2, NPL, CCDC69, LY96, CD44, HK2, TNFRSF1A, PHYHD1, IFITM1, LHFPL2, SERPINH1, RRM2, CYBA, CDKN1A, TSPO, TIMP1
Cb.Cnp : SLPI, ITGB4, CSF1, ADAMTSL4, PDLIM1, ARRDC3, ELOVL7, ARID5A, TMPRSS5, EFNA1, LHFPL2, ST6GAL1, RASL12, CLDN14, RPS6KA1, RASSF4, IL18, CTSC, BACE2, MTHFD2
Cpu : AIF1, TYROBP, INHBA, IDO1, C1QB, C1QA, ARHGDIB, SELPLG, STC1
Cpu.D2 : CISH, AIF1, ACVRL1, IDO1, ARHGDIB, STC1
Cpu.D1 : CISH, AIF1, STXBP2, CD68, INHBA, ART3, IDO1, ACVRL1, ARHGDIB, STC1
Cpu.Chat : CD44, LST1, CDA, CASP1, RGS10, LGI2, IGHG1, TES
Ctx : FOSL2, KLF10, STARD8, ZBTB16
Ctx.Pdgfrajd340 : FGFRL1, ST6GAL1, TAGLN2, ASF1B, TNFRSF1A, SOCS3, CNN2, RRM2, IGFBP3, MELK, TGM2, RASL12, RELL1, AURKB, TOP2A, CDK2, BCL6B
Ctx.Etv1_ts88 : PTPN6, TYROBP, CYTH4, TCAP, ITGB2, IRF8, ID3, CFH, FCER1G, TAGLN, GMFG, FERMT3, C2CD4B, STAB1, IGFBP2, C1QB, CTSS, NCKAP1L, CD14, LYVE1, CTSC, AIF1, GFAP, F13A1, INPP5D, STARD8, CD163, LY86, PTGS1, NPL, CP, SLC11A1, CD68, CSF1R, LAPTM5, IFITM1, TNFAIP8L2, C1QC, CYBA, C1QA, ARHGDIB, TIFA, MS4A7, GBP6
Ctx.Pnoc : LST1, NNMT, ZBTB16
Ctx.Ntsr : KLF10, CSF1R, CFH, OXTR, PDLIM1
Ctx.Glt25d2 : TMEM204, CSF1R, STARD8, GBP2, ITGB2, TNFAIP3, LRG1
Ctx.Fthfd : CHI3L1, KLF15, GPR146, ID3, ABCC4, FGFRL1, SLC7A2, VAMP8, NUPR1, SERPINH1, IGFBP2, GFAP, EIF4EBP1, PAPSS2, SLC14A1, IFITM2, EMP2, PNP, TIFA, DAPP1, NOTCH4, VNN1, TNFRSF1A, IL18, ANGPTL4, ARID5A, CEBPD
Ctx.Cort : OXTR, PTGS1, NNMT, MAFB
Ctx.Cnp : ITGB4, ST6GAL1, ADAMTSL4, FOXJ1, ARRDC3, ELOVL7, TMPRSS5, PDLIM1, NFKBIA, LCP1, RASL12, CLDN14, RPS6KA1, CTSC, EFNA1, MTHFD2
Epi : TM4SF1, ANGPT2, TAL1, C2CD4B, FCER1G, C1QC, CTXN3, RAMP3, GPR4, IFITM3, RASD1, TIFA, ST14
Epi.Chat : PSMB9, PSMB8, GPR4, CXCL11, C1QA, NFAM1, WFIKKN2, IRX3
Spc : CALCB, ARRDC3, PDLIM1, VWF, ABCC4, PRX, CSF1R, ADAMTSL4, PTPRC, ART3, TMEM119, C1QB, TGM2, S100A8, CLDN14, BACE2, ANXA2, CTSS, TYROBP, ITGB4, S100A9, GFAP, EMP1, TMPRSS5, GBP6, RBMS2, LY86, SELPLG, SRGN, APLNR, CD44, ELOVL7, CSF1, IGFBP3, ADAMTS9, TGFB3, SERPING1, RASSF4, XIST, C3
Spc.Chat : CALCB, TUBB6, TNFRSF12A, TNFRSF1A, CDA, AGTRAP, TRIP10, CFI, LCP1, TSPO, PIK3R5, LGI2, RASSF4, IRX3, HYAL2, ANXA2, MTHFD2
RetC.Cones : SAMSN1, MFNG, SLC1A7, TM4SF1, VWF, SOCS3, PIK3AP1, CFH, STXBP2, RGR, PTP4A3, LYVE1, CTSC, SLC14A1, MGP, IER3, NPL, ABI3, HK2, SLC7A5, VASP, LHFPL2, PRAM1, C1QA, SERPING1, ARHGDIB, GPSM3, CEBPD, XIST, CEBPB

The overlapped genes for every cell type (pSI threshold 0.01 **) are:**
RetR.Rods : SLC16A3, ALPL, AQP1, SLCO4A1, MYO7A, SEMA3F, SLC26A2, TNFAIP3, CP, SUSD3, ROM1
Hyp : OXT, AVP, SYTL4, SELPLG, RASD1, RNASE6
Hyp.Hcrt : IGFBP3, C2CD4B, IGHG1, TNFRSF12A, CASP1
BF : IFITM3, OXT, MGP, TAGLN, AVP
BF.Chat : IL1R2, RGS10, LST1, IGHG1
BS : LY86, CALCB, TAL1
BS.Chat : CALCB, CLIC1, TAGLN2, TNFRSF12A, CDA, LCP1, ANXA2
BS.Slc6a4 : GPR4
Cb : SLA, RPS6KA1, CNKSR3, MYBPC1
Cb.Septin4 : CHI3L1, RHOD, SLC14A1, PLSCR1, HK2, CEBPD, EMP2, ANGPTL4, SLCO4A1, CNN2, PXN, UCP2, CDKN1A, CASP1, GLI1, ARID5A, DENND3, CYBA, TIMP1, MYBPC1
Cb.Pcp2 : RHOD, ALOX5AP, RTEL1, CCDC69
Cb.Neurod1 : RPS6KA1, CNKSR3, UNC13D, BCL6
Cb.Lypd6 : ST14, SLA, TIFA, TMEM204
Cb.Grp : CTXN3, HK2, ANGPTL4, CXCL11, UCP2, SERPING1, CLEC1A
Cb.Grm2 : ST14, LGI2
Cb.Fthfd : CHI3L1, GSTT1, RHOD, VAMP8, HK2, IFITM2, EMP2, SLCO4A1, CNN2, SERPINH1, RRM2, CTXN3, LY96, DENND3, CYBA, SLC14A1
Cb.Cnp : ITGB4, ST6GAL1, ADAMTSL4, TMPRSS5, ELOVL7, CSF1, ARRDC3, PDLIM1, RASL12, CLDN14, BACE2, CTSC, EFNA1
Cpu : IDO1, INHBA, ARHGDIB
Cpu.D2 : IDO1, CISH, ARHGDIB, ACVRL1, STC1
Cpu.D1 : IDO1, AIF1, ACVRL1, ARHGDIB
Cpu.Chat : LST1, CD44, CDA, IGHG1
Ctx : KLF10, STARD8
Ctx.Pdgfrajd340 : TAGLN2, ASF1B, CNN2, RRM2, IGFBP3, MELK, RASL12, AURKB, TOP2A, CDK2, BCL6B
Ctx.Etv1_ts88 : PTPN6, TYROBP, CYTH4, ITGB2, CFH, PTGS1, FERMT3, STAB1, IGFBP2, CYBA, CD14, LYVE1, FCER1G, F13A1, STARD8, CD163, SLC11A1, CD68, CSF1R, LAPTM5, IFITM1, TNFAIP8L2, C1QC, C1QB, C1QA, MS4A7
Ctx.Pnoc : NNMT, ZBTB16
Ctx.Ntsr : none
Ctx.Glt25d2 : none
Ctx.Fthfd : FGFRL1, CHI3L1, SLC7A2, NUPR1, GFAP, TNFRSF1A, EMP2, ANGPTL4, IGFBP2, NOTCH4, IFITM2, ID3, TIFA, ARID5A, PAPSS2, DAPP1
Ctx.Cort : NNMT, MAFB
Ctx.Cnp : ITGB4, ADAMTSL4, ARRDC3, FOXJ1, ELOVL7, TMPRSS5, PDLIM1, RASL12
Epi : RASD1, TM4SF1, CTXN3, GPR4, RAMP3
Epi.Chat : PSMB9, PSMB8, GPR4, CXCL11, NFAM1, WFIKKN2, IRX3
Spc : S100A9, CSF1R, TYROBP, RASSF4, PTPRC, GFAP, EMP1, TMPRSS5, CALCB, ART3, TMEM119, VWF, LY86, CTSS, S100A8, PRX, ADAMTS9, APLNR, ELOVL7, C3
Spc.Chat : CALCB, CDA, AGTRAP, CFI, LCP1, TUBB6, PIK3R5, ANXA2, IRX3
RetC.Cones : SAMSN1, LYVE1, SLC1A7, HK2, RGR, CFH, SOCS3, PRAM1, MFNG, SERPING1, ARHGDIB, GPSM3, ABI3, XIST, PIK3AP1, CEBPB

The overlapped genes for every cell type (pSI threshold 0.001 **) are:**
RetR.Rods : SLC16A3, ALPL, AQP1, MYO7A, SEMA3F, TNFAIP3, CP, SUSD3, ROM1
Hyp : RNASE6, OXT, AVP, SYTL4
Hyp.Hcrt : IGFBP3, C2CD4B, IGHG1, CASP1
BF : TAGLN
BF.Chat : none
BS : TAL1
BS.Chat : CALCB, CDA, LCP1, ANXA2
BS.Slc6a4 : GPR4
Cb : SLA, RPS6KA1
Cb.Septin4 : CHI3L1, SLC14A1, HK2, ANGPTL4, CNN2, UCP2, GLI1, DENND3, CEBPD, MYBPC1
Cb.Pcp2 : RHOD, CCDC69
Cb.Neurod1 : RPS6KA1, CNKSR3
Cb.Lypd6 : ST14, SLA, TMEM204
Cb.Grp : CTXN3, CLEC1A, SERPING1
Cb.Grm2 : ST14, LGI2
Cb.Fthfd : GSTT1, CTXN3, SLC14A1, SLCO4A1
Cb.Cnp : ITGB4, ADAMTSL4, TMPRSS5, PDLIM1, RASL12, CTSC
Cpu : INHBA
Cpu.D2 : IDO1, ACVRL1, ARHGDIB
Cpu.D1 : IDO1
Cpu.Chat : CD44
Ctx : none
Ctx.Pdgfrajd340 : TAGLN2, RRM2, MELK, RASL12, AURKB, TOP2A, CDK2, BCL6B
Ctx.Etv1_ts88 : TYROBP, PTPN6, F13A1, CSF1R, FERMT3, STAB1, CYTH4, C1QC, C1QB, C1QA, CD163, CD14, LYVE1, MS4A7
Ctx.Pnoc : ZBTB16
Ctx.Ntsr : none
Ctx.Glt25d2 : none
Ctx.Fthfd : PAPSS2, NUPR1, GFAP, EMP2, ANGPTL4, ID3, TIFA, ARID5A, NOTCH4
Ctx.Cort : none
Ctx.Cnp : ITGB4, ELOVL7, ARRDC3, ADAMTSL4
Epi : RASD1, RAMP3
Epi.Chat : PSMB9, GPR4, CXCL11, NFAM1, WFIKKN2, IRX3
Spc : PTPRC, S100A8, EMP1, TMPRSS5, GFAP, LY86, APLNR, PRX, C3
Spc.Chat : CALCB, LCP1, CFI, ANXA2
RetC.Cones : SAMSN1, MFNG, SLC1A7, HK2, RGR, SOCS3, ARHGDIB, XIST, PIK3AP1, GPSM3

The overlapped genes for every cell type (pSI threshold 0.0001 **) are:**
RetR.Rods : ALPL, AQP1, SEMA3F, TNFAIP3, SUSD3, ROM1
Hyp : OXT, AVP, SYTL4
Hyp.Hcrt : C2CD4B, IGHG1
BF : TAGLN
BF.Chat : none
BS : none
BS.Chat : CALCB, CDA, LCP1, ANXA2
BS.Slc6a4 : none
Cb : none
Cb.Septin4 : SLC14A1, HK2, CNN2, GLI1, UCP2, MYBPC1
Cb.Pcp2 : none
Cb.Neurod1 : RPS6KA1
Cb.Lypd6 : ST14, SLA, TMEM204
Cb.Grp : CTXN3, SERPING1
Cb.Grm2 : ST14
Cb.Fthfd : SLC14A1
Cb.Cnp : TMPRSS5, CTSC, ADAMTSL4, ITGB4
Cpu : none
Cpu.D2 : IDO1
Cpu.D1 : IDO1
Cpu.Chat : none
Ctx : none
Ctx.Pdgfrajd340 : RRM2, MELK, TOP2A, TAGLN2, BCL6B
Ctx.Etv1_ts88 : TYROBP, PTPN6, F13A1, CSF1R, STAB1, CYTH4, C1QC, C1QB, C1QA, CD163, CD14, MS4A7
Ctx.Pnoc : none
Ctx.Ntsr : none
Ctx.Glt25d2 : none
Ctx.Fthfd : EMP2, NUPR1, PAPSS2, ID3, ARID5A, TIFA
Ctx.Cort : none
Ctx.Cnp : ITGB4, ADAMTSL4
Epi : RASD1, RAMP3
Epi.Chat : NFAM1, GPR4, CXCL11, WFIKKN2
Spc : S100A8, PRX, C3
Spc.Chat : CALCB, LCP1, CFI, ANXA2
RetC.Cones : SAMSN1, MFNG, SLC1A7, RGR, XIST, PIK3AP1
