# Supplementary material for: Molecular Landscape of Tourette’s Disorder
Source: Int J Mol Sci. 2023 Jan 11;24(2):1428. doi: 10.3390/ijms24021428 (PMC9865021; doi:10.3390/ijms24021428)
Supplement: Supplementary file 1 [file ijms-24-01428-s001.zip › supplementary File S7.pdf]

Of the inputed 362 genes,
266 genes exist in the brain cell type expression dataset, 96 genes do not, which are:
CHI3L2,S100A11,C1RL,NAPSB,LYZ,SP110,NABP1,HLA-
DPB1,RNASE3,RNASE2,CLEC17A,TRIM22,SIGLEC10,SERPINB1,MS4A4A,SLC66A3,SYK,ARPC1B,SECTM1,EFL1,ADGRG5,TLR5,CD300C,CD300E,SP140,C2ORF73,LILRB4,LILRB2,LILRB1,HCP5,C1ORF54,C19ORF38,FCN1,TFEC,VNN2,GBP5,GPR141,C17ORF64,TAGAP,BCL2A1,DHRS9,SIRPB2,MS4A14,NCF2,ACP3,TLR10,PTAFR,RNASET2,NIBAN1,SPI1,TASL,CEACAM21,IFI16,TNFRSF6B,C1ORF162,C5ORF58,C16ORF54,TMEM156,APOBEC3C,APOBEC3B,PSMB8-
AS1,HLA-DOA,HLA-DPA1,LPAR5,FCGR3A,HILPDA,ADGRE2,ARL11,LRRC25,RPL30,NLRC4,BIN2,SERPINA1,HLA-
DRA,SERPINA3,TMIGD2,MNDA,APOL4,GNGT2,PHYKPL,TLDC2,FCGR2A,MS4A6A,C1R,SIGLEC7,OTULINL,LILRA2,NAMPTP1,TYMP,SIGLEC8,SIGLEC9,PCLAF,LILRA5,HLA-DMB,HLA-DQA2,HLA-DMA

Interpretation of CSEA results table:
The first row lists the pSI thresholds, and the first column lists the brain cell types
The values in the tables are the Fisher's Exact p-values, followed by the Benjamini-Hochberg (BH) corrected values in parentheses.
Click the linked p-values to see the overlapped genes for the corresponding brain cell types and thresholds.

Brain Cell Types and P-Values	0.05	0.01	0.001	0.0001
RetR.Rods	0.668 (0.982)	0.662 (1.000)	0.570 (1.000)	0.412 (1.000)
Hyp	0.864 (0.982)	0.334 (1.000)	0.394 (1.000)	1.000 (1.000)
Hyp.Hcrt	0.772 (0.982)	0.917 (1.000)	0.718 (1.000)	1.000 (1.000)
BF	0.481 (0.982)	1.000 (1.000)	1.000 (1.000)	1.000 (1.000)
BF.Chat	0.776 (0.982)	0.535 (1.000)	1.000 (1.000)	1.000 (1.000)
BS	0.178 (0.982)	0.181 (1.000)	0.131 (0.790)	1.000 (1.000)
BS.Chat	0.279 (0.982)	0.329 (1.000)	0.755 (1.000)	0.552 (1.000)
BS.Slc6a4	0.912 (0.982)	1.000 (1.000)	1.000 (1.000)	1.000 (1.000)
Cb	0.977 (0.982)	0.733 (1.000)	0.181 (0.790)	1.000 (1.000)
Cb.Septin4	0.585 (0.982)	0.316 (1.000)	0.362 (1.000)	0.298 (1.000)
Cb.Pcp2	0.854 (0.982)	0.887 (1.000)	1.000 (1.000)	1.000 (1.000)
Cb.Neurod1	0.978 (0.982)	0.755 (1.000)	0.560 (1.000)	0.303 (1.000)
Cb.Lypd6	0.784 (0.982)	0.614 (1.000)	0.261 (1.000)	0.166 (1.000)
Cb.Grp	0.153 (0.982)	0.505 (1.000)	1.000 (1.000)	1.000 (1.000)
Cb.Grm2	0.973 (0.982)	0.547 (1.000)	1.000 (1.000)	1.000 (1.000)
Cb.Fthfd	0.714 (0.982)	0.551 (1.000)	1.000 (1.000)	1.000 (1.000)
Cb.Cnp	0.867 (0.982)	0.903 (1.000)	0.971 (1.000)	0.876 (1.000)
Cpu	0.586 (0.982)	0.700 (1.000)	1.000 (1.000)	1.000 (1.000)
Cpu.D2	0.981 (0.982)	0.647 (1.000)	0.370 (1.000)	0.473 (1.000)
Cpu.D1	0.977 (0.982)	0.670 (1.000)	1.000 (1.000)	1.000 (1.000)
Cpu.Chat	0.878 (0.982)	0.874 (1.000)	1.000 (1.000)	1.000 (1.000)
Ctx	0.808 (0.982)	0.524 (1.000)	1.000 (1.000)	1.000 (1.000)
Ctx.Pdgfrajd340	0.708 (0.982)	0.143 (1.000)	0.152 (0.790)	0.290 (1.000)
Ctx.Etv1_ts88	6.988e-12 (2.446e-10)	2.753e-14 (9.637e-13)	3.563e-10 (1.247e-08)	8.173e-11 (2.861e-09)
Ctx.Pnoc	0.951 (0.982)	1.000 (1.000)	1.000 (1.000)	1.000 (1.000)
Ctx.Ntsr	0.889 (0.982)	0.370 (1.000)	0.026 (0.308)	0.113 (0.990)
Ctx.Glt25d2	0.385 (0.982)	1.000 (1.000)	1.000 (1.000)	1.000 (1.000)
Ctx.Fthfd	0.434 (0.982)	0.458 (1.000)	1.000 (1.000)	1.000 (1.000)
Ctx.Cort	0.902 (0.982)	0.675 (1.000)	1.000 (1.000)	1.000 (1.000)
Ctx.Cnp	0.982 (0.982)	0.998 (1.000)	1.000 (1.000)	1.000 (1.000)
Epi	0.831 (0.982)	1.000 (1.000)	1.000 (1.000)	1.000 (1.000)
Epi.Chat	0.898 (0.982)	0.441 (1.000)	0.346 (1.000)	0.755 (1.000)
Spc	0.275 (0.982)	0.013 (0.220)	0.025 (0.308)	0.106 (0.990)
Spc.Chat	0.847 (0.982)	0.351 (1.000)	0.176 (0.790)	0.034 (0.595)
RetC.Cones	0.549 (0.982)	0.199 (1.000)	0.063 (0.554)	0.478 (1.000)

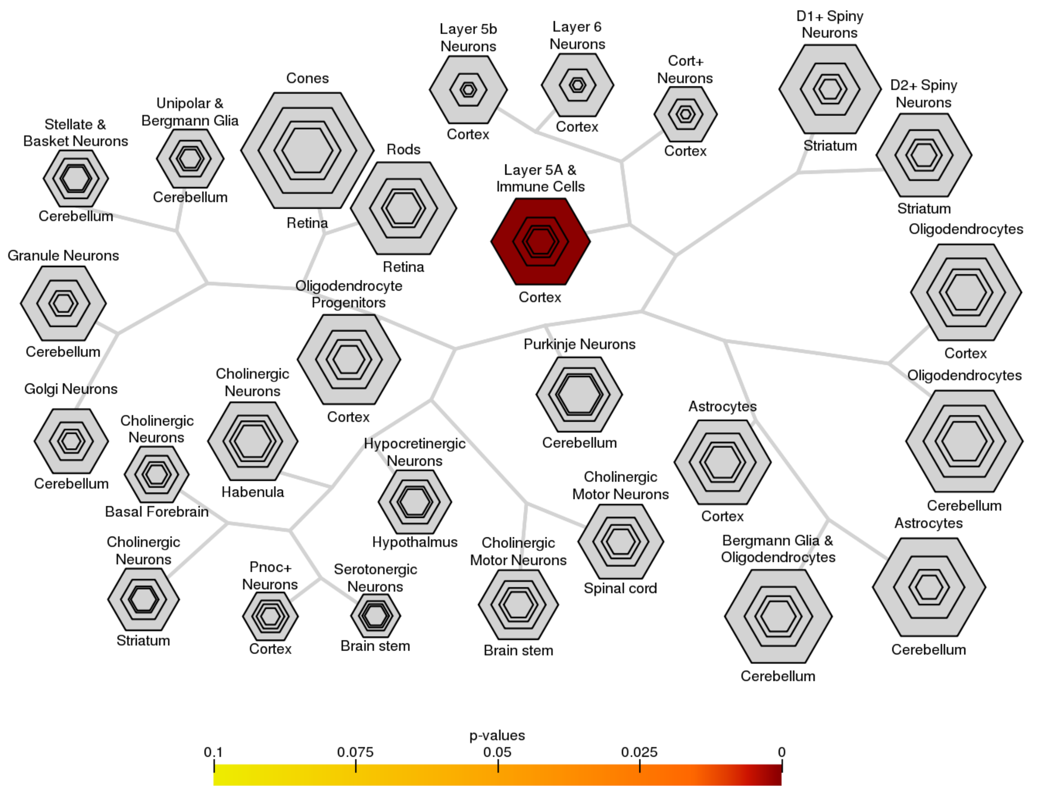

The overlapped genes for every cell type (pSI threshold 0.05 **) are:**
RetR.Rods : B4GALT1, EHD4, CXCL16, MYD88, RTEL1, SLC26A2, TNFAIP3, HCLS1, SUSD3, RHBDF2
Hyp : C1QC, SELPLG, RNASE6
Hyp.Hcrt : SLPI, PIK3R5, UCP2, CASP1
BF : LY86, FCER1G, C1QA, CD163, CD68
BF.Chat : SLPI, RGS10, LST1
BS : FCER1G, OLFML3, TAL1, IGFBP2, C1QB, LY86, C3
BS.Chat : CLIC1, BIRC5, CSRP2, EMB, TNFSF13B, LCP1, PIK3R5, C3
BS.Slc6a4 : LST1
Cb : SLA, RPS6KA1, RTEL1, PIK3R3, LYN
Cb.Septin4 : B2M, CHI3L1, B4GALT1, VAMP8, CMTM3, CLIC1, CASP1, HK2, CYBA, UCP2, HCK
Cb.Pcp2 : ARHGAP15, ALOX5AP, RTEL1, LCP1, F11R
Cb.Neurod1 : RPS6KA1, UNC13D, VAMP8
Cb.Lypd6 : CMTM7, SLA, PLVAP
Cb.Grp : CHI3L1, VAMP8, CMTM3, CLIC1, CASP1, HK2, UCP2
Cb.Grm2 : PRRG4, F11R
Cb.Fthfd : B2M, CHI3L1, PTGS1, VAMP8, CMTM3, LHFPL2, HK2, CYBA, UCP2, IFI30, CASP1
Cb.Cnp : SLPI, LAP3, ST6GAL1, PSTPIP2, IL18, LHFPL2, ARHGAP15, RPS6KA1, CTSC, MTHFD2
Cpu : SELPLG, AIF1, C1QB, C1QA, ARHGDIB
Cpu.D2 : CD4, CISH, AIF1, ARHGDIB
Cpu.D1 : CD4, CISH, AIF1, ARHGDIB, CD68
Cpu.Chat : RGS10, LST1, CASP1
Ctx : ARHGAP15, ARHGAP25, BLNK
Ctx.Pdgfrajd340 : RAB32, ST6GAL1, CMTM3, ASF1B, NUSAP1, MYD88, CDK1, CDK2, ETV6
Ctx.Etv1_ts88 : PTPN6, BLNK, AIF1, CYTH4, TBXAS1, ITGB2, IRF8, ADAP2, PTGS1, GMFG, HK3, FERMT3, STAB1, IGFBP2, CYBA, CTSS, NCKAP1L, CD14, LYVE1, CTSC, RAB32, FCER1G, F13A1, INPP5D, CD163, LY86, SLC11A1, CD68,
CSF1R, LAPTM5, TNFAIP8L2, C1QC, C1QB, C1QA, ARHGDIB, MS4A7
Ctx.Pnoc : LST1
Ctx.Ntsr : CSF1R, ARHGAP25, BLNK
Ctx.Glt25d2 : BLNK, ARHGAP25, CSF1R, GBP2, ITGB2, TNFAIP3, LRG1
Ctx.Fthfd : CHI3L1, VAMP8, CMTM3, MYD88, IL18, PNP, IGFBP2, ABCC4, VNN1, DAPP1
Ctx.Cort : PTGS1, MAFB
Ctx.Cnp : LAP3, ST6GAL1, LCP1, RPS6KA1, CTSC, MTHFD2
Epi : CMTM7, C1QC, FCER1G, TAL1
Epi.Chat : NFAM1, PSMB8, DOCK2, C1QA, PSMB9
Spc : S100A9, OLFML3, PTPRC, S100A8, CSF1R, TNFSF13B, TMEM119, C1QB, CTSS, LY86, SELPLG, ABCC4, C3
Spc.Chat : TNFSF13B, PIK3R5, LCP1, CFI, MTHFD2
RetC.Cones : MALT1, SAMSN1, LYVE1, NAGA, OLFML3, SLC1A7, HK2, LMNB1, NFKB1, LHFPL2, PRAM1, C1QA, ARHGDIB, ABI3, CTSC, PIK3AP1, GPSM3

The overlapped genes for every cell type (pSI threshold 0.01 **) are:**
RetR.Rods : SUSD3, B4GALT1, SLC26A2, TNFAIP3
Hyp : SELPLG, RNASE6
Hyp.Hcrt : CASP1
BF : none
BF.Chat : RGS10, LST1
BS : LY86, TAL1
BS.Chat : TNFSF13B, LCP1, CLIC1, CSRP2
BS.Slc6a4 : none
Cb : SLA, RPS6KA1
Cb.Septin4 : CHI3L1, CMTM3, CASP1, HK2, CYBA, UCP2
Cb.Pcp2 : ALOX5AP, RTEL1
Cb.Neurod1 : RPS6KA1, UNC13D
Cb.Lypd6 : CMTM7, SLA
Cb.Grp : HK2, UCP2
Cb.Grm2 : PRRG4, F11R
Cb.Fthfd : CMTM3, CHI3L1, CYBA, HK2, VAMP8
Cb.Cnp : LAP3, ARHGAP15, ST6GAL1, CTSC
Cpu : ARHGDIB
Cpu.D2 : CD4, CISH, ARHGDIB
Cpu.D1 : CD4, AIF1, ARHGDIB
Cpu.Chat : LST1
Ctx : ARHGAP25
Ctx.Pdgfrajd340 : RAB32, CMTM3, ASF1B, NUSAP1, CDK1, CDK2, ETV6
Ctx.Etv1_ts88 : PTPN6, CYTH4, ITGB2, PTGS1, FERMT3, STAB1, IGFBP2, CYBA, CD14, LYVE1, RAB32, FCER1G, F13A1, CD163, SLC11A1, CD68, CSF1R, LAPTM5, TNFAIP8L2, C1QC, C1QB, C1QA, MS4A7
Ctx.Pnoc : none
Ctx.Ntsr : ARHGAP25, BLNK
Ctx.Glt25d2 : none
Ctx.Fthfd : CMTM3, CHI3L1, IGFBP2, MYD88, DAPP1
Ctx.Cort : MAFB
Ctx.Cnp : LAP3
Epi : none
Epi.Chat : NFAM1, PSMB9, DOCK2, PSMB8
Spc : S100A9, PTPRC, S100A8, CSF1R, TMEM119, CTSS, LY86, C3
Spc.Chat : TNFSF13B, PIK3R5, LCP1, CFI
RetC.Cones : MALT1, SAMSN1, LYVE1, SLC1A7, HK2, NFKB1, LMNB1, PRAM1, ARHGDIB, ABI3, PIK3AP1, GPSM3

The overlapped genes for every cell type (pSI threshold 0.001 **) are:**
RetR.Rods : SUSD3, TNFAIP3
Hyp : RNASE6
Hyp.Hcrt : CASP1
BF : none
BF.Chat : none
BS : TAL1
BS.Chat : LCP1
BS.Slc6a4 : none
Cb : SLA, RPS6KA1
Cb.Septin4 : HK2, CHI3L1, UCP2
Cb.Pcp2 : none
Cb.Neurod1 : RPS6KA1
Cb.Lypd6 : SLA, CMTM7
Cb.Grp : none
Cb.Grm2 : none
Cb.Fthfd : none
Cb.Cnp : CTSC
Cpu : none
Cpu.D2 : CD4, ARHGDIB
Cpu.D1 : none
Cpu.Chat : none
Ctx : none
Ctx.Pdgfrajd340 : RAB32, NUSAP1, CDK1, CDK2
Ctx.Etv1_ts88 : PTPN6, F13A1, CSF1R, FERMT3, STAB1, CYTH4, C1QC, C1QB, C1QA, CD163, CD14, LYVE1, MS4A7
Ctx.Pnoc : none
Ctx.Ntsr : ARHGAP25, BLNK
Ctx.Glt25d2 : none
Ctx.Fthfd : none
Ctx.Cort : none
Ctx.Cnp : none
Epi : none
Epi.Chat : PSMB9, NFAM1, DOCK2
Spc : PTPRC, S100A8, LY86, C3
Spc.Chat : TNFSF13B, LCP1, CFI
RetC.Cones : MALT1, SAMSN1, SLC1A7, HK2, LMNB1, ARHGDIB, PIK3AP1, GPSM3

The overlapped genes for every cell type (pSI threshold 0.0001 **) are:**
RetR.Rods : SUSD3, TNFAIP3
Hyp : none
Hyp.Hcrt : none
BF : none
BF.Chat : none
BS : none
BS.Chat : LCP1
BS.Slc6a4 : none
Cb : none
Cb.Septin4 : HK2, UCP2
Cb.Pcp2 : none
Cb.Neurod1 : RPS6KA1
Cb.Lypd6 : SLA, CMTM7
Cb.Grp : none
Cb.Grm2 : none
Cb.Fthfd : none
Cb.Cnp : CTSC
Cpu : none
Cpu.D2 : CD4
Cpu.D1 : none
Cpu.Chat : none
Ctx : none
Ctx.Pdgfrajd340 : RAB32, NUSAP1
Ctx.Etv1_ts88 : PTPN6, F13A1, CSF1R, STAB1, CYTH4, C1QC, C1QB, C1QA, CD163, CD14, MS4A7
Ctx.Pnoc : none
Ctx.Ntsr : ARHGAP25
Ctx.Glt25d2 : none
Ctx.Fthfd : none
Ctx.Cort : none
Ctx.Cnp : none
Epi : none
Epi.Chat : NFAM1
Spc : S100A8, C3
Spc.Chat : TNFSF13B, LCP1, CFI
RetC.Cones : SAMSN1, PIK3AP1, SLC1A7
