# Supplementary material for: Molecular Landscape of Tourette’s Disorder
Source: Int J Mol Sci. 2023 Jan 11;24(2):1428. doi: 10.3390/ijms24021428 (PMC9865021; doi:10.3390/ijms24021428)
Supplement: Supplementary file 1 [file ijms-24-01428-s001.zip › supplementary Figure S2.pdf]

Endocannabinoids regulate synaptic function through retrograde signaling, autocrine signaling, and also indirectly by activating astrocytic receptors.

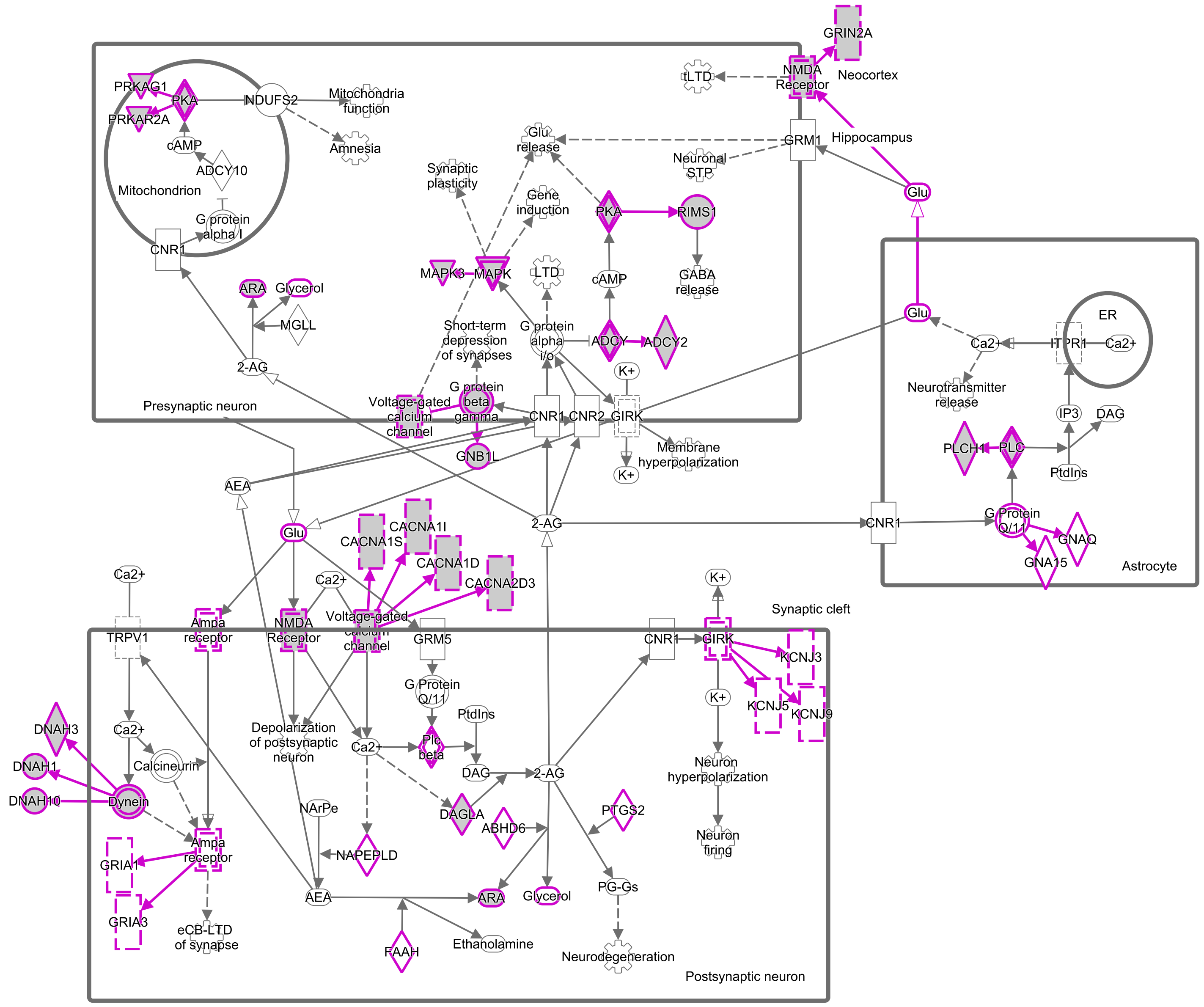

© 2000-2022 QIAGEN. All rights reserved.

Figure S2. Endocannabinoid Neuronal Synapse Pathway identified by IPA
The Endocannabinoid Neuronal Synapse Pathway was identified as the second most significantly enriched canonical pathway in the set of 872 TD candidate genes (FDR P-value <0.05). The diagram represents the Endocannabinoid Neuronal Synapse Pathway at the cellular level. It depicts the genes/proteins and metabolites involved, their interactions, and the cellular and metabolic reactions in which this pathway is involved. Molecules are represented as nodes, and the biological relationship between two nodes is represented as an edge (line). Molecules highlighted with pink-outline and gray color represent genes/proteins/protein complexes which members appear in the set of 872 TD candidate genes and metabolites which showed shared genetic etiology with TD in the PRS-based analyses at Bonferroni adjusted P-value <0.05. Pink-outlined molecules without a color indicate metabolites which showed shared genetic etiology with TD at FDR P-value < 0.01 or genes that were differentially expressed in the blood or brain tissue of TD patients. Double-bordered molecules represent groups or complexes. Pink arrows indicate members of a complex that are present in the set of 872 TD candidate genes. Abbreviations: ARA – arachidonic acid; 2-AG – 2-arachidonoylglycerol; AEA – anandamine; NArPe – N-arachidonylphosphatidylethanolamine; cAMP – cyclic AMP; Glu – glutamate, DAG – diacylglycerol; LTD – Long-term synaptic depression; tLTD – Timing-dependent long-term depression; eCB-LTD of synapse – Endocannabinoid-dependent long-term depression of synapse; Neuronal STP – short-term potentiation of neurons; PtdIns - phosphatidylinositol
