# Supplementary material for: Molecular Landscape of Tourette’s Disorder
Source: Int J Mol Sci. 2023 Jan 11;24(2):1428. doi: 10.3390/ijms24021428 (PMC9865021; doi:10.3390/ijms24021428)
Supplement: Supplementary file 1 [file ijms-24-01428-s001.zip › supplementary File S1.pdf]

Of the inputed 872 genes,
776 genes exist in the tissue expression dataset, 96 genes do not, which are:
TLCD4,NELFA,HEXA-AS1,URAD,NKX2-2,GPR75-ASB3,NAPSB,TMEM191A,DGCR5,P3H2,UFD1,TMEM178B,EIF4EBP2P3,KMT5A,TRIQK,LINC02694,SNU13,KIRREL1,HNRNPLL,MARCHF7,TLNRD1,VPS9D1-AS1,SAXO2,EFL1,MPIG6B,OFCC1,ASS1P2,SNORC,COPS9,CORO7-PAM16,ICE1,GFUS,TANGO2,RTL10,LY75-CD302,RNA5SP172,MTRFR,ST20-MTHFS,POM121L4P,RAMAC,KAT14,ARIH2OS,PALM2AKAP2,ATP5IF1,SNORA79,IHO1,CEP295NL,LRATD1,CFAP99,STING1,NSD3,RNU6-1228P,HYKK,TENM2,C20ORF204,TENM4,P2RX6P,DYNC2I1,PROB1,CIBAR1,ESS2,SLC49A3,CFAP251,DNAJB7,GP1BB,MRTFA,BOLA3P2,OR8T1P,PI4KAP1,NOTCH2NLA,TSSK2,CARF,LY6G5B,MIR2113,SEPTIN5,ST20-AS1,THEM7P,INSYN1,MARF1,DESI1,MICU3,MINAR1,SELENOK,BCDIN3D-AS1,COP1,DTX2P1,SELENOF,KMT2C,COA8,KMT2D,GPR42,MESD,DOP1B,DGCR11,DCAF1,KARS1

Interpretation of TSEA results table:
The first row lists the pSI thresholds, and the first column lists the tissues
The values in the tables are the Fisher's Exact p-values, followed by the Benjamini-Hochberg (BH) corrected values in parentheses.
The overlapped genes for the corresponding tissues and thresholds are listed at the end.

Tissues and P-Values	0.05	0.01	0.001	0.0001
Adipose Tissue	0.865 (1.0)	0.946 (0.995)	1.0 (1.0)	1.0 (1.0)
Adrenal Gland	0.419 (0.953)	0.185 (0.715)	0.296 (1.0)	0.324 (1.0)
Blood	1.0 (1.0)	0.968 (0.995)	0.718 (1.0)	1.0 (1.0)
Blood Vessel	0.283 (0.785)	0.774 (0.995)	0.324 (1.0)	1.0 (1.0)
Brain	3.669e-10 (9.173e-09)	1.091e-06 (2.727e-05)	0.017 (0.433)	0.041 (1.0)
Breast	0.486 (1.0)	0.986 (0.995)	1.0 (1.0)	1.0 (1.0)
Colon	0.859 (1.0)	0.749 (0.995)	0.554 (1.0)	0.462 (1.0)
Esophagus	0.925 (1.0)	0.972 (0.995)	1.0 (1.0)	1.0 (1.0)
Fallopian Tube	0.912 (1.0)	0.554 (0.995)	1.0 (1.0)	1.0 (1.0)
Heart	0.12 (0.579)	0.16 (0.715)	0.795 (1.0)	1.0 (1.0)
Kidney	0.795 (1.0)	0.962 (0.995)	0.992 (1.0)	1.0 (1.0)
Liver	0.908 (1.0)	0.891 (0.995)	0.917 (1.0)	1.0 (1.0)
Lung	0.816 (1.0)	0.377 (0.944)	0.671 (1.0)	0.292 (1.0)
Muscle	0.162 (0.579)	0.125 (0.715)	0.481 (1.0)	0.435 (1.0)
Nerve	0.064 (0.397)	0.222 (0.715)	0.94 (1.0)	1.0 (1.0)
Ovary	0.146 (0.579)	0.174 (0.715)	0.744 (1.0)	1.0 (1.0)
Pancreas	0.208 (0.649)	0.337 (0.936)	0.281 (1.0)	1.0 (1.0)
Pituitary	1.041e-06 (1.301e-05)	0.001 (0.013)	0.157 (1.0)	1.0 (1.0)
Prostate	0.885 (1.0)	0.918 (0.995)	1.0 (1.0)	1.0 (1.0)
Skin	0.987 (1.0)	0.995 (0.995)	0.995 (1.0)	0.989 (1.0)
Stomach	0.976 (1.0)	0.985 (0.995)	0.803 (1.0)	1.0 (1.0)
Testis	0.94 (1.0)	0.963 (0.995)	0.995 (1.0)	0.998 (1.0)
Thyroid	0.368 (0.92)	0.528 (0.995)	0.584 (1.0)	0.721 (1.0)
Uterus	0.033 (0.277)	0.229 (0.715)	0.603 (1.0)	1.0 (1.0)
Vagina	0.787 (1.0)	0.92 (0.995)	0.64 (1.0)	1.0 (1.0)

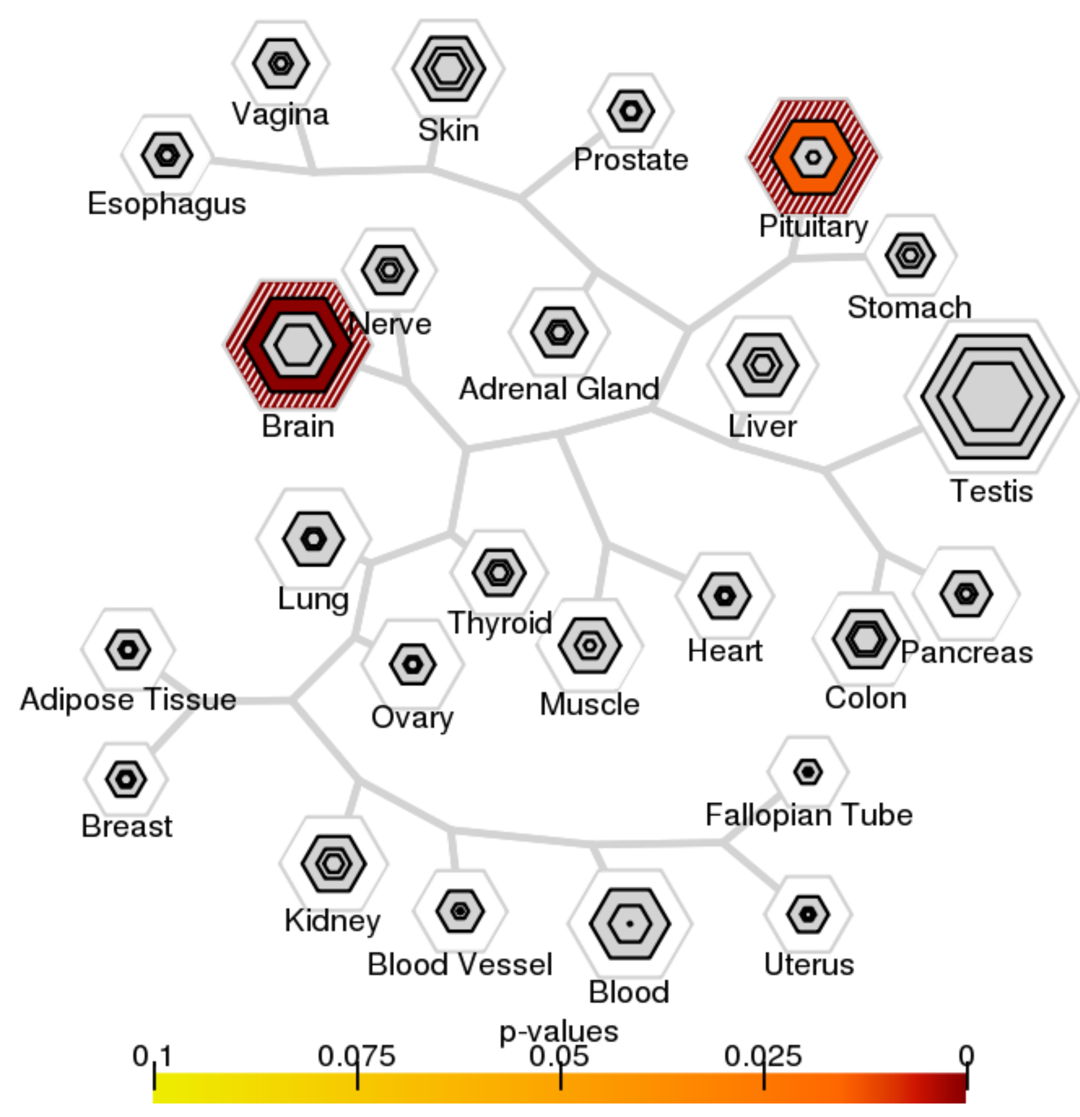

The overlapped genes for every tissue (pSI threshold 0.05 **) are:**
Adipose Tissue : CXCL14, KLB, TLN2, ZNF385D, PLEK, POSTN, TEK, SELE, SLC25A1, NAT8L, COL4A2, CCDC85A, DPP4, DLC1, ADM, MGST1, LAPTM5, HTRA3, CLDN5, FMO2, BST1, ETS1, COL6A3, NEGR1, TNN, FFAR3, MRC1, FPR3, RAB42
Adrenal Gland : CACNA1I, FBN2, NLGN3, CHST6, CHGB, DCAF12, NRCAM, PLEK2, SLC23A2, SLC26A2, SLC10A4, CD276, CSDC2, DNAH11, NXPH1, CHRNB4, FHIT, ARHGEF3, EMB, CNTN6, SLC6A2, VGF, AADAC, UCHL1, DRD2, HS2ST1, MGST1, NAALAD2, SDC3, PPIL1, CCDC134, NGEF, PABPC5, COMT, SLC7A4, P2RX6, SLC27A2, CALN1, CACNA1D, PHOX2B, MPV17, FPR3
Blood : LY75, ARHGAP26, DCAF12, CASP8, PDCD1, PPP1R16B, MEI1, PLEK, MPO, OAS3, BEND4, FFAR3, TMCC2, HRH2, CDC45, LY6G6F, RASGRP1, DPEP2, CSGALNACT2, PREX1, GCA, KCNH7, EMB, LAPTM5, ST20, DOK3, SLCO4C1, TNFRSF13C, EPSTI1, ABCA7, BST1, DYSF, FLT3, SH3BP2, SIGLEC14, FPR1, FPR2, MGAM, RAB44
Blood Vessel : PCDH11X, FN1, LOXL1, ZNF385D, POSTN, PAM, TOB2, PPP1R3B, UNC13C, MPRIP, TIMP2, SORT1, PPP2R2B, SCARF2, HRH2, PCDH7, GEM, KLHL38, METTL24, COL4A2, NEGR1, NR4A2, FOXP1, ASPN, ANKRD34C, NPTN, KALRN, PDLIM7, GPR63, LAMA5, COL8A1, LINGO1, FMO2, CACNA1S, ADAMTS6, NEB, LTBP1, CDKN1A, AEBP1
Brain : CPT1C, CADM2, VWC2, ARHGEF33, CNTNAP2, SLC10A4, OLFM1, NLGN3, ARF3, GRIN2A, DDN, WNT1, WNT10B, HCN1, CCDC85A, KCNK1, SERPINE2, SOWAHA, CAMK1D, ANKRD34C, LMO3, GPRIN1, CHGB, SORCS3, SULT4A1, NSF, PCDH11X, AIFM3, SLITRK1, CNTNAP5, CSMD1, GPR75, SLC39A12, NRXN1, CDHR1, NLGN4X, NIPA1, CKB, ABI2, ZNF382, GPR149, CDH7, PLCH1, MAPT, YIPF7, GPR37, CRHR1, UNC13C, PDE6B, GABRA2, PPP2R2B, SLC4A10, RTN4R, FAM184A, TBR1, GABRB3, YPEL4, MDGA2, CD160, UCHL1, GABRG1, NPAS3, RBFOX1, KLHL32, AP3B2, CPEB1, SLC45A1, LMO4, SYNJ2, KIF26B, FLT3, CALN1, CPNE5, BCL11A, POU3F2, ASTN2, CNTN6, MYT1L, PRR7, ADAM23, C4ORF48, NAT8L, CELSR3, ZFYVE28, SLC24A2, PREPL, COL9A2, CHADL, RASGEF1A, PTPRN2, PREX1, DRD2, DCC, KCNIP4, PKP4, RELN, HCN4, DAGLA, YJEFN3, TNIK, SHANK2, HDC, OPRK1, NRCAM, ABCA2, CSMD3, HS3ST4, RIMBP2, FGF17, SYT17, CACNA1I, AGBL4, DPP6, MCHR1, ADCY2, DLX6, IGIP, PPP1R16B, FAM153B, TMCC2, ERC2, CHST6, MCHR2, ANKRD45, NXPH1, KCNH3, FAXC, PCDH17, RASGRP1, STON2, VGF, CLCA4, KCNC3, ZNF536, CNTNAP4, TEF, TERF2IP, DNAH10, NGEF, CXXC4, LINGO1, CACNA2D3, CADPS2, RIMS1, ABCB9, TUBA1A, ANK3, CMTM5
Breast : LY75, SEMA3E, KLB, BHLHE40, ZNF385D, CAPN6, PDGFRL, SELE, NAT8L, AFF3, SPRY2, PDZK1, CLCA2, ODF2L, IGSF10, ADM, CX3CL1, ETS1, COL6A6, AQP5, ESR1, CLDN5, FMO2, FSIP1, MGST1, TNN, FFAR3, HS3ST4
Colon : LY75, PRSS3, CLCN2, SERINC2, MEI1, RAB19, PLEK2, EPHB2, SLC26A2, PHOX2B, ALDH18A1, GAL3ST2, CHST5, SEL1L3, CDX2, DGKQ, SPINK1, CDC45, GCG, CLCA4, CLCA1, GABRA2, SLC4A10, SOWAHA, DHRS11, PDE9A, SLC23A1, NEO1, EXOC3L4, NGEF, SLC35G1, AIFM3, KCNIP4, SLC27A2, TROAP, PRPH, CDHR1, CKB, DNAJC22
Esophagus : PRSS3, SEMA3E, HOXB4, CDHR1, GPR157, BNC1, CBR3, SPINK5, SPAG17, WNT5A, PCDH7, P2RX2, FAXC, DIO2, ABCC1, CLCA2, CLCA4, DNAH11, MUC6, RIMS1, SLC6A4, GPR37, IGSF3, CADPS2, TUBA1C, FLG
Fallopian Tube : ESR1, HPD, AEBP1, DPP6, CDH23, MCHR1, PCDHB12, TBX1, RASL11A, COL6A3, ASPN, DYNC2H1, LTBP1, COL4A2, CPNE5, PDLIM7, CAPN6, PI16, PDGFRL, COL8A1
Heart : FBN2, ALPK3, HCN4, GTF3A, PDCD1, VWC2, ACO2, PFKM, ADAM23, KBTBD12, CSDC2, NDUFA4, PPP1R3A, PAM, MTIF3, HRH2, TMEM67, GRIN2A, KLHL38, MDH1, CHADL, KCNK1, WDR62, LIMCH1, NDUFA6, HTRA3, RBFOX1, COL6A6, NDUFA13, DGCR6, CTNNA3, CACNA2D3, MTUS2, ANK3, DYSF, SCN5A, CMTM5, KCNJ11, NDUFB1, FHL2, RNF115, PPIL1, MACROD1, OBSCN, TTN, CPNE5
Kidney : CXCL14, SLCO4C1, ITGB6, WWC1, SERINC2, RAB19, CHDH, IMMP2L, PPP1R16B, LRRC43, FHIT, SPINK1, HOXB4, IGF2BP1, DDN, PDZK1, ABCC6, DPP4, KCNC3, GABRA2, FBN3, SOWAHA, NAPSA, PKP4, NDUFA6, SLC23A1, NDUFB1, RAB11FIP3, MACROD1, KCNIP4, HPD, SLC27A2, ACAA1, TRIM50, IL17RB, TLN2, APOM, MGAM, EXOC3L4, C10ORF67, DNAJC22, NCKAP5
Liver : CD302, SPINK1, SERINC2, PLEK2, STARD5, RCAN1, PPP1R3B, WWC1, CDC45, TMBIM6, SESN2, PDZK1, ABCC6, RANGAP1, PNKD, AADAC, STAB2, SLC23A1, RELN, MGST1, GPR37, SLCO4C1, ERRFI1, NGEF, KLB, CPS1, WNT3, SERPIND1, HPD, SLC27A2, ACAA1, IL17RB, CHDH, GPLD1, APOM, ABCG8, SIGLEC14, FPR2, EXOC3L4, DNAJC22, SLC43A1
Lung : ITGB6, FN1, PLEK, CLDN5, TEK, DUSP6, MPO, LIMCH1, THBS1, DNAH10, P2RX2, LY6G6F, OVCH1, DPEP2, IGSF10, FBN3, PCDH12, PREX1, MRC1, NAPSA, DNAH5, CX3CL1, PCDH17, LAPTM5, DOK3, SLCO4C1, COL6A6, SLC6A4, COL8A1, CNTN6, HDC, DNAH3, DLEC1, PCDHAC1, FMO2, DLC1, CACNA1D, MPL, SIGLEC14, FPR1, FPR2, FPR3, C10ORF67, RAB44, PLEKHH2, MGAM, NCKAP5
Muscle : ADSL, PSMD7, ITGB6, MAPT, YIPF7, SCN5A, ACO2, TM6SF1, PFKM, ASB8, CAB39, KBTBD12, KLHL38, C22ORF39, CLTCL1, UNC13C, GTF3A, FBXO10, ST3GAL3, DDN, LRPPRC, DGCR6, NDUFA4, FBXL17, ALPK3, WDR62, PSMD14, LIMCH1, SH3GLB1, PPP1R3A, PRKAR2A, RBFOX1, ABCF2, ADRM1, PRODH, RERE, CTNNA3, CACNA2D3, P2RX6, TBX1, CACNA1S, DYSF, GTF2IRD1, NEB, KCNJ11, PSME4, RNF115, MACROD1, OBSCN, TTN, ADCY2, GPR157
Nerve : CXCL14, NLGN3, CDH7, CADM2, CLCN2, ADAM23, PDGFRL, PPP2R2B, NR4A2, ZDHHC8, SLITRK1, FMNL3, THBS1, RUNX1T1, PRODH, RHEBL1, HCN1, CHADL, RASGEF1A, ZNF536, GABRA2, GABRG1, DRD2, PDE9A, RELN, PTPRU, SDC3, POU3F2, SORCS3, CPEB1, COL8A1, SPRY2, CTNNA3, ARVCF, PI16, STARD9, SCN5A, ABCA2, NEGR1, TUBA1A, TJP1, NRXN1, NLGN4X, OLFM1, CMTM5
Ovary : SLC25A17, NINL, SLC23A2, VWC2, MORF4L2, EPHB2, NR4A2, ZNF581, PDGFRL, CSDC2, ROBO3, FAM153B, SLC29A3, TSPYL5, TIMP2, PITPNM2, WNT5A, ERC2, GABRG1, YPEL4, PCDH11X, SESN2, RBM12B, ZNF83, ST13, IGSF10, SERPINE2, ZNF577, MCHR1, HTRA3, RPS21, NPAS3, SORCS3, SULT4A1, ATN1, ALDH7A1, JUND, PABPC5, PCDHGA2, SGCZ, TMEM200B, CDH23, MPL, MATN1, FHL2, MEX3B, NLGN4X, PLEKHH2, C12ORF54
Pancreas : PRSS3, KCNMB3, ICA1, GCG, NFE2L3, KLB, WWC1, NRCAM, RAB19, SLC43A1, ZNF485, KBTBD12, ALDH18A1, SEL1L3, CDX2, EEF2, SPINK1, CTRL, FKBP11, HCN1, PDZK1, ABCC6, VGF, MUC6, AMH, AADAC, SLITRK1, PTPRN2, NPRL2, ZNF33B, TMED3, DNAH5, DAP, SLC35G1, CHGB, DAGLA, ERRFI1, CXXC4, AGRN, SHANK2, MTUS2, TRIM50, FLT3, OGFOD2, KCNJ11, CDH23, SEL1L, NUCB2, CHDH, HOMER2, DNAJC22
Pituitary : CPT1C, CADM2, ARHGEF33, CNTNAP2, BPIFB3, SLC10A4, OLFM1, LRRC43, CHGB, ZBTB20, FBXO10, WNT1, WNT10B, HCN1, KCNK1, RIMS1, NAALAD2, GPRIN1, NISCH, SORCS3, SULT4A1, NSF, AIFM3, SLITRK1, SERPIND1, SLC7A4, P2RX6, LZTR1, ZDHHC11, GTF2IRD1, SLC39A12, NRXN1, ZNF382, GPR149, VGF, CDH7, MAPT, DCAF4L1, MPO, BEND4, UNC13C, PDE6B, PPP2R2B, SLC4A10, FAM184A, GABRB3, YPEL4, DIO2, AMH, UCHL1, GABRG1, ZNF74, KLHL32, AP3B2, CELSR3, SLC45A1, MTUS2, PCDHAC1, C10ORF67, DPCD, TSNAXIP1, ABCA7, MYT1L, PRR7, DYNC2H1, SPAG17, C4ORF48, NAT8L, CPEB1, MXD4, SLC24A2, COL9A2, NR4A2, FBN3, PTPRN2, DRD2, DCC, DNAH5, HCN4, SLC27A2, DLEC1, FSIP1, ALPK3, NRCAM, ABCA2, PIWIL1, RIMBP2, FGF17, SYT17, AGBL4, MCHR1, ADCY2, RAB19, CACNA1D, FAM153B, TMCC2, CRHR1, ANKRD45, DNAH7, KCNH3, FAXC, NXPH1, KCNC3, ZNF536, CNTNAP4, TERF2IP, COL6A6, CXXC4, KCNIP4, ANKRD34C, ABCB9, STON2, ERC2, OBSCN
Prostate : KLHL5, SEMA3E, ASTN2, CNTNAP2, BEND4, FAM153B, RASL11A, P2RX2, AFF3, ZNF613, DPP4, FBN3, PDE9A, DNAH5, SYCE1L, HOMER2, YJEFN3, OPRK1, SLC23A1, NEB, TMEM200B, ADCY2, SYT17
Skin : CXCL14, PLA2R1, TANC1, SGPP2, PRB1, FN1, FBN2, DLX6, PLEK2, STARD5, EFNA5, POSTN, BNC1, SPINK5, SPPL3, KDM5B, CAMK1D, ETV7, EMB, SLC6A2, CLCA2, EGFR, TNFRSF13C, PRODH, WNT3, AQP5, IGSF3, PRSS3, RASGRP1, SH3BP2, GPLD1, GPR157, TNN, CDHR1, FLG, BCL11A
Stomach : SPINK1, GEM, CAPN6, POSTN, CHST5, SEL1L3, HRH2, PCDH7, THBS1, METTL24, CHRNA7, AADAC, PTPRN2, MUC6, SOWAHA, HOMER2, AQP5, HDC, TRIM50, TROAP, KCNJ11, HS3ST4, EXOC3L4, CKB
Testis : ARHGEF33, BPIFB3, ZAR1, CLTCL1, LRRC43, B3GNT4, PIAS4, ZBTB20, CDC45, GRIN2A, WNT1, OVCH1, NAALAD2, SULT4A1, WNT3, CTNNA3, SLC7A4, P2RX6, CSMD1, MPL, BAG5, CPNE5, C12ORF54, CLCN2, PLCH1, MEX3B, MUC16, DCAF4L1, WDR62, BEND4, CEP85L, RIMBP3, TBC1D3H, FAM184A, MDGA2, FSCB, KIF24, AMH, KCNH7, FSIP1, GSC2, STAB2, CELSR3, CPS1, WBP2NL, FKBP6, PCDHAC1, TRIM50, SYCE1L, CALN1, POLN, C10ORF67, ZC2HC1C, POTEA, ZNF560, DPCD, ASTN2, MEI1, MYT1L, SENP1, BNC1, PIWIL3, TSPYL5, SPAG17, C4ORF48, NAT8L, CPEB1, KDM5B, DCC, HCN4, YJEFN3, KIF20B, AQP5, GPR37, DNAH10, OPRK1, DLEC1, GRK4, RFWD3, CSMD3, PIWIL1, DGCR6L, FBN2, OR56A1, DLX6, IGF2BP1, FAM153B, CHRNB4, IQCK, OPRM1, ANKRD45, DNAH7, LY6G6F, SLC6A2, CNTNAP4, CABLES2, DNAJC18, SPATA24, SLCO6A1, TSNAXIP1, CENPJ, DHX57, DNAH3, TBX1, ABCB9, TROAP, C1QL4, PRPH, POU4F2
Thyroid : CACNA1I, LY75, PLA2R1, DPP6, PLCH1, RAB19, PLEK2, EPHB2, SELE, HIRA, LRRC43, IQCK, STON2, GABRB3, DNAH7, TSHR, EMB, DIO2, ABCC1, CCDC85A, CHRNA7, KCNC3, DNAH11, FBN3, RELN, LMO3, CENPJ, PDE6B, STAB2, CNTN6, AGRN, ARVCF, SHANK2, WWOX, TBX1, C1QL4, TNN, PAPOLG, FGF17, DNAJC22
Uterus : PLA2R1, CPT1C, MCHR1, STARD9, LOXL1, DBN1, MEX3B, URB1, CAPN6, SLC24A2, KLHL38, DPP6, WNT5A, PCDHB12, RTN4R, GRIN2A, YPEL4, METTL24, RERE, SLC6A2, COL4A2, MUC6, ASPN, PTPRU, MATN1, SDC3, PDLIM7, SORCS3, COL5A1, ESR1, LINGO1, SERPIND1, CLDN5, KIF26B, ADAMTS6, HTRA3, COL6A3, TMEM200B, ABCG8, PLEKHH2, AEBP1
Vagina : PRSS3, SGPP2, BHLHE40, MEX3B, CALN1, STARD5, BNC1, CRHR1, SPINK5, SPAG17, WNT5A, GPR157, CSMD3, DIO2, PI16, CLCA2, CLCA4, ADM, MATN1, GPR63, COL5A1, ESR1, IGSF3, SCN5A, BCL11A, TMEM200B, C1QL4, PRPH, PIM1, HS3ST4, ZNF681, NCKAP5, C12ORF54

The overlapped genes for every tissue (pSI threshold 0.01 **) are:**
Adipose Tissue : NAT8L, TNN, FFAR3, KLB
Adrenal Gland : NXPH1, CHRNB4, DNAH11, PLEK2, SLC23A2, CHST6, NRCAM, CALN1, MGST1, SDC3, CHGB, CCDC134, PHOX2B, CNTN6, SLC6A2, AADAC
Blood : FFAR3, PLEK, GCA, BST1, DYSF, HRH2, KCNH7, LY6G6F, LAPTM5, SIGLEC14, DOK3, FPR1, FPR2, TNFRSF13C, MPO, MGAM, DPEP2
Blood Vessel : FN1, PCDH7, LTBP1, COL4A2, COL8A1, ASPN, AEBP1
Brain : GPR149, AGBL4, WNT10B, NLGN3, CACNA1I, CDH7, POU3F2, SLC39A12, RELN, PPP1R16B, MAPT, RASGEF1A, DLX6, CALN1, MYT1L, CNTNAP2, ADAM23, SULT4A1, NXPH1, MCHR2, UNC13C, PDE6B, UCHL1, NAT8L, TMCC2, PPP2R2B, CHGB, ERC2, CHST6, RTN4R, OLFM1, TBR1, GABRB3, SLC24A2, DDN, KCNH3, MDGA2, PTPRN2, HCN1, RASGRP1, WNT1, VGF, CADM2, ZNF536, GABRA2, GABRG1, SOWAHA, NRXN1, DCC, KCNIP4, SLC4A10, CNTNAP4, RIMS1, GPR37, NPAS3, GPRIN1, RBFOX1, SORCS3, KLHL32, NGEF, AP3B2, YJEFN3, PCDH11X, CNTN6, AIFM3, SLITRK1, LINGO1, GRIN2A, YPEL4, OPRK1, ANKRD34C, NRCAM, CNTNAP5, ADCY2, CSMD1, CSMD3, DPP6, HS3ST4, DRD2, FGF17, CMTM5
Breast : CX3CL1, TNN
Colon : AIFM3, GCG, CLCA4, CLCA1, CDX2, PDE9A, DHRS11, SOWAHA, PRSS3, CDHR1, EPHB2, SLC26A2, PHOX2B, CHST5, GAL3ST2
Esophagus : CLCA2, SPINK5, CLCA4, P2RX2, FLG
Fallopian Tube : CAPN6, RASL11A, AEBP1
Heart : CTNNA3, MTUS2, PAM, SCN5A, TTN, PPP1R3A, KLHL38, VWC2, FHL2, HCN4, MDH1, ADAM23, KBTBD12, CSDC2
Kidney : DPP4, HPD, IGF2BP1, IL17RB, NAPSA, CHDH, WWC1, SLC23A1, SLCO4C1, PDZK1
Liver : SERPIND1, HPD, AADAC, SLC27A2, ABCG8, MGST1, KLB, APOM, SLC23A1, EXOC3L4, STAB2, GPLD1, CPS1, DNAJC22, ABCC6
Lung : FPR3, FPR2, DNAH3, FMO2, IGSF10, ITGB6, NAPSA, FPR1, LY6G6F, SLCO4C1, MRC1, PCDH17, COL6A6, MPO, SLC6A4, NCKAP5
Muscle : MACROD1, CLTCL1, P2RX6, TBX1, CACNA1S, ALPK3, NEB, TTN, PPP1R3A, KCNJ11, ADCY2, YIPF7, DDN, RBFOX1, PFKM, ASB8, OBSCN, KBTBD12, KLHL38, DGCR6
Nerve : FMNL3, SLITRK1, RASGEF1A, ZNF536, RELN, CLCN2, CADM2, CMTM5, ABCA2, CDH7, NRXN1, HCN1, PDGFRL, POU3F2, NEGR1
Ovary : FAM153B, SGCZ, IGSF10, SERPINE2, NR4A2, CDH23, VWC2, FHL2, NLGN4X, PCDH11X, ROBO3
Pancreas : GCG, MUC6, ICA1, CDX2, SPINK1, CTRL, PRSS3, SEL1L, SLC43A1, TRIM50, FKBP11, HOMER2
Pituitary : GPR149, AGBL4, CPT1C, CDH7, CHGB, RAB19, MYT1L, ANKRD34C, BPIFB3, SLC10A4, CRHR1, NXPH1, SPAG17, C4ORF48, CPEB1, GABRB3, DNAH7, KCNH3, COL9A2, VGF, AMH, UCHL1, PTPRN2, DRD2, DNAH5, NAALAD2, GPRIN1, TSNAXIP1, SORCS3, COL6A6, SULT4A1, AP3B2, CELSR3, CXXC4, SLITRK1, ABCA7, DLEC1, MTUS2, ZDHHC11, CACNA1D, SLC39A12, ERC2, PIWIL1, RIMBP2, FGF17, SYT17
Prostate : CNTNAP2, SEMA3E, OPRK1, P2RX2, AFF3
Skin : AQP5, CXCL14, SPINK5, FBN2, CLCA2, PRB1, CDHR1, BNC1, WNT3, FLG, BCL11A
Stomach : TRIM50, SPINK1, MUC6, HDC
Testis : MUC16, ZBTB20, TSNAXIP1, OR56A1, PLCH1, AMH, MEI1, MEX3B, MYT1L, DCAF4L1, BPIFB3, BNC1, BEND4, ZNF560, PIWIL3, SLC7A4, CHRNB4, LRRC43, B3GNT4, RIMBP3, OPRM1, CDC45, ANKRD45, PRPH, DNAH7, WNT1, MDGA2, LY6G6F, FSIP1, SLC6A2, FSCB, KIF24, WDR62, DCC, KCNH7, POU4F2, SYCE1L, FAM153B, SPATA24, SLCO6A1, HCN4, SPAG17, GSC2, SULT4A1, CPEB1, IGF2BP1, WBP2NL, AQP5, GPR37, FKBP6, DNAH10, DNAH3, DLEC1, GRK4, TRIM50, CABLES2, CSMD1, TROAP, C1QL4, CSMD3, PIWIL1, DLX6, C10ORF67, ZC2HC1C, POTEA, C12ORF54
Thyroid : CACNA1I, SELE, PLA2R1, FBN3, KCNC3, HIRA, TSHR, CENPJ, DIO2, LMO3, CNTN6
Uterus : ESR1, DPP6, ASPN, TMEM200B, ABCG8, SLC24A2, CAPN6, PLEKHH2, COL5A1
Vagina : CLCA2, ESR1, SPINK5, WNT5A, CLCA4, C1QL4, CSMD3, PI16

The overlapped genes for every tissue (pSI threshold 0.001 **) are:**
Adipose Tissue : none
Adrenal Gland : NXPH1, PHOX2B, SLC6A2, AADAC
Blood : FPR2, DOK3, FPR1, LY6G6F, MPO, MGAM
Blood Vessel : LTBP1, COL8A1
Brain : GPR149, CADM2, MYT1L, CNTNAP2, SLC24A2, NXPH1, UNC13C, TBR1, NAT8L, ERC2, SLC4A10, MCHR2, GRIN2A, DDN, GABRA2, GABRG1, NRXN1, SOWAHA, CNTNAP4, RIMS1, RBFOX1, KLHL32, SULT4A1, POU3F2, SLITRK1, GPR37, OPRK1, CNTNAP5, CALN1, CSMD3, SLC39A12, HS3ST4
Breast : none
Colon : CLCA4, CHST5, CDX2, CLCA1, SLC26A2, GAL3ST2
Esophagus : none
Fallopian Tube : none
Heart : SCN5A
Kidney : PDZK1
Liver : ABCG8, SERPIND1, CPS1
Lung : NAPSA, SLC6A4
Muscle : CACNA1S, NEB, KLHL38, TTN
Nerve : CDH7
Ovary : IGSF10
Pancreas : GCG, PRSS3, SPINK1
Pituitary : GPR149, COL6A6, VGF, COL9A2, DRD2, RIMBP2, MYT1L, CHGB, BPIFB3, SLC10A4, CELSR3
Prostate : none
Skin : CDHR1, PRB1, FLG
Stomach : TRIM50, MUC6
Testis : ZNF560, MEI1, DLX6, DCAF4L1, BPIFB3, BNC1, BEND4, OR56A1, PIWIL3, CHRNB4, GSC2, RIMBP3, OPRM1, CDC45, ANKRD45, MDGA2, FSCB, WDR62, DCC, POU4F2, SLCO6A1, HCN4, IGF2BP1, WBP2NL, AQP5, FKBP6, DNAH3, C1QL4, PIWIL1, MEX3B, C10ORF67, POTEA, C12ORF54
Thyroid : DIO2, KCNC3, TSHR
Uterus : CAPN6
Vagina : CLCA2, SPINK5

The overlapped genes for every tissue (pSI threshold 0.0001 **) are:**
Adipose Tissue : none
Adrenal Gland : NXPH1, PHOX2B
Blood : none
Blood Vessel : none
Brain : SLITRK1, UNC13C, MYT1L, TBR1, SLC4A10, GABRG1, CALN1, MCHR2, SLC24A2, CNTNAP4, SLC39A12, HS3ST4, SULT4A1, POU3F2
Breast : none
Colon : CLCA4, CHST5, CLCA1, CDX2
Esophagus : none
Fallopian Tube : none
Heart : none
Kidney : none
Liver : none
Lung : NAPSA, SLC6A4
Muscle : NEB
Nerve : none
Ovary : none
Pancreas : none
Pituitary : none
Prostate : none
Skin : FLG
Stomach : none
Testis : PIWIL3, GSC2, ZNF560, RIMBP3, OPRM1, OR56A1, POU4F2, SLCO6A1, PIWIL1, BPIFB3, POTEA, FSCB
Thyroid : TSHR
Uterus : none
Vagina : none
