# Supplementary material for: Molecular Landscape of Tourette’s Disorder
Source: Int J Mol Sci. 2023 Jan 11;24(2):1428. doi: 10.3390/ijms24021428 (PMC9865021; doi:10.3390/ijms24021428)
Supplement: Supplementary file 1 [file ijms-24-01428-s001.zip › supplementary File S3.pdf]

Of the inputed 872 genes,
624 genes exist in the brain cell type expression dataset, 248 genes do not, which are:
DNAH10,DNAH11,TLCD4,CKB,NELFA,NDUFB1,BPIFB3,LRRC43,PRB1,GPR75-ASB3,CDK2AP1,ZNF74,CEP128,CERS5,ZNF382,PPP1R3A,NAPSB,FAM153B,TMEM191A,DGCR5,BCDIN3D-AS1,HNRNPLL,ZNF260,P3H2,ZNF577,UFD1,TMEM178B,RP1L1,ZNF664,MAPK3,EIF4EBP2P3,PCDH11X,SLC10A4,KMT5A,TRIQK,ZNF681,MUC6,LINC02694,XYLT1,DLEC1,SIGLEC14,SNU13,IQCK,FAXC,KIRREL1,SLC35G1,MARCHF7,SPRY3,KCNMB3,TLNRD1,VPS9D1-AS1,SAXO2,ST20,HCN4,OVCH1,EFL1,STARD9,RAB42,SPINK1,MTRFR,ZNF567,MPIG6B,OFCC1,ASS1P2,CPS1,ZNF560,SNORC,COPS9,HMGN2,AHCY,TBC1D3H,TMEM170A,EPB41,ICE1,GFUS,TANGO2,OR56A1,RTL10,HEXA-AS1,RNA5SP172,YPEL4,ZNF169,ST20-MTHFS,POM121L4P,TUBA1C,TUBA1B,TUBA1A,KMT2D,RAMAC,IGSF10,ZNF385D,PCDHAC1,DGCR6L,KAT14,ARIH2OS,PALM2AKAP2,ATP5IF1,URAD,MAU2,C1QL4,ARHGEF33,CACNA1I,MPV17,ZNF568,ZC2HC1C,KLHL38,GTF2IRD1,ZNF581,C22ORF39,ADAMTS6,C2ORF49,OR52A5,SNORA79,HYKK,PCDHGA2,CEP295NL,GPR89A,ZNF485,CHST6,DNAH3,ZNF613,DNAH1,DNAH7,RBFOX1,DNAH5,SAMD11,RNU6-1228P,LRATD1,CFAP99,LY75,CLTCL1,PCDHGA9,STING1,DCAF4L1,NSD3,WDPCP,SLCO6A1,DPEP2,CEP85L,C12ORF54,PIWIL3,IHO1,GPR75,TENM2,HS3ST4,ZNF837,TENM4,P2RX6P,DYNC2I1,TRIM50,CORO7-PAM16,ZNF536,ESS2,PI4KAP1,SRSF7,SLC49A3,CFAP251,LY6G6F,TMEM200B,MRTFA,BOLA3P2,SGCZ,OR8T1P,RIMS1,SUGP1,NOTCH2NLA,SPATS2,RIMBP2,RPS21,ZNF83,COMT,ANKRD34C,NUDT12,ENO1,PEX2,ZNF518B,CHMP1A,C4ORF48,FSCB,CAMKMT,HSP90AA1,GPR157,ETV7,ZNF529,LMO3,GEMIN6,C10ORF67,LY6G5B,MIR2113,SOWAHA,EXOC3L4,ZNF33B,SEPTIN5,METTL24,THEM7P,INSYN1,OR56B4,C20ORF204,KANSL1,IGIP,MARF1,DESI1,ABHD16A,MCRS1,CNTNAP5,PROB1,GAL3ST2,MICU3,LY75-CD302,RERE,GOLGA6L9,GABARAPL2,MINAR1,PRR7,SELENOK,ZNF557,ACAA1,COP1,DTX2P1,MPPE1,ADRM1,SELENOF,POTEA,KMT2C,COA8,SRSF4,NLGN4X,FBN3,MCHR2,MORF4L2,PIGW,FFAR3,SHANK2,GPR42,MESD,ZNF461,SRSF3,VAMP7,ST20-AS1,DOP1B,CIBAR1,DRD2,DGCR11,DCAF1,GRIN2A,KARS1,ZNF799,COL6A6

Interpretation of CSEA results table:
The first row lists the pSI thresholds, and the first column lists the brain cell types
The values in the tables are the Fisher's Exact p-values, followed by the Benjamini-Hochberg (BH) corrected values in parentheses.
Click the linked p-values to see the overlapped genes for the corresponding brain cell types and thresholds.

Brain Cell Types and P-Values	0.05	0.01	0.001	0.0001
RetR.Rods	0.678 (0.999)	0.834 (1.000)	0.942 (1.000)	0.849 (1.000)
Hyp	0.997 (0.999)	0.943 (1.000)	1.000 (1.000)	1.000 (1.000)
Hyp.Hcrt	0.999 (0.999)	0.981 (1.000)	0.951 (1.000)	0.871 (1.000)
BF	0.632 (0.999)	0.877 (1.000)	1.000 (1.000)	1.000 (1.000)
BF.Chat	0.743 (0.999)	0.796 (1.000)	0.575 (1.000)	0.273 (1.000)
BS	0.517 (0.999)	0.272 (1.000)	0.039 (1.000)	1.000 (1.000)
BS.Chat	0.594 (0.999)	0.815 (1.000)	0.638 (1.000)	0.284 (1.000)
BS.Slc6a4	0.495 (0.999)	0.741 (1.000)	0.793 (1.000)	0.681 (1.000)
Cb	0.948 (0.999)	0.998 (1.000)	1.000 (1.000)	1.000 (1.000)
Cb.Septin4	0.729 (0.999)	0.854 (1.000)	0.884 (1.000)	0.927 (1.000)
Cb.Pcp2	0.726 (0.999)	0.768 (1.000)	0.752 (1.000)	0.984 (1.000)
Cb.Neurod1	0.810 (0.999)	0.764 (1.000)	0.858 (1.000)	1.000 (1.000)
Cb.Lypd6	0.503 (0.999)	0.719 (1.000)	0.683 (1.000)	0.829 (1.000)
Cb.Grp	0.998 (0.999)	1.000 (1.000)	1.000 (1.000)	1.000 (1.000)
Cb.Grm2	0.495 (0.999)	0.625 (1.000)	0.489 (1.000)	0.555 (1.000)
Cb.Fthfd	0.942 (0.999)	0.948 (1.000)	0.979 (1.000)	1.000 (1.000)
Cb.Cnp	0.999 (0.999)	0.915 (1.000)	0.829 (1.000)	0.719 (1.000)
Cpu	0.151 (0.999)	0.304 (1.000)	1.000 (1.000)	1.000 (1.000)
Cpu.D2	0.007 (0.160)	0.048 (0.836)	0.588 (1.000)	0.442 (1.000)
Cpu.D1	0.441 (0.999)	0.285 (1.000)	0.732 (1.000)	1.000 (1.000)
Cpu.Chat	0.192 (0.999)	0.526 (1.000)	0.349 (1.000)	0.442 (1.000)
Ctx	0.428 (0.999)	0.519 (1.000)	1.000 (1.000)	1.000 (1.000)
Ctx.Pdgfrajd340	0.992 (0.999)	0.992 (1.000)	0.993 (1.000)	1.000 (1.000)
Ctx.Etv1_ts88	0.492 (0.999)	0.715 (1.000)	0.358 (1.000)	0.519 (1.000)
Ctx.Pnoc	0.264 (0.999)	0.081 (0.943)	0.246 (1.000)	1.000 (1.000)
Ctx.Ntsr	0.009 (0.160)	0.812 (1.000)	1.000 (1.000)	1.000 (1.000)
Ctx.Glt25d2	0.067 (0.786)	0.035 (0.836)	0.120 (1.000)	1.000 (1.000)
Ctx.Fthfd	0.984 (0.999)	0.823 (1.000)	0.872 (1.000)	1.000 (1.000)
Ctx.Cort	0.122 (0.999)	0.486 (1.000)	0.555 (1.000)	1.000 (1.000)
Ctx.Cnp	0.992 (0.999)	0.996 (1.000)	0.886 (1.000)	0.879 (1.000)
Epi	0.702 (0.999)	0.880 (1.000)	0.903 (1.000)	1.000 (1.000)
Epi.Chat	0.758 (0.999)	0.544 (1.000)	0.363 (1.000)	0.106 (1.000)
Spc	0.970 (0.999)	0.978 (1.000)	1.000 (1.000)	1.000 (1.000)
Spc.Chat	0.794 (0.999)	0.925 (1.000)	0.969 (1.000)	0.820 (1.000)
RetC.Cones	0.828 (0.999)	0.862 (1.000)	0.973 (1.000)	0.860 (1.000)

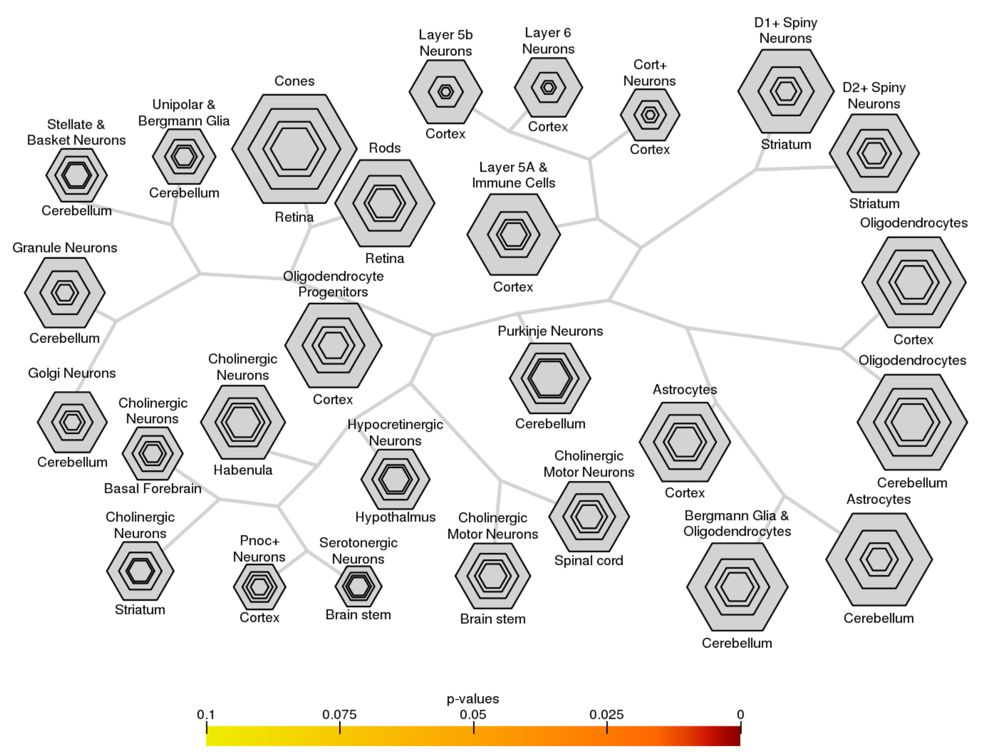

The overlapped genes for every cell type (pSI threshold 0.05 **) are:**
RetR.Rods : STON2, SF3A2, NCKAP5, SLC26A2, FAM193B, PDE6B, FAM184A, PIAS3, TCOF1, TOB2, CELSR3, ZFYVE28, TNIP2, GABPB1, DLC1, ABCA7, KDM5B, MYO10, DYNC2H1, POU4F2, URB1, PLCH1, PIAS4, PLA2R1
Hyp : HDC, DGKQ, GABRG1, FAM193B
Hyp.Hcrt : NUCB2, PI16, VGF, TMED3
BF : AKAP9, GPR149, OPRK1, POSTN, NCL, PROKR2, PCDH17, DLX6, BIRC6, DDN
BF.Chat : ZAR1, SLC25A1, GSC2, OPRD1, UCHL1, CDKN1A, YJEFN3, TIMP2
BS : MGST1, BAZ2B, POU4F2, SLC27A2, SLC6A4, FAM184A, CDH7, CSMD3, PRPH, PHOX2B, HOXB4
BS.Chat : ZAR1, KCNC3, P2RX6, FLT3, NXPH1, EMB, GEM, CPEB1, ADAM23, SLC45A1, TM6SF1, PRPH, PHOX2B, HOXB4
BS.Slc6a4 : ZAR1, SLC27A2, SLC6A4, STARD3NL, PRPH, VWC2
Cb : RELN, FLT3, ATN1, ZC3H7B, FAM193B, MPHOSPH9, CRHR1, UNC13C, TRIO, CADPS2, SEL1L3, HCN1, PDGFRL, POLD1, SGPP2, SERINC2, PAN3
Cb.Septin4 : LIX1L, PDLIM7, NCKAP5, TEAD2, RASL11A, MXD4, COL9A2, PREX1, MGST1, NKX2-2, ABCB9, HTRA3, KLHL5, GPLD1, CMTM5, CXCL14, SLC39A12, TRIP6, CD302, PRODH, DIO2, CBR3, SLC25A17, CDKN1A
Cb.Pcp2 : IMMP2L, HDC, FLT3, DAGLA, PPP1R16B, RIMBP3, ARHGAP26, NINL, TM6SF1, CSDC2, PDE9A, KCNIP4, DAP, BCL11A, SERINC2
Cb.Neurod1 : RTN4R, ARHGEF3, PAN3, DHRS11, SENP1, KIF26B, CRHR1, TOB2, NRXN1, KCNH3, UNC13C, CADPS2, SEL1L3, COL9A2
Cb.Lypd6 : EPHB2, SORCS3, STARD5, PAM, HCN1, MACROD1, CSDC2, SGPP2, PDE4A, PLCH1
Cb.Grp : CMTM5, TEAD2, MGST1
Cb.Grm2 : IMMP2L, PTPRN2, ST3GAL3, WWC1, FKBP6, RANBP1, PAM, SEMA3E, KCNH3, ZCRB1, TCF20, EFNA5, VWC2
Cb.Fthfd : LIX1L, RASL11A, MXD4, PREX1, MGST1, NKX2-2, ABCB9, HTRA3, KLHL5, RMND5B, GPLD1, NPAS3, CMTM5, CXCL14, SLC39A12, TRIP6, CD302, PDGFRL, PRODH, DIO2, SDC3, CDKN1A
Cb.Cnp : WNT3, NFE2L3, SORT1, GPR37, CHDH, ABCA2, PPP1R16B, LY6G6D, CMTM5, NIPA1, MON1B, DIABLO, NKX2-2, IL17RB, SERPIND1, STARD3NL, SH3BP2
Cpu : RIMBP3, AKAP9, DBN1, ANK3, POSTN, CPNE5, FOXP1, DLX6, INO80D, DCC, MYT1L, NUCB2, CSMD3, DDN, RUNX1T1, NGEF
Cpu.D2 : ME2, UNC13C, TEF, PAM, RASGEF1A, ASH1L, PPP1R16B, MCHR1, NGEF, ZFYVE28, XPNPEP3, POSTN, ARHGEF3, AKAP9, CALN1, ZC3H7B, GPR149, RIMBP3, CPNE5, AFF3, MYT1L, CSMD3, SEMA3E, RAB11FIP3, CACNA2D3, CBR3, RUNX1T1, TRMT61A, PHF14, TUBGCP5, FOXP1, DLX6, DDN
Cpu.D1 : ARF3, UNC13C, TEF, TRMT61A, ASH1L, PPP1R16B, PIKFYVE, MCHR1, RASGRP1, NGEF, ZFYVE28, RASGEF1A, CALN1, ZC3H7B, POSTN, CPNE5, LMO4, MYT1L, CSMD3, RAB11FIP3, CBR3, TUBGCP5, FOXP1, DLX6, DDN
Cpu.Chat : DGCR6, ZAR1, SLC25A1, DLX6, GSC2, OPRD1, UCHL1, CRHR1, ADCY2, CXCL14, RGS12, NARS2, HRH2, FHIT, TIMP2
Ctx : LMO4, OTUD1, RNF169, GPRIN1, LINGO1, NGEF, EFNA5, FHL2, TBR1, RASGEF1A, DDN
Ctx.Pdgfrajd340 : LTBP1, MEX3B, NPAS3, NCKAP5, NKX2-2, RIMBP3, ADM, HTRA3, DCAF12, KLHL5, CHST5, SERPIND1, PHF14, PREX1
Ctx.Etv1_ts88 : RNF169, CX3CL1, SYT17, RASGRP1, NGEF, LZTR1, TBR1, AP3B2, MRC1, KCNH3, SLC39A12, LINGO1, SH3BP2, PRODH, LAPTM5, SLC7A4, PAM, GPRIN1, RTN4R, PIAS4, KALRN, SORCS3, FHL2
Ctx.Pnoc : NXPH1, YJEFN3, AQP5, WNT5A, CXCL14, RGS12, SESN2, ANKRD45, VWC2
Ctx.Ntsr : RELN, RTN4R, RASGRP1, IFIH1, CDHR1, OTUD1, SEMA3E, NR4A2, PCDHB12, SYT17, SLC4A10, NRXN1, PROKR2, VPS13A, NOTCH1, LINGO1, BCL11A, SLCO4C1, KALRN, TBR1, SORCS3
Ctx.Glt25d2 : ABCF2, LMO4, OPA1, PCDHB12, SYT17, MCHR1, PAM, MRC1, TEK, SLC7A4, CLDN5, LINGO1, BHLHE40, RASGRP1, SLCO4C1, CX3CL1, GPRIN1, IGSF3, SORCS3, FHL2
Ctx.Fthfd : MYO10, LTBP1, DIO2, MGST1, CBR3, GEM, CXCL14, SLC39A12, TRIP6, POLR3H, GABRG1, DUSP6, PRODH
Ctx.Cort : FBXO10, KALRN, ROBO3, RNF169, SLC4A10, GPRIN1, KCNH3, SLC23A2, PLA2R1, WBP2NL, NLGN3, VWC2, KCNH5
Ctx.Cnp : WNT3, NFE2L3, GPR37, CHDH, ABCA2, PPP1R16B, SORT1, CMTM5, SYNJ2, GCA, NIPA1, SLC29A3, TBC1D5, NKX2-2, SERPIND1, IL17RB, COL6A3
Epi : NXPH1, HDC, ROBO3, SYT17, CHRNB4, PROKR2, HOMER2, ARVCF, MAP3K6, RASL11A, BIRC6, TANC1
Epi.Chat : CHRNB4, NR4A2, ROBO3, SYT17, PHF14, YJEFN3, RTN4R, CADPS2, MAP3K6, PRKAR2A, TNFAIP2, OBSCN, AIFM3, DAP, SEMA3E, DPP6
Spc : GPLD1, NFE2L3, FLT3, MGST1, CHDH, ABCA2, SLC27A2, FN1, NAT8L, WNT5A, SLC39A12, FAM193B, SERPIND1, CSMD3, PRPH, SGPP2, HOXB4
Spc.Chat : CDHR1, NXPH1, DPCD, CCDC92, P2RX6, ABCA7, SLC25A1, SLC45A1, CPEB1, ADAM23, NUDT17, PRPH, VWC2, FLT3
RetC.Cones : OPA1, ZDHHC17, KLC1, TEF, RAB18, ST3GAL3, FARP2, PKN2, DIO2, TLK1, SEL1L, BEND4, PREPL, CDHR1, RICTOR, LMBR1L, PCM1, CACNA2D3, GEM, CHRNB4, TEK, HCN1, COL4A2, XRN2, CACNA1D, GLE1, GSC2, CACNA1S, DBN1, BAZ2B, CLDN5, FN1, SLC23A2, RRM1, CCDC66

The overlapped genes for every cell type (pSI threshold 0.01 **) are:**
RetR.Rods : TCOF1, DYNC2H1, POU4F2, PLA2R1, PLCH1, PDE6B, FAM184A, SLC26A2
Hyp : HDC
Hyp.Hcrt : PI16, VGF
BF : OPRK1
BF.Chat : OPRD1, ZAR1, SLC25A1
BS : POU4F2, SLC6A4, HOXB4
BS.Chat : PRPH, P2RX6, FLT3, PHOX2B, HOXB4
BS.Slc6a4 : ZAR1, SLC6A4
Cb : SERINC2
Cb.Septin4 : MGST1, CMTM5, HTRA3, TEAD2, CDKN1A, RASL11A, TRIP6, PREX1
Cb.Pcp2 : HDC, DAGLA, NINL, TM6SF1, PDE9A, DAP, SERINC2
Cb.Neurod1 : UNC13C, CRHR1, CADPS2, DHRS11, SEL1L3
Cb.Lypd6 : STARD5, HCN1, PLCH1, SORCS3
Cb.Grp : none
Cb.Grm2 : EFNA5, FKBP6, KCNH3, VWC2
Cb.Fthfd : NPAS3, MGST1, ABCB9, HTRA3, SLC39A12, TRIP6, PREX1
Cb.Cnp : WNT3, NFE2L3, SORT1, GPR37, CHDH, ABCA2, NKX2-2, LY6G6D, CMTM5, SERPIND1, IL17RB
Cpu : FOXP1, POSTN, AKAP9, NGEF
Cpu.D2 : RAB11FIP3, AKAP9, ASH1L, GPR149, RIMBP3, CPNE5, MCHR1, FOXP1, DLX6, TRMT61A, CSMD3, RASGEF1A, DDN
Cpu.D1 : RAB11FIP3, ASH1L, POSTN, CPNE5, FOXP1, TEF, DLX6, NGEF, RASGEF1A, DDN
Cpu.Chat : CXCL14, ZAR1, OPRD1, GSC2, SLC25A1
Ctx : TBR1, FHL2
Ctx.Pdgfrajd340 : NCKAP5, NKX2-2, CHST5, ADM
Ctx.Etv1_ts88 : RNF169, LAPTM5, MRC1, RTN4R, LINGO1, TBR1
Ctx.Pnoc : NXPH1, ANKRD45, AQP5, WNT5A, RGS12, YJEFN3
Ctx.Ntsr : CDHR1, TBR1
Ctx.Glt25d2 : CLDN5, SYT17, SLC7A4, GPRIN1, TEK, MCHR1, SORCS3
Ctx.Fthfd : DIO2, MGST1, CBR3, GEM, CXCL14, SLC39A12, TRIP6, PRODH
Ctx.Cort : KCNH3, KALRN, WBP2NL
Ctx.Cnp : NFE2L3, SORT1, CHDH, GPR37, SERPIND1, IL17RB
Epi : PROKR2, MAP3K6, CHRNB4
Epi.Chat : NR4A2, ROBO3, CHRNB4, RTN4R, TNFAIP2, MAP3K6, OBSCN, YJEFN3
Spc : SLC27A2, PRPH, HOXB4
Spc.Chat : PRPH, P2RX6, FLT3, SLC45A1
RetC.Cones : CDHR1, RICTOR, LMBR1L, PCM1, CACNA1S, FN1, BAZ2B, CLDN5, TLK1, GEM, CHRNB4, TEK, TEF, ST3GAL3, COL4A2, PREPL, SLC23A2

The overlapped genes for every cell type (pSI threshold 0.001 **) are:**
RetR.Rods : PDE6B, PLA2R1
Hyp : none
Hyp.Hcrt : PI16
BF : none
BF.Chat : OPRD1, ZAR1
BS : POU4F2, SLC6A4
BS.Chat : PRPH, P2RX6, PHOX2B
BS.Slc6a4 : SLC6A4
Cb : none
Cb.Septin4 : RASL11A, TRIP6, TEAD2
Cb.Pcp2 : TM6SF1, HDC, DAP, NINL
Cb.Neurod1 : CRHR1
Cb.Lypd6 : STARD5, PLCH1
Cb.Grp : none
Cb.Grm2 : FKBP6, VWC2
Cb.Fthfd : MGST1
Cb.Cnp : NFE2L3, SORT1, LY6G6D, GPR37, SERPIND1, IL17RB
Cpu : none
Cpu.D2 : FOXP1, RAB11FIP3, DDN
Cpu.D1 : FOXP1, RAB11FIP3
Cpu.Chat : OPRD1, ZAR1, GSC2
Ctx : none
Ctx.Pdgfrajd340 : NKX2-2
Ctx.Etv1_ts88 : RTN4R, LINGO1, TBR1, MRC1
Ctx.Pnoc : RGS12, YJEFN3, AQP5
Ctx.Ntsr : none
Ctx.Glt25d2 : CLDN5, SLC7A4
Ctx.Fthfd : SLC39A12, DIO2, PRODH
Ctx.Cort : KCNH3
Ctx.Cnp : SERPIND1, NFE2L3, GPR37, CHDH
Epi : PROKR2
Epi.Chat : NR4A2, ROBO3, CHRNB4, TNFAIP2, MAP3K6, OBSCN
Spc : none
Spc.Chat : PRPH
RetC.Cones : CDHR1, LMBR1L, CACNA1S, GEM, BAZ2B

The overlapped genes for every cell type (pSI threshold 0.0001 **) are:**
RetR.Rods : PDE6B, PLA2R1
Hyp : none
Hyp.Hcrt : PI16
BF : none
BF.Chat : OPRD1, ZAR1
BS : none
BS.Chat : PRPH, P2RX6, PHOX2B
BS.Slc6a4 : SLC6A4
Cb : none
Cb.Septin4 : TRIP6
Cb.Pcp2 : NINL
Cb.Neurod1 : none
Cb.Lypd6 : PLCH1
Cb.Grp : none
Cb.Grm2 : VWC2
Cb.Fthfd : none
Cb.Cnp : SERPIND1, IL17RB, GPR37, NFE2L3
Cpu : none
Cpu.D2 : FOXP1, RAB11FIP3
Cpu.D1 : none
Cpu.Chat : OPRD1, ZAR1
Ctx : none
Ctx.Pdgfrajd340 : none
Ctx.Etv1_ts88 : TBR1, MRC1
Ctx.Pnoc : none
Ctx.Ntsr : none
Ctx.Glt25d2 : none
Ctx.Fthfd : none
Ctx.Cort : none
Ctx.Cnp : GPR37, CHDH
Epi : none
Epi.Chat : NR4A2, ROBO3, CHRNB4, TNFAIP2, MAP3K6, OBSCN
Spc : none
Spc.Chat : PRPH
RetC.Cones : CDHR1, LMBR1L, GEM, BAZ2B
